# The Nitrofuran-Warhead-Equipped Spirocyclic Azetidines Show Excellent Activity against *Mycobacterium tuberculosis*

**DOI:** 10.3390/molecules29133071

**Published:** 2024-06-27

**Authors:** Kristina Komarova, Lyubov Vinogradova, Alexey Lukin, Maxim Zhuravlev, Dmitry Deniskin, Mikhail Chudinov, Maxim Gureev, Marine Dogonadze, Natalia Zabolotnykh, Tatiana Vinogradova, Anastasia Lavrova, Petr Yablonskiy

**Affiliations:** 1 Lomonosov Institute of Fine Chemical Technologies, MIREA—Russian Technological University, 119454 Moscow, Russia; 2 Institute of Cytology, Russian Academy of Sciences, Tikhoretsky Ave 4, 194064 Saint Petersburg, Russia; 3 Saint-Petersburg State Research Institute of Phthisiopulmonology of the Ministry of Healthcare of the Russian Federation, 191036 Saint Petersburg, Russia; 4 Sophya Kovalevskaya North-West Mathematical Research Center, Immanuel Kant Baltic Federal University, 236041 Kaliningrad, Russia; 5 Department of Hospital Surgery, Faculty of Medicine, Saint Petersburg State University, 199034 Saint Petersburg, Russia

**Keywords:** nitrofurans, spiroazetidines, tuberculosis, antimycobacterial activity, induced fit docking, deazaflavin-dependent nitroreductase

## Abstract

A series of 21 new 7′H-spiro[azetidine-3,5′-furo [3,4-d]pyrimidine]s substituted at the pyrimidine ring second position were synthesized. The compounds showed high antibacterial in vitro activity against *M. tuberculosis*. Two compounds had lower minimum inhibitory concentrations against *Mtb* (H37Rv strain) compared with isoniazid. The novel spirocyclic scaffold shows excellent properties for anti-tuberculosis drug development.

## 1. Introduction

*Nitroheterocycles*, *spirocycles*, and *anti-tuberculosis activity* are our lab’s favorite keywords. Nitroheterocyclic compounds can attack many targets in a bacterial cell and therefore represent an extremely effective antibacterial warhead [1,2,3,4,5,6,7]. The mechanism of action of nitroheterocyclic antibiotics, such as nitrofurans and nitroimidazoles, is based on their reduction by bacterial enzymes [8,9]. This reaction leads to the formation of various toxic species that destroy the bacteria’s life cycle [10].

Due to their unique structure and intrinsic three-dimensionality, spirocyclic scaffolds are being used more frequently in drug discovery [11]. The spirocycles also appear to be attractive and privileged structures for antimicrobial drug design [12]. Spirocycles are a significant part of sp^3^-rich scaffolds [13,14], providing opportunities to “escape from flatland,” that is, to increase the drug-likeness of designed substances [15].

Tuberculosis is an old and insidious enemy of mankind [16,17]. Multidrug-resistant strains of *M. tuberculosis* (*Mtb*) are widespread throughout the world [18,19], and the fight against this enemy depends on the development of new antibiotics [20,21].

**Figure 1:**
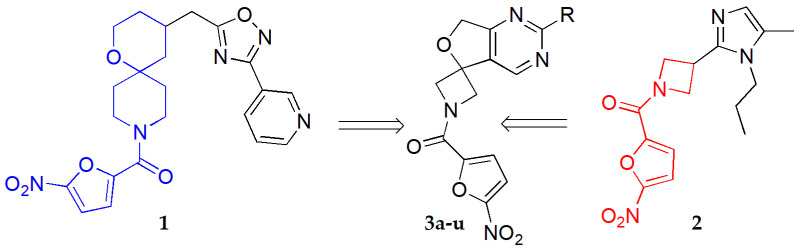
The design of the new compound series.

The design of the new compound series was inspired by our previous successful substances: 9-(5-nitro-2-furoyl)-4-[(3-pyridin-3-yl-1,2,4-oxadiazol-5-yl)methyl]-1-oxa-9- azaspiro [5.5]undecane (**1**) [22] and 5-methyl-2-[1-(5-nitro-2-furoyl)azetidin-3-yl]-1-propyl-1*H*-imidazole (**2**) [23] (Figure 1). Both of these compounds had equal minimum inhibitory concentrations (MICs) of 1.6 μg/mL against the *Mtb* H37Rv strain. We assumed that the combination of two structural elements could lead to the new active entities combining the best properties of two partners in one molecule. Active spirocyclic compounds of the 7′H-spiro[azetidine-3,5′-furo[3,4-d]pyrimidine] chemotype have not previously been described, and their synthesis represented a new and interesting challenge.

Recently, we designed this spirocyclic scaffold containing an azetidine moiety and synthesized four target compounds (**3a**–**d**) [24]. The first compounds synthesized displayed remarkable activity against *Mtb* (HRv37 strain) in a preliminary test. This gave us a reason to expand the range of substituents in search of the best activity and SAR parameters. Thus, our work combines three key elements: spirocycles, nitroheterocycles, and anti-tuberculosis activity.

## 2. Results and Discussion

### 2.1. Chemistry

**Scheme 1:**
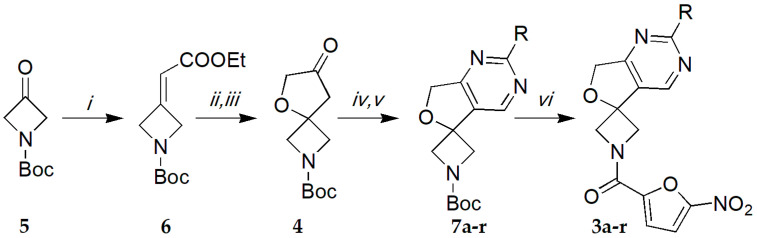
Synthesis of target compounds **3a**–**d**. Reagents and conditions: (i) (EtO)_2_P(O)CH_2_CO_2_Et, NaH, THF, 0° → 20 °C, 18 h (92%); (ii) HOCH_2_CO_2_Me, NaH, Et_2_O; (iii) DMSO, 0 °C, then DMSO/H_2_O/NaCl, 120 °C, 2 h (61%); (iv) Me_2_NCH(OMe)_2_, reflux, 4 h; (v) RC(=NH)NH_2_∙HCl (**7a**–**d**), MeONa, MeOH, 0 °C, then reflux, 18 h (13–84%); (*vi*) CF_3_CO_2_H, CH_2_Cl_2_, 0 °C, 1 h, then DMF/Et_3_N, CDI/5-nitro-2-furoic acid, rt, 18 h (20–60%).

The key synthetic precursor of compounds **3a**–**u** was spirocyclic ketone **4**, whose synthesis was carried out according to the described procedure [25]. The starting tert-butyl 3-oxaazetidine-1-carboxylate **5** by a Horner–Emmons reaction with triethyl phosphonacetate gave the unsaturated ester **6** in a 92% yield. Next, the Michael reaction was carried out with glycolic acid methyl ester, followed by the Claisen condensation and decarboxylation. The yield of spirocyclic ketone **4** after chromatographic purification on silica gel was 61% (Scheme 1).

The ketone **4** was refluxed with an excess of dimethylformamide dimethyl acetal Me_2_NCH(OMe)_2_, and the resulting intermediate, without additional purification, was introduced into a cyclization reaction with the corresponding amidines. The choice of the amidines was determined by their availability and aimed for maximum chemical diversity in the final compounds. Some of the amidines not available commercially were obtained from corresponding nitriles via iminoester hydrochlorides obtained by the classic Pinner reaction [26]. In the case of heteroaromatic nitriles of the pyridine and pyrazine series, the corresponding imino esters were obtained with catalytic amounts of sodium methoxide in absolute methanol. The yields and products of the pyrimidines **7a**–**r** synthesis are presented in Table 1. The key step responsible for the overall synthesis success was the regioselective addition of Me_2_NCH(OMe)_2_ at the C-8 position of spirocyclic ketone **4**. Further heterocyclization with amidines led exclusively to the single regioisomer, with the structure confirmation by NMR. After the removal of the Boc protecting group in compounds **7a**–**r** by trifluoroacetic acid, resulting unstable amines were immediately acylated with 5-nitro-2-furoic acid to the final compounds **3a**–**r**. The yields of the compounds **3a**–**r** are presented in Table 1.

**Scheme 2:**
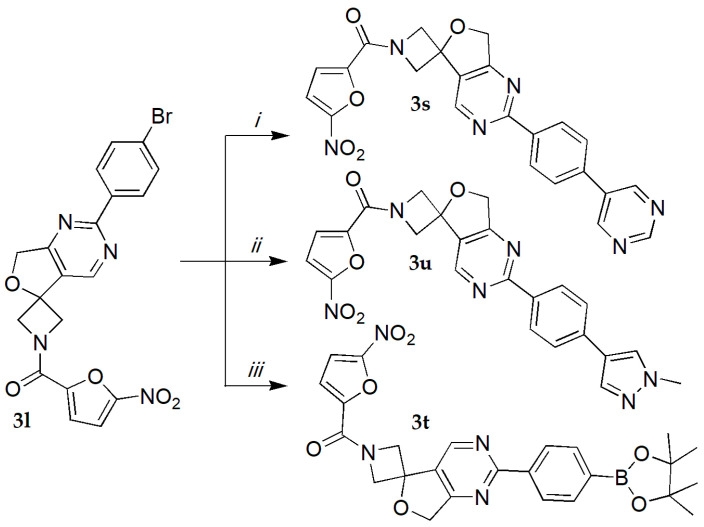
Synthesis of target compounds **3s**–**u**. Reagents and conditions: (*i*) pyrimidine-5-boronic acid, Pd(Dppf)Cl_2_, Cs_2_CO_3_, dioxane/water (10:1), 105 °C, 6 h (50%); (*ii*) 1-methyl-1H-pyrazole-4-boronic acid pinacol ester, Pd(Dppf)Cl_2_, Cs_2_CO_3_, dioxane/water (10:1), 105 °C, 6 h (40%); (*iii*) bis(pinacolato)diboron, Pd(Dppf)Cl_2_, AcOK, dioxane, 105 °C, 6 h (45%).

In the case of complex nitriles with high molecular weight, their use for the amidines’ synthesis can be difficult. We have shown the possibility for further transformation of compound **3l** into final compounds that are not available by Scheme 1. Compounds **3s** and **3u** were obtained by Suzuki cross-coupling using Pd(Dppf)Cl_2_ as a catalyst and cesium carbonate as a base in a water–dioxane mixture (Scheme 2). Compound **3t** was synthesized as an alternative substrate for cross-coupling.

### 2.2. Antibacterial Activity

Compounds **3a**–**u** were tested against MTb (H37Rv strain) by serial dilution, and MICs were determined (Table 2). The clinically used antibiotic isoniazid served as a positive control and a comparator. Most substances in the series demonstrated good anti-tubercular activity. An analysis of the obtained MIC data showed that the substitution in one of the phenyl ring positions of compound **3b** with a halogen atom significantly increased the activity. Compounds **3f** and **3l** with p-halogen-phenyl substituent showed the best activity, exceeding the activity of the comparator. Compounds with heteroaromatic substituents, such as pyridine (**3j**, **3n**, **3o**) or pyrazine (**3p**), were less active than the aromatic ones. In general, a decrease in activity was observed with a decrease in the lipophilicity of the molecules.

### 2.3. In Silico Studies

The primary targets that interact with nitrofurans in the context of tuberculosis are deazaflavin-dependent nitroreductase (Ddn) and NAD(P)H nitroreductase Acg (Rv2032). It is currently not possible to model the interaction of the studied compounds with the Acg protein due to the lack of a molecular basis, despite the availability of the protein structure. Therefore, the interaction of the target compounds with the Ddn protein was modeled only. The binding poses of the ligands were predicted using the induced fit docking method (IFD). IFD docking, a modeling technique that allows the estimation of ligand-induced changes in the receptor structure, is a variation of molecular docking that considers the possible mobility of a receptor upon binding to a small molecule within a given radius (in this case, 6 Å from the ligand) in a limited way. Calculations were performed for a number of substances, including the most active and least active compounds (Table 2).

**Figure 2:**
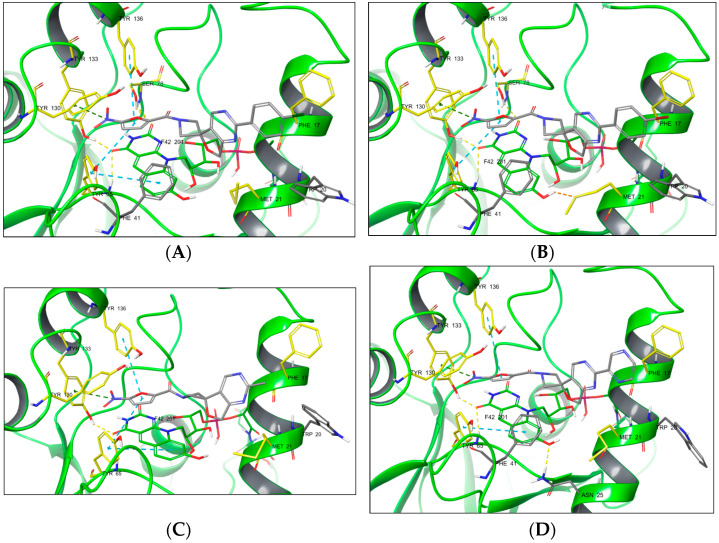
(**A**) Three-dimensional model of ligand binding in the active cavity of Ddn. Dotted lines indicate the following interactions: blue—stacking, green—π-cationic, yellow—hydrogen bonding, orange—strained contacts. (**A**): **3f**@Ddn; (**B**): **3l**@Ddn; (**C**): **3c**@Ddn; (**D**): **3p**@Ddn.

The IFD docking into the active cavity of deazaflavin-dependent nitroreductase yielded more promising outcomes. The estimated function score correlated with the MIC value. Concurrently, the active compounds exhibited a favorable binding pose quality. The nitrofuran moiety of the molecule was correctly oriented in the direction of the catalytic center of the protein and was planar relative to the flavin mononucleotide (FMN). A three-dimensional stacking analysis of compounds **3f** and **3l** (leaders) as well as **3c** and **3p** (least active) revealed the influence of the substituent at the pyrimidine fragment of the scaffold. The halogenphenyl moiety provided the necessary lipophilic contacts with Met21, Trp20, Ile18, and Phe17 (in the case of **3f**, **l**), which ensured an energetically more favorable interaction with Ddn nitroreductase (see Figure 2). Substances within the activity gradient, such as **3d** and related compounds, possessed lipophilic substituents that interacted with the aforementioned lipophilic amino acids to a limited extent.

## 3. Materials and Methods

### 3.1. Chemistry

Commercially available reagents were used without further purification. NMR spectra were recorded using a Bruker DPX-300 spectrometer (^1^H: 300 MHz; ^13^C: 75 MHz). Chemical shifts are reported as parts per million (δ, ppm). The residual solvent peak (CHCl_3_ or DMSO-*d*_6_) was used as the internal standard: 7.28 or 2.51 for ^1^H and 77.07 or 40.00 ppm for ^13^C. Multiplicities are abbreviated as follows: s = singlet, d = doublet, t = triplet, q = quartet, m = multiplet, br = broad, dd = doublet of doublets, dt = doublet of triplets, and ddd = doublet/doublets of doublets (see Supplementary Materials). Coupling constants, *J*, are reported in Hz. Mass spectra were recorded using a Bruker microTOF spectrometer (ionization by electrospray, positive ion detection). Melting points were determined in open capillary tubes using a Stuart SMP50 Automatic Melting Point Apparatus (Palm City, FL, USA). Analytical thin-layer chromatography was carried out on UV-254 silica gel plates. Visualization was accomplished by UV light. Column chromatography was performed with 230–400-mesh silica gel Merk grade 60 (0.040–0.063 mm). All reactions were carried out under argon atmosphere.

*tert-Butyl 3-(2-ethoxy-2-oxoethylidene)azetidine-1-carboxylate (* **6** *)*

To the suspension of NaH (60% dispersion in mineral oil, 1.88 g, 0.047 mol, 1.15 equiv.) in THF (150 mL) at 0 °C, triethyl phosphonoacetate (11 g, 0.054 mol, 1.2 equiv.) was added. The resulting mixture was allowed to warm to room temperature and stirred for 30 min. Then, it was cooled back to 0 °C, at which point a solution of tert-butyl 3-oxoazetidine-1-carboxylate (**5**) (7 g, 0.041 mol, 1 equiv.) in THF (50 mL) was added. The reaction mixture was allowed to reach room temperature and stirred for 18 h. Then, the mixture was diluted with ethyl acetate (100 mL) and washed with sat. aq. NaHCO_3_, water, and brine. The organic phase was separated, dried over anhydrous Na_2_SO_4_, filtered, and concentrated in vacuo. The residue was purified by column chromatography on silica gel eluting with 0 → 10% ethyl acetate in hexane to give the title compound as a clear oil. Yield 9 g (92%), colorless oil. The spectrum of the product matched that reported in the literature [27]. ^1^H NMR (300 MHz, CDCl_3_) δ 5.74 (dd, J = 4.5, 2.2 Hz, 1H), 4.80 (dd, J = 6.3, 2.9 Hz, 2H), 4.57 (dt, J = 5.3, 2.7 Hz, 2H), 4.31–4.02 (m, 2H), 1.45 (s, 8H), 1.26 (t, J = 7.1 Hz, 4H); LCMS (ESI): *m*/*z* (M + H) calcd, 242.3; found, 242.2.

*tert-Butyl 7-oxo-5-oxa-2-azaspiro[3.4]octane-2-carboxylate (* **4** *)*

To a suspension of NaH (60% dispersion in mineral oil, 4.2 g, 0.105 mol) in dry ether (150 mL), methyl glycolate (8.1 mL, 105 mmol) was added dropwise under argon. The resulting mixture was stirred at room temperature for 30 min, whereafter it was concentrated in vacuo. Dry DMSO (200 mL) was added, and the solution was cooled to 0 °C. A solution of **6** (21 g, 87.2 mmol) in dry DMSO (20 mL) was added. The reaction mixture was allowed to reach room temperature and stirred for 18 h. It was then diluted with 5% HCl (50 mL) and ether (200 mL). The organic phase was separated, dried over anhydrous Na_2_SO_4_, filtered, and concentrated in vacuo to give a yellowish oil. The latter was dissolved in a mixture of DMSO (300 mL) and water (30 mL) containing NaCl (10.2 g, 175 mmol). The resulting mixture was heated at 120 °C for 2 h under argon. Upon cooling to room temperature, the mixture was diluted with brine (100 mL) and ether (100 mL). The organic phase was separated, and the aqueous phase was extracted with ether (2 × 100 mL). The combined organic phases were washed with water (200 mL), dried over anhydrous Na_2_SO_4_, filtered, and concentrated in vacuo to give a yellowish oil. The latter was purified by column chromatography on silica gel eluting with 1:4.5 ethyl acetate—hexane to give the title compound as a yellowish waxy solid. Yield 12 g (61%). The spectrum of the product matched that reported in the literature [25]. ^1^H NMR (300 MHz, DMSO-*d*_6_) δ 4.06–3.81 (m, 6H), 2.78 (s, 2H), 1.36 (s, 9H); LCMS (ESI): *m*/*z* (M + H) calcd, 228.3; found, 228.2.

#### 3.1.1. General Procedure 1 for the Synthesis of Compounds **7a**–**r**

Ketone **4** (2.24 g, 0.01 mol, 1 equiv.) was dissolved in dimethylformamide dimethyl acetal (13 mL, 10 equiv.) and heated under reflux for 18 h. The volatiles were removed on a rotary evaporator, and the residue was co-evaporated again with toluene. The residue was dissolved in methanol (10 mL) and then was added dropwise at 0 °C to a solution of corresponding amidine hydrochloride (0.015 mol, 1.5 equiv) and sodium methoxide (0.9 g, 0.017 mol, 1.7 equiv.) in methanol (30 mL). The resulting mixture was heated at reflux for 8 h. The solvent was removed in vacuo and the residue was partitioned between 5% aqueous citric acid (50 mL) and dichloromethane (100 mL). The organic phase was separated, washed with water, dried over anhydrous Na_2_SO_4_, filtered, and concentrated in vacuo. The residue was purified by column chromatography on silica gel eluting with 50% → 100% ethyl acetate in hexane. Compounds **7a**–**d** were synthesized according to this procedure previously, and the spectra were reported [24].

*tert-butyl 2′-(4-fluorophenyl)-1H,7′H-spiro[azetidine-3,5′-furo[3,4-d]pyrimidine]-1-carboxylate (* **7e** *).*

The compound was synthesized according to General Procedure 1. Yield 1.75 g (49%), white solid, m.p. 132-133 °C, ^1^H NMR (300 MHz, DMSO-*d*_6_) δ 9.12 (s, 1H), 8.45 (dd, J = 8.8, 5.7 Hz, 2H), 7.36 (t, J = 8.8 Hz, 2H), 5.09 (s, 2H), 4.29 (d, J = 10.0 Hz, 2H), 4.15 (d, J = 9.7 Hz, 2H), 1.43 (s, 9H); ^13^C NMR (75 MHz, DMSO-*d*_6_) δ 170.72, 163.20, 162.84, 156.01, 151.65, 133.69, 130.77, 130.65, 130.30, 116.31, 116.02, 81.28, 79.55, 71.31, 63.57, 28.46. HRMS (ESI) *m*/*z* calcd for C_19_H_21_FN_3_O_3_ (M+H^+^) 358.1566, found 358.1568.

*tert-butyl 2′-(4-chlorophenyl)-1H,7′H-spiro[azetidine-3,5′-furo[3,4-d]pyrimidine]-1-carboxylate (* **7f** *).*

The compound was synthesized according to General Procedure 1. Yield 2.06 g (55%), white solid, m.p. 138–139 °C. ^1^H NMR (300 MHz, DMSO-*d*_6_) δ 9.14 (s, 1H), 8.41 (d, J = 8.4 Hz, 2H), 7.60 (d, J = 8.6 Hz, 2H), 5.10 (s, 2H), 4.29 (d, J = 9.6 Hz, 2H), 4.15 (d, J = 9.8 Hz, 2H), 1.43 (s, 9H); ^13^C NMR (75 MHz, DMSO-*d*_6_) δ 170.78, 163.12, 156.01, 151.70, 136.40, 135.99, 130.65, 130.03, 129.32, 81.28, 79.56, 71.30, 28.46. HRMS (ESI) *m*/*z* calcd for C_19_H_21_ClN_3_O_3_ (M+H^+^) 374.1271, found 374.1274.

*tert-butyl 2′-(3-chlorophenyl)-1H,7′H-spiro[azetidine-3,5′-furo[3,4-d]pyrimidine]-1-carboxylate (* **7g** *).*

The compound was synthesized according to General Procedure 1. Yield 1.91 g (51%), white solid, m.p. 122-123 °C. ^1^H NMR (300 MHz, DMSO-*d*_6_) δ 9.17 (s, 1H), 8.40–8.31 (m, 2H), 7.68–7.53 (m, 2H), 5.11 (s, 2H), 4.30 (d, J = 9.7 Hz, 2H), 4.15 (d, J = 9.7 Hz, 2H), 1.43 (s, 9H); ^13^C NMR (75 MHz, DMSO-*d*_6_) δ 170.88, 162.67, 156.01, 151.75, 139.21, 134.11, 131.24, 131.03, 127.77, 126.82, 81.27, 79.57, 71.31, 63.54, 28.46. HRMS (ESI) *m*/*z* calcd for C_19_H_21_ClN_3_O_3_ (M+H^+^) 374.1271, found 374.1272.

*tert-butyl 2′-(4-isopropylphenyl)-1H,7′H-spiro[azetidine-3,5′-furo[3,4-d]pyrimidine]-1- carboxylate (* **7h** *).*

The compound was synthesized according to General Procedure 1. Yield 1.68 g (44%), white solid, m.p. 94–95 °C. ^1^H NMR (300 MHz, DMSO-*d*_6_) δ 9.11 (s, 1H), 8.33 (d, J = 7.8 Hz, 2H), 7.41 (d, J = 7.9 Hz, 2H), 5.08 (s, 2H), 4.29 (d, J = 9.6 Hz, 2H), 4.15 (d, J = 9.7 Hz, 2H), 2.97 (m, 1H), 1.43 (s, 9H), 1.25 (d, J = 7.0 Hz, 6H); ^13^C NMR (75 MHz, DMSO-*d*_6_) δ 170.53, 164.11, 156.00, 152.08, 151.61, 134.83, 130.04, 128.38, 127.18, 81.26, 79.55, 71.33, 33.76, 29.40, 28.44, 24.06. HRMS (ESI) *m*/*z* calcd for C_22_H_28_N_3_O_3_ (M+H^+^) 382.2130, found 382.2130.

*tert-butyl 2′-[(4-methoxyphenoxy)methyl]-1H,7′H-spiro[azetidine-3,5′-furo[3,4-d]pyrimidine] -1-carboxylate (* **7i** *).*

The compound was synthesized according to General Procedure 1. Yield 2.68 g (67%), white solid, m.p. 88–89 °C. ^1^H NMR (300 MHz, DMSO-*d*_6_) δ 9.07 (s, 1H), 6.92 (d, J = 9.2 Hz, 2H), 6.84 (d, J = 9.2 Hz, 2H), 5.22 (s, 2H), 5.04 (s, 2H), 4.26 (d, J = 9.6 Hz, 2H), 4.12 (d, J = 9.7 Hz, 2H), 3.68 (s, 3H), 1.42 (s, 9H). ^13^C NMR (75 MHz, DMSO-*d*_6_) δ 170.45, 166.19, 155.98, 153.91, 152.56, 151.47, 131.04, 115.85, 114.96, 81.14, 79.56, 71.18, 71.07, 55.70, 55.31, 28.42. HRMS (ESI) *m*/*z* calcd for C_21_H_26_N_3_O_5_ (M+H^+^) 400.1872, found 400.1872.

*tert-butyl 2′-pyridin-3-yl-1H,7′H-spiro[azetidine-3,5′-furo[3,4-d]pyrimidine]-1-carboxylate (* **7j** *).*

The compound was synthesized according to General Procedure 1. Yield 1.90 g (56%), white solid, m.p. 107–108 °C. ^1^H NMR (300 MHz, DMSO-*d*_6_) 9.51 (s, 1H), 9.19 (s, 1H), 8.77–8.63 (m, 2H), 7.58 (dd, J = 7.9, 4.8 Hz, 1H), 5.12 (s, 2H), 4.31 (d, J = 9.7 Hz, 2H), 4.16 (d, J = 9.7 Hz, 2H), 1.44 (s, 9H); ^13^C NMR (75 MHz, DMSO-*d*_6_) δ 170.88, 162.55, 156.01, 152.04, 151.81, 149.39, 135.62, 132.66, 131.06, 124.31, 81.28, 79.57, 71.32, 63.54, 28.46. HRMS (ESI) *m*/*z* calcd for C_18_H_21_N_4_O_3_ (M+H^+^) 341.1613, found 341.1617.

*tert-butyl 2′-(2-methoxyethyl)-1H,7′H-spiro[azetidine-3,5′-furo[3,4-d]pyrimidine]-1-carboxylate (* **7k** *).*

The compound was synthesized according to General Procedure 1. Yield 1.57 g (49%), white solid, m.p. 65–66 °C. ^1^H NMR (300 MHz, DMSO-*d*_6_) δ 8.96 (s, 1H), 4.99 (s, 2H), 4.23 (d, J = 9.8 Hz, 2H), 4.12 (d, J = 9.7 Hz, 2H), 3.81 (t, J = 6.5 Hz, 2H), 3.22 (s, 3H), 3.14 (t, J = 6.5 Hz, 2H), 1.42 (s, 9H); ^13^C NMR (75 MHz, DMSO-*d*_6_) δ 169.96, 168.79, 156.00, 151.06, 129.74, 81.19, 79.51, 71.16, 70.62, 63.59, 58.21, 39.14, 28.44. HRMS (ESI) *m*/*z* calcd for C_16_H_24_N_3_O_4_ (M+H^+^) 322.1766, found 322.1765.

*tert-butyl 2′-(4-bromophenyl)-1H,7′H-spiro[azetidine-3,5′-furo[3,4-d]pyrimidine]-1-carboxylate (* **7l** *).*

The compound was synthesized according to General Procedure 1. Yield 2.13 g (51%), white solid, m.p. 139-140 °C. ^1^H NMR (300 MHz, DMSO-*d*_6_) δ 9.14 (s, 1H), 8.33 (d, J = 8.4 Hz, 2H), 7.74 (d, J = 8.4 Hz, 2H), 5.09 (s, 2H), 4.29 (d, J = 9.6 Hz, 2H), 4.15 (d, J = 9.6 Hz, 2H), 1.43 (s, 9H); ^13^C NMR (75 MHz, DMSO-*d*_6_) δ 170.78, 163.14, 155.98, 151.78, 136.28, 132.29, 130.69, 130.23, 125.38, 81.25, 79.56, 71.30, 63.70, 28.44. HRMS (ESI) *m*/*z* calcd for C_19_H_21_BrN_3_O_3_ (M+H^+^) 418.0766, found 418.0768.

*tert-butyl 2′-(3-bromophenyl)-1H,7′H-spiro[azetidine-3,5′-furo[3,4-d]pyrimidine]-1-carboxylate (* **7m** *).*

The compound was synthesized according to General Procedure 1. Yield 0.54 g (13%), white solid, m.p. 128–129 °C. ^1^H NMR (300 MHz, DMSO-*d*_6_) δ 9.16 (s, 1H), 8.54–8.51 (m, 1H), 8.39 (d, J = 7.9, 1H), 7.75 (dd, J = 8.0 Hz, 1H), 7.52 (t, J = 7.9 Hz, 1H), 5.10 (s, 2H), 4.30 (d, J = 9.6 Hz, 2H), 4.16 (d, J = 9.6 Hz, 2H), 1.43 (s, 9H); ^13^C NMR (75 MHz, DMSO-*d*_6_) δ 170.88, 162.56, 156.00, 151.75, 139.40, 134.13, 131.50, 131.02, 130.70, 127.19, 122.58, 81.27, 79.56, 71.31, 63.55, 28.46. HRMS (ESI) *m*/*z* calcd for C_19_H_21_BrN_3_O_3_ (M+H+) 418.0766, found 418.0767.

*tert-butyl 2′-pyridin-2-yl-1H,7′H-spiro[azetidine-3,5′-furo[3,4-d]pyrimidine]-1-carboxylate (* **7n** *).*

The compound was synthesized according to General Procedure 1. Yield 2.07 g (61%), white solid, m.p. 107–108 °C. ^1^H NMR (300 MHz, DMSO-*d*_6_) δ 9.20 (s, 1H), 8.75 (d, J = 2.7 Hz, 1H), 8.38 (d, J = 7.9 Hz, 1H), 8.03–7.94 (m, 1H), 7.59–7.51 (m, 1H), 5.11 (s, 2H), 4.32 (d, J = 9.6 Hz, 2H), 4.16 (d, J = 9.7 Hz, 2H), 1.43 (s, 9H); ^13^C NMR (75 MHz, DMSO-*d*_6_) δ 170.79, 163.82, 156.03, 154.59, 151.57, 150.13, 137.55, 131.34, 125.59, 124.01, 81.24, 79.56, 71.33, 63.56, 28.45. HRMS (ESI) *m*/*z* calcd for C_18_H_21_N_4_O_3_ (M+H^+^) 341.1613, found 341.1613.

*tert-butyl 2′-pyridin-4-yl-1H,7′H-spiro[azetidine-3,5′-furo[3,4-d]pyrimidine]-1-carboxylate (* **7o** *).*

The compound was synthesized according to General Procedure 1. Yield 2.04 g (60%), white solid, m.p. 100–101 °C. ^1^H NMR (300 MHz, DMSO-*d*_6_) δ 9.24 (s, 1H), 8.78 (d, J = 5.5 Hz, 2H), 8.26 (d, J = 4.4 Hz, 2H), 5.13 (s, 2H), 4.31 (d, J = 9.7 Hz, 2H), 4.16 (d, J = 9.7 Hz, 2H), 1.43 (s, 9H); ^13^C NMR (75 MHz, DMSO-*d*_6_) δ 171.09, 162.28, 156.00, 151.90, 151.01, 144.18, 131.99, 122.01, 81.27, 79.58, 71.30, 63.52, 40.79, 40.51, 40.23, 39.96, 39.68, 39.40, 39.12, 28.45. HRMS (ESI) *m*/*z* calcd for C_18_H_21_N_4_O_3_ (M+H^+^) 341.1613, found 341.1615.

*tert-butyl 2′-pyrazin-2-yl-1H,7′H-spiro[azetidine-3,5′-furo[3,4-d]pyrimidine]-1-carboxylate (* **7p** *).*

The compound was synthesized according to General Procedure 1. Yield 1.80 g (53%), white solid, m.p. 114–115 °C. ^1^H NMR (300 MHz, DMSO-*d*_6_) δ 9.53 (s, 1H), 9.25 (s, 1H), 8.82 (d, J = 7.0 Hz, 2H), 5.14 (s, 2H), 4.33 (d, J = 9.6 Hz, 2H), 4.18 (d, J = 9.7 Hz, 2H), 1.44 (s, 9H); ^13^C NMR (75 MHz, DMSO-*d*_6_) δ 171.10, 162.17, 156.01, 151.85, 149.91, 146.34, 145.08, 132.05, 81.26, 79.59, 71.32, 63.52, 28.46. HRMS (ESI) *m*/*z* calcd for C_17_H_20_N_5_O_3_ (M+H^+^) 342.1566, found 342.1566.

*tert-butyl 2′-ethyl-1H,7′H-spiro[azetidine-3,5′-furo[3,4-d]pyrimidine]-1-carboxylate (* **7q** *).*

Prepared according to General Procedure 1. Yield 1.39 g (48%), white solid, m.p. 64–65 °C. ^1^H NMR (300 MHz, DMSO-*d*_6_) δ 8.95 (d, J = 1.7 Hz, 1H), 4.99 (s, 1H), 4.23 (d, J = 9.6 Hz, 2H), 4.12 (d, J = 9.6 Hz, 2H), 2.93 (q, J = 7.4 Hz, 1H), 1.42 (s, 9H), 1.28 (t, J = 7.6 Hz, 3H); ^13^C NMR (75 MHz, DMSO-*d*_6_) δ 171.89, 169.94, 155.99, 151.13, 129.45, 81.14, 79.52, 71.18, 63.58, 32.15, 28.42, 12.99. HRMS (ESI) *m*/*z* calcd for C_15_H_22_N_3_O_3_ (M+H^+^) 292.1661, found 292.1664.

*tert-butyl 2′-isopropyl-1H,7′H-spiro[azetidine-3,5′-furo[3,4-d]pyrimidine]-1-carboxylate (* **7r** *).*

The compound was synthesized according to General Procedure 1. Yield 2.22 g (73%), white solid, m.p. 75–76 °C. ^1^H NMR (300 MHz, DMSO-*d*_6_) δ 8.97 (d, J = 1.0 Hz, 1H), 5.00 (s, 1H), 4.23 (d, J = 9.6 Hz, 2H), 4.10 (d, J = 9.7 Hz, 2H), 3.18 (dt, J = 14.2, 6.9 Hz, 0H), 1.42 (s, 9H), 1.27 (dd, J = 6.9, 1.1 Hz, 6H); ^13^C NMR (75 MHz, DMSO-*d*_6_) δ 175.12, 169.93, 155.99, 151.15, 129.54, 81.17, 79.52, 71.23, 63.60, 37.28, 28.43, 22.13. HRMS (ESI) *m*/*z* calcd for C_16_H_24_N_3_O_3_ (M+H^+^) 306.1817, found 306.1818.

#### 3.1.2. General Procedure 2 for the Synthesis of Compounds **3a**–**r**

Solution A. To a solution of 5-nitrofuranoic acid (75 mg, 0.47 mmol) in DMF (3 mL), CDI (97 mg, 0.6 mmol) was added at 0 °C, and the mixture was stirred at 0 °C for 1 h.

Solution B. To a solution of compound **7** (0.6 mmol) in dichloromethane (5 mL), trifluoroacetic acid (1 mL) was added dropwise at 0 °C, and the resulting mixture was stirred for 1 h. The volatiles were removed in vacuo (bath temperature < 30 °C) and the residue was dissolved in DMF (3 mL). Triethylamine (0.19 g, 1.9 mmol) was added; the mixture was stirred for 30 min and added dropwise to solution A.

The reaction mixture was stirred at room temperature for 18 h, poured into water (25 mL), and the mixture was extracted with ethyl acetate (3 × 20 mL). The combined organic extracts were washed with brine, dried over anhydrous Na_2_SO_4_, filtered, and concentrated in vacuo. The residue was purified by column chromatography on silica gel eluting with 10:1 dichloromethane-methanol. Compounds **3a**–**d** were synthesized according to this procedure previously, and the spectra were reported [24].

*2′-(4-fluorophenyl)-1-(5-nitro-2-furoyl)-7′H-spiro[azetidine-3,5′-furo[3,4-d]pyrimidine] (* **3e** *).*

Yield 44 mg (24%), white solid, m.p. 125-126 °C. ^1^H NMR (300 MHz, CDCl_3_) δ 8.90 (s, 1H), 8.55–8.42 (m, 2H), 7.43–7.32 (m, 2H), 7.19 (t, J = 8.7 Hz, 2H), 5.18 (s, 2H), 5.10 (d, J = 11.0 Hz, 1H), 4.99 (d, J = 11.0 Hz, 1H), 4.67 (d, J = 11.6 Hz, 1H), 4.56 (d, J = 11.6 Hz, 1H); ^13^C NMR (75 MHz, CDCl_3_) δ 170.06, 164.96 (d, J = 251.6 Hz), 164.83, 156.32, 150.02, 147.97, 132.96 (d, J = 3.2 Hz), 130.62 (d, J = 8.8 Hz), 129.06, 123.36, 117.26, 115.63 (d, J = 21.7 Hz), 111.66, 81.70, 72.01, 66.91, 63.35. HRMS (ESI) *m*/*z* calcd for C_19_H_14_FN_4_O_5_ (M+H^+^) 397.0948, found 397.0948.

*2′-(4-chlorophenyl)-1-(5-nitro-2-furoyl)-7′H-spiro[azetidine-3,5′-furo[3,4-d]pyrimidine] (* **3f** *).*

Yield 38 mg (20%), white solid, m.p. 129-128 °C. ^1^H NMR (300 MHz, CDCl_3_) δ 8.91 (s, 1H), 8.43 (d, J = 8.3 Hz, 2H), 7.49 (d, J = 8.3 Hz, 2H), 7.37 (d, J = 9.2 Hz, 2H), 5.19 (s, 2H), 5.13–4.97 (m, 2H), 4.72–4.51 (m, 2H); ^13^C NMR (75 MHz, CDCl_3_) δ 170.11, 164.80, 156.31, 150.04, 147.96, 137.57, 135.23, 129.76, 129.39, 128.88, 117.25, 111.65, 81.70, 72.00, 66.89, 63.33. HRMS (ESI) *m*/*z* calcd for C_19_H_14_ClN_4_O_5_ (M+H^+^) 413.0652, found 413.0654.

*2′-(3-chlorophenyl)-1-(5-nitro-2-furoyl)-7′H-spiro[azetidine-3,5′-furo[3,4-d]pyrimidine] (* **3g** *).*

Yield 66 mg (34%), white solid, m.p. 124–126 °C. ^1^H NMR (300 MHz, CDCl_3_) δ 8.91 (s, 1H), 8.47 (s, 1H), 8.35 (d, J = 7.5 Hz, 1H), 7.54–7.32 (m, 4H), 5.18 (s, 2H), 5.09 (d, J = 11.0 Hz, 1H), 4.99 (d, J = 11.0 Hz, 1H), 4.66 (d, J = 11.4 Hz, 1H), 4.55 (d, J = 11.6 Hz, 1H); ^13^C NMR (75 MHz, CDCl_3_) δ 170.18, 164.46, 156.31, 150.11, 147.93, 138.53, 134.84, 131.21, 129.93, 129.75, 128.53, 126.51, 117.34, 111.76, 81.68, 72.03, 66.95, 63.39. HRMS (ESI) *m*/*z* calcd for C_19_H_14_ClN_4_O_5_ (M+H^+^) 413.0652, found 413.0652.

*2′-(4-isopropylphenyl)-1-(5-nitro-2-furoyl)-7′H-spiro[azetidine-3,5′-furo[3,4-d]pyrimidine] (* **3h** *).*

Yield 117 mg (59%), white solid, m.p. 116-117 °C. ^1^H NMR (300 MHz, CDCl_3_) δ 8.90 (s, 1H), 8.38 (d, J = 8.6 Hz, 2H), 7.43 -7.34 (m, 4H), 5.19 (s, 2H), 5.10 (d, J = 11.0 Hz, 1H), 5.00 (d, J = 11.0 Hz, 1H), 4.67 (d, J = 11.5 Hz, 1H), 4.56 (d, J = 11.6 Hz, 1H), 3.01 (p, J = 7.0 Hz, 1H), 1.32 (d, J = 6.9 Hz, 6H); ^13^C NMR (75 MHz, CDCl_3_) δ 169.90, 165.91, 156.31, 152.51, 149.96, 148.02, 134.46, 128.74, 128.53, 126.77, 117.22, 111.66, 81.73, 72.06, 66.95, 63.38, 34.07, 23.71. HRMS (ESI) *m*/*z* calcd for C_22_H_21_N_4_O_5_ (M+H^+^) 421.1511, found 421.1512.

*2′-[(4-methoxyphenoxy)methyl]-1-(5-nitro-2-furoyl)-7′H-spiro[azetidine-3,5′-furo[3,4-d]pyrimidine] (* **3i** *).*

Yield 119 mg (58%), white solid, m.p. 118-119 °C. ^1^H NMR (300 MHz, CDCl_3_) δ 8.90 (s, 1H), 7.42–7.30 (m, 2H), 6.96 (d, J = 8.8 Hz, 2H), 6.83 (d, J = 8.9 Hz, 2H), 5.32 (s, 2H), 5.16 (s, 2H), 5.07 (d, J = 11.0 Hz, 1H), 4.96 (d, J = 11.0 Hz, 1H), 4.64 (d, J = 11.6 Hz, 1H), 4.53 (d, J = 11.6 Hz, 1H), 3.77 (s, 3H); ^13^C NMR (75 MHz, CDCl_3_) δ 170.33, 167.43, 156.29, 154.30, 152.33, 150.13, 147.89, 130.46, 117.28, 115.93, 114.60, 111.66, 81.58, 71.96, 71.31, 66.83, 63.28, 55.59. HRMS (ESI) *m*/*z* calcd for C_21_H_19_N_4_O_7_ (M+H^+^) 439.1253, found 439.1253.

*1-(5-nitro-2-furoyl)-2′-pyridin-3-yl-7′H-spiro[azetidine-3,5′-furo[3,4-d]pyrimidine] (* **3j** *).*

Yield 61 mg (34%), white solid, m.p. 118-119 °C. ^1^H NMR (300 MHz, DMSO-*d*_6_) δ 9.50 (d, J = 2.0 Hz, 1H), 9.27 (s, 1H), 8.72 (dd, J = 4.8, 1.7 Hz, 1H), 8.67 (dt, J = 8.0, 2.0 Hz, 1H), 7.82–7.68 (m, 1H), 7.57 (dd, J = 8.0, 4.8 Hz, 1H), 7.49–7.35 (m, 1H), 5.16 (s, 2H), 5.01 (d, J = 10.6 Hz, 1H), 4.86 (d, J = 10.6 Hz, 1H), 4.59 (d, J = 11.4 Hz, 1H), 4.37 (d, J = 11.5 Hz, 1H); ^13^C NMR (75 MHz, DMSO-*d*_6_)170.92, 162.61, 156.84, 152.25, 151.97, 151.87, 149.33, 147.84, 135.73, 132.67, 130.64, 124.34, 123.07, 117.55, 113.52, 82.10, 71.58, 66.60, 63.21. HRMS (ESI) *m*/*z* calcd for C_18_H_14_N_5_O_5_ (M+H^+^) 380.0994, found 380.0996.

*2′-(2-methoxyethyl)-1-(5-nitro-2-furoyl)-7′H-spiro[azetidine-3,5′-furo[3,4-d]pyrimidine] (* **3k** *).*

Yield 66 mg (39%), white solid, m.p. 92-93 °C. ^1^H NMR (300 MHz, CDCl_3_) δ 8.81 (s, 1H), 7.37 (d, J = 3.8 Hz, 1H), 7.33 (d, J = 3.8 Hz, 1H), 5.11 (s, 2H), 5.04 (d, J = 10.9 Hz, 1H), 4.94 (d, J = 11.0 Hz, 1H), 4.62 (d, J = 11.6 Hz, 1H), 4.51 (d, J = 11.8 Hz, 1H), 3.92 (t, J = 6.4 Hz, 2H), 3.37 (s, 3H), 3.30 (t, J = 6.4 Hz, 2H); ^13^C NMR (75 MHz, CDCl_3_) δ 169.95, 156.27, 149.75, 147.93, 129.10, 117.28, 111.73, 109.53, 81.63, 72.00, 70.72, 66.91, 63.31, 58.70, 39.41. HRMS (ESI) *m*/*z* calcd for C_16_H_17_N_4_O_6_ (M+H^+^) 361.1148, found 361.1148.

*2′-(4-bromophenyl)-1-(5-nitro-2-furoyl)-7′H-spiro[azetidine-3,5′-furo[3,4-d]pyrimidine] (* **3l** *).*

Yield 110 mg (51%), white solid, m.p. 140-141 °C. ^1^H NMR (300 MHz, DMSO-*d*_6_) δ 9.25 (s, 1H), 8.35 (d, J = 8.6 Hz, 2H), 7.80 (d, J = 3.9 Hz, 1H), 7.75 (d, J = 8.6 Hz, 2H), 7.40 (d, J = 3.9 Hz, 1H), 5.16 (s, 2H), 5.01 (d, J = 10.6 Hz, 1H), 4.87 (d, J = 10.6 Hz, 1H), 4.60 (d, J = 11.5 Hz, 1H), 4.38 (d, J = 11.5 Hz, 1H); ^13^C NMR (75 MHz, DMSO-*d*_6_) δ 170.85, 163.32, 156.86, 152.16, 147.83, 136.34, 132.28, 130.29, 125.39, 117.55, 113.54, 82.11, 71.55, 63.22. HRMS (ESI) *m*/*z* calcd for C_19_H_14_BrN_4_O_5_ (M+H^+^) 457.0147, found 457.0149.

*2′-(3-bromophenyl)-1-(5-nitro-2-furoyl)-7′H-spiro[azetidine-3,5′-furo[3,4-d]pyrimidine] (* **3m** *).*

Yield 52 mg (24%), white solid, m.p. 136-137 °C. ^1^H NMR (300 MHz, DMSO-*d*_6_) δ 9.27 (s, 1H), 8.53 (s, 1H), 8.41 (d, J = 7.8 Hz, 1H), 7.84-7.72 (m, 2H), 7.52 (t, J = 8.0 Hz, 1H), 7.41 (s, 1H), 5.17 (s, 2H), 5.07-4.97 (m, 1H), 4.94–4.82 (m, 1H), 4.60 (d, J = 11.1 Hz, 1H), 4.39 (d, J = 11.4 Hz, 1H); ^13^C NMR (75 MHz, DMSO-*d*_6_) δ 170.93, 162.66, 156.86, 152.20, 147.84, 139.39, 134.17, 131.51, 130.73, 130.59, 127.23, 122.58, 117.55, 113.54, 82.10, 71.56, 66.63, 63.21. HRMS (ESI) *m*/*z* calcd for C_19_H_14_BrN_4_O_5_ (M+H^+^) 457.0147, found 457.0147

*1-(5-nitro-2-furoyl)-2′-pyridin-2-yl-7′H-spiro[azetidine-3,5′-furo[3,4-d]pyrimidine] (* **3n** *).*

Yield 91 mg (51%), white solid, m.p. 117-118 °C. ^1^H NMR (300 MHz, CDCl_3_) δ 9.01 (s, 1H), 8.86 (d, J = 4.1 Hz, 1H), 8.55 (d, J = 8.0 Hz, 1H), 7.94–7.84 (m, 1H), 7.49–7.41 (m, 1H), 7.34 (m, J = 13.3, 3.8 Hz, 2H), 5.23 (s, 2H), 5.04 (dd, J = 30.9, 10.7 Hz, 2H), 4.60 (dd, J = 32.7, 11.2 Hz, 2H); ^13^C NMR (75 MHz, CDCl_3_) δ 170.82, 164.13, 156.39, 153.51, 150.42, 149.87, 147.99, 137.67, 130.94, 125.56, 124.14, 117.35, 111.74, 81.71, 72.19, 66.91, 63.40. HRMS (ESI) *m*/*z* calcd for C_18_H_14_N_5_O_5_ (M+H^+^) 380.0994, found 380.0994.

*1-(5-nitro-2-furoyl)-2′-pyridin-4-yl-7′H-spiro[azetidine-3,5′-furo[3,4-d]pyrimidine] (* **3o** *).*

Yield 62 mg (35%), white solid, m.p. 115-116 °C. ^1^H NMR (300 MHz, CDCl_3_) δ 8.94 (s, 1H), 8.71 (d, J = 5.0 Hz, 2H), 8.24 (d, J = 5.1 Hz, 2H), 7.37–7.23 (m, 2H), 5.13 (s, 2H), 4.97 (d, J = 14.7 Hz, 2H), 4.53 (d, J = 15.6 Hz, 2H); ^13^C NMR (75 MHz, CDCl_3_) δ 170.44, 163.55, 150.35, 147.83, 144.04, 130.94, 123.76, 122.07, 117.31, 111.81, 81.63, 71.91, 66.80, 63.25. HRMS (ESI) *m*/*z* calcd for C_18_H_14_N_5_O_5_ (M+H^+^) 380.0994, found 380.0999.

*1-(5-nitro-2-furoyl)-2′-pyrazin-2-yl-7′H-spiro[azetidine-3,5′-furo[3,4-d]pyrimidine] (* **3p** *).*

Yield 107 mg (60%), white solid, m.p. 122–123 °C. ^1^H NMR (300 MHz, DMSO-*d*_6_) δ 9.55 (s, 1H), 9.36 (s, 1H), 8.88–8.78 (m, 2H), 7.80 (d, J = 3.8 Hz, 1H), 7.40 (d, J = 3.9 Hz, 1H), 5.20 (s, 2H), 5.04 (d, J = 10.5 Hz, 1H), 4.89 (d, J = 10.7 Hz, 1H), 4.62 (d, J = 11.3 Hz, 1H), 4.40 (d, J = 11.5 Hz, 1H); ^13^C NMR (75 MHz, DMSO-*d*_6_) δ 171.22, 162.33, 156.94, 152.38, 151.95, 149.98, 147.93, 146.45, 145.18, 145.16, 131.72, 117.62, 113.60, 82.17, 71.66, 66.67, 63.28. HRMS (ESI) *m*/*z* calcd for C_17_H_13_N_5_O_5_ (M+H^+^) 381.0947, found 381.0946.

*2′-ethyl-1-(5-nitro-2-furoyl)-7′H-spiro[azetidine-3,5′-furo[3,4-d]pyrimidine] (* **3q** *).*

Yield 62 mg (40%), white solid, m.p. 96-97 °C. ^1^H NMR (300 MHz, CDCl_3_) δ 8.80 (s, 1H), 7.39 (d, J = 3.8 Hz, 1H), 7.34 (d, J = 3.8 Hz, 1H), 5.12 (s, 2H), 5.06 (d, J = 10.9 Hz, 1H), 4.95 (d, J = 11.0 Hz, 1H), 4.63 (d, J = 11.6 Hz, 1H), 4.52 (d, J = 11.5 Hz, 1H), 3.06 (q, J = 7.6 Hz, 2H), 1.40 (t, J = 7.6 Hz, 3H); ^13^C NMR (75 MHz, CDCl_3_) δ 173.51, 169.68, 156.28, 149.76, 147.98, 128.67, 117.22, 111.65, 81.65, 71.97, 66.91, 32.51, 12.67. HRMS (ESI) *m*/*z* calcd for C_15_H_15_N_4_O_5_ (M+H^+^) 331.1042, found 331.1043.

*2′-(4-isopropylphenyl)-1-(5-nitro-2-furoyl)-7′H-spiro[azetidine-3,5′-furo[3,4-d]pyrimidine] (* **3r** *).*

Yield 90 mg (56%), white solid, m.p. 114–115 °C. ^1^H NMR (300 MHz, CDCl_3_) ^1^H NMR (300 MHz, CDCl_3_) δ 8.80 (s, 1H), 7.38 (d, J = 3.8 Hz, 1H), 7.34 (d, J = 3.8 Hz, 1H), 5.12 (s, 2H), 5.05 (d, J = 11.0 Hz, 1H), 4.95 (d, J = 10.9 Hz, 1H), 4.63 (d, J = 11.9 Hz, 1H), 4.52 (d, J = 11.6 Hz, 1H), 3.30 (hept, J = 6.9 Hz, 1H), 1.37 (d, J = 6.9 Hz, 6H); ^13^C NMR (75 MHz, CDCl_3_) δ 176.77, 169.63, 156.27, 149.68, 147.99, 128.68, 117.20, 111.65, 81.64, 72.00, 66.94, 63.30, 37.59, 21.66. HRMS (ESI) *m*/*z* calcd for C_16_H_17_N_4_O_5_ (M+H^+^) 345.1198, found 345.1198.

*1-(5-nitro-2-furoyl)-2′-(4-pyrimidin-5-ylphenyl)-7′H-spiro[azetidine-3,5′-furo[3,4-d]pyrimidine] (* **3s** *).*

Pyrimidine-5-boronic acid (0.034 g, 0.273 mmol), compound **3l** (0.1 g, 0.219 mmol), [(C_5_H_4_P(C_6_H_5_)_2_)_2_)Fe]PdCl_2_·CH_2_Cl_2_ (0.016 g, 0.022 mmol) and Cs_2_CO_3_ (0.143 g, 0.438 mmol) were dissolved in a 100 mL screw cap vial under argon atmosphere in degassed dioxane/H_2_O 10:1 (10 mL). The mixture was heated for 6 h at 105 °C, H_2_O (50 mL) was added, and the mixture was extracted with EtOAc (2 × 50 mL). The combined organic layers were dried (Na_2_SO_4_), then evaporated and dried to constant mass on evaporating rotor. The residue was purified by column chromatography (silica gel, CH_2_Cl_2_ with 2.5% MeOH). Yield 50 mg (50%), white solid, m.p. 136–137 °C. ^1^H NMR (300 MHz, DMSO-*d*_6_) δ 9.28 (s, 1H), 9.24 (d, J = 3.2 Hz, 3H), 8.57 (d, J = 8.3 Hz, 2H), 8.02 (d, J = 8.4 Hz, 2H), 7.81 (d, J = 3.9 Hz, 1H), 7.41 (d, J = 3.9 Hz, 1H), 5.19 (s, 2H), 5.04 (d, J = 10.2 Hz, 1H), 4.89 (d, J = 11.1 Hz, 1H), 4.62 (d, J = 11.6 Hz, 1H), 4.40 (d, J = 11.2 Hz, 1H). ^13^C NMR (75 MHz, DMSO-*d*_6_) δ 170.85, 163.55, 158.04, 156.86, 155.25, 152.20, 151.89, 147.81, 137.44, 136.66, 132.88, 130.25, 129.09, 127.79, 117.58, 113.58, 82.12, 71.61, 66.65, 63.25. HRMS (ESI) *m*/*z* calcd for C_23_H_17_N_6_O_5_ (M+H^+^) 457.1260, found 457.1261.

*1-(5-nitro-2-furoyl)-2′-[4-(4,4,5,5-tetramethyl-1,3,2-dioxaborolan-2-yl)phenyl]-7′H-spiro[azetidine-3,5′-furo[3,4-d]pyrimidine] (* **3t** *).*

Bis(pinacolato)diboron (0.133 g, 0.525 mmol), compound **3l** (0.2 g, 0.437 mmol), [(C_5_H_4_P(C_6_H_5_)_2_)_2_)Fe]PdCl_2_·CH_2_Cl_2_ (0.032 g, 0.044 mmol) and AcOK (0.129 g, 1.31 mmol) were dissolved in a 100 mL screw cap vial under argon atmosphere in degassed dioxane (10 mL). The mixture was heated for 6 h at 105 °C, H_2_O (50 mL) was added, and the mixture was extracted with EtOAc (2 × 50 mL). The combined organic layers were dried (Na_2_SO_4_) and concentrated in vacuo. Residue was purified by column chromatography (silica gel, CH_2_Cl_2_ with 2.5% MeOH). Yield 49 mg (45%), white solid, m.p. 119–120 °C. ^1^H NMR (300 MHz, CDCl_3_) δ 8.94 (s, 1H), 8.47 (d, J = 8.1 Hz, 2H), 7.96 (d, J = 8.1 Hz, 2H), 7.43–7.32 (m, 2H), 5.20 (s, 2H), 5.10 (d, J = 11.0 Hz, 1H), 5.00 (d, J = 11.0 Hz, 1H), 4.67 (d, J = 11.6 Hz, 1H), 4.57 (d, J = 11.6 Hz, 1H), 1.39 (s, 12H); ^13^C NMR (75 MHz, CDCl_3_) δ 170.03, 165.69, 156.31, 150.01, 147.98, 139.06, 135.01, 129.34, 127.53, 117.22, 111.65, 83.96, 81.71, 72.03, 66.90, 63.35, 24.83. HRMS (ESI) *m*/*z* calcd for C_25_H_26_BN_4_O_7_ (M+H^+^) 505.1894, found 505.1895.

*2′-[4-(1-methyl-1H-pyrazol-4-yl)phenyl]-1-(5-nitro-2-furoyl)-7′H-spiro[azetidine-3,5′-furo[3,4-d]pyrimidine] (* **3u** *).*

1-methyl-1H-pyrazole-4-boronic acid pinacol ester (0.057 g, 0.273 mmol), compound **3l** (0.1 g, 0.219 mmol), [(C_5_H_4_P(C_6_H_5_)_2_)_2_)Fe]PdCl_2_·CH_2_Cl_2_ (0.016 g, 0.022 mmol), and Cs_2_CO_3_ (0.143 g, 0.438 mmol) were dissolved in a 100 mL screw cap vial under argon atmosphere in degassed dioxane/H_2_O 10:1 (10 mL). The mixture was heated for 6 h at 105 °C, H_2_O (50 mL) was added, and the mixture was extracted with EtOAc (2 × 50 mL). The combined organic layers were dried (Na_2_SO_4_) and concentrated in vacuo. Residue was purified by column chromatography (silica gel, CH_2_Cl_2_ with 2.5% MeOH). Yield 40 mg (40%), white solid, m.p. 138-139 °C. ^1^H NMR (300 MHz, DMSO-*d*_6_) δ 9.21 (s, 1H), 8.39 (d, J = 8.3 Hz, 3H), 8.25 (s, 1H), 7.96 (s, 1H), 7.80 (d, J = 3.9 Hz, 1H), 7.73 (d, J = 8.2 Hz, 3H), 7.40 (d, J = 3.9 Hz, 1H), 5.15 (s, 2H), 5.01 (d, J = 10.6 Hz, 1H), 4.87 (d, J = 10.7 Hz, 1H), 4.59 (d, J = 11.5 Hz, 1H), 4.38 (d, J = 11.5 Hz, 1H), 3.89 (s, 4H); ^13^C NMR (75 MHz, DMSO-*d*_6_) δ 170.60, 164.06, 156.87, 152.00, 151.89, 147.83, 136.78, 135.86, 134.56, 129.55, 128.93, 128.82, 125.49, 121.64, 117.55, 113.54, 82.12, 80.18, 71.58, 66.64, 64.38, 63.26. HRMS (ESI) *m*/*z* calcd for C_23_H_19_N_6_O_5_ (M+H^+^) 459.1416, found 459.1416.

### 3.2. Biological Activity Evaluation

Compounds **3a**–**u** were tested against the *Mycobacterium tuberculosis* H37Rv drug-sensitive strain relative to isoniazid that was served as a positive control. The measurements were performed in triplicate. The *M. tuberculosis* H37Rv strain (originated from the Institute of Hygiene and Epidemiology in Prague, 1976) was obtained on 7 August 2013 from the Federal Scientific Center for Expertise of Medical Products (RF Ministry of Health Care). The lyophilized strain was inoculated on Löwenstein–Jensen growth medium. The minimal inhibitory concentration (MIC) of the compounds was determined using a REMA (resazurin microtiter plate assay) [28]. The testing was performed as described previously [22].

### 3.3. In Silico Evaluation

#### 3.3.1. Protein Preparation

A protein model of deazaflavin-dependent nitroreductase [29] was preprocessed before calculations using the protein prepwizard tool from the Schrodinger suite. During preprocessing, errors such as missing amino acid sidechains, incorrect protonation states, missing hydrogens, incorrect bond orders, angles, etc. were fixed. Solvent molecules (water) were removed and restrained minimization (for XRAY structures) was performed [30]. The ligand geometry was generated by the LigPrep module. All molecular modeling operations were carried out in the OPLS4 force field [31]. Schrödinger Suite 2022-4 [32] was used for calculations.

#### 3.3.2. Induced Fit Docking of Molecules

The docking grid box was calculated based on reference ligand position and size (grid placement on complex ligand centroid, maximum grid side is 12 Å). In the case of ligand absence, it was calculated by residues involved in potential interactions (for Ddn, the centroid was between FMN and Tyr65/130/133/136). For each ligand, 20 poses were generated. The best-fitting pose was selected by a comparison with reference ligand nitrofuran moiety positioning in the protein active site (if present in the PDB files) or by orientation towards FMN and interacting amino acids. Ligands present in available PDB models were redocked for docking quality validation and scoring function reference values’ calculation.

The binding poses’ clustering was also used as an important parameter showing ligand binding quality. Additionally, the observed cluster should replicate the pharmacophore characteristics of the reference ligands (if present). This value is shown as stars in parentheses (see Table 2). Three stars—more than 60% clustered docking solutions with RMSD less than 1.5 Å; two stars—40–60% clustered docking solutions with RMSD less than 3 Å; and one star—less than 40% or solutions with no clustering.

## 4. Conclusions

A series of 18 new **7′**H-spiro[azetidine-3,5′-furo[3,4-d]pyrimidine]s **3a**–**r** were synthesized in four steps. Additionally, three more substances were obtained starting from compound **3l** by Pd-catalyzed cross-coupling to demonstrate the possibility of modifying the target compounds. Most of the compounds showed the same activity as isoniazid against the *Mycobacterium tuberculosis* H37Rv drug-sensitive strain. The compounds **3f** (2′-(4-chlorophenyl)-1-(5-nitro-2-furoyl)-7′H-spiro[azetidine-3,5′-furo[3,4-d]pyrimidine]) and **3l** (2′-(4-bromophenyl)-1-(5-nitro-2-furoyl)-7′H-spiro[azetidine-3,5’-furo[3,4-d] pyrimidine]) were the leads with MIC values lower than the positive control. Some SAR parameters were evaluated, but further optimization of the lead compounds is required. Evidence of the successful design of the new series is that the antimycobacterial activity of compounds **3f,l** was 400 times greater than that of the parent compounds **1** and **2** [22,23]. The in silico calculation results confirmed that the likely primary target of the new compounds is Ddn reductase of *M. tuberculosis*. In general, the novel spirocyclic scaffold shows excellent properties for anti-tuberculosis drug development.

## Figures and Tables

**Table 1:** Structure of substituents and yields of compounds **7a**–**r** and **3a**–**u**.

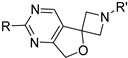 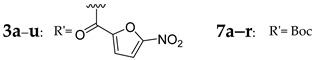																					
R	a	b	c	d	e	f	g	h	i	j	k	l	m	n	o	p	q	r	s	t	u
	*n*-Pr	Ph	Me	cyclo-Hex	4-FC_6_H_5_	4-ClC_6_H_5_	3-ClC_6_H_5_	4-iPrC_6_H_5_	(4-MeOC_6_H_4_)OCH_2_	3-Py	(CH_2_)_2_OMe	4-BrC_6_H_5_	3-BrC_6_H_5_	2-Py	4-Py	2′-pyrazine	Et	*i*-Pr	See Scheme 2	See Scheme 2	See Scheme 2
Yield of **7** (%)	74	44	57	84	49	55	51	44	67	56	49	51	13	61	60	53	48	73	-	-	-
Yield of **3** (%)	36	44	41	36	24	20	34	59	58	34	39	51	24	51	35	60	40	56	50	45	40

**Table 2:** Minimal inhibitory concentration (MIC, µg/mL) of tested compounds and isoniazid (positive control) against MTb, results of induced fit docking (GScore, kcal/mol) of the investigated compounds into the active cavity of Ddn, and calculated lipophilicity value. Stars indicate the quality of binding pose, where *** is good, ** is fair, and * is low. The MIC values are mean from three different assays (errors were in the range of ±5–10% of the reported values). The compounds with MIC values lower than those for positive control are highlighted by green. The LogD values were calculated using ACD/LogD 6.00 software.

	Compound 3																					Control
	a	b	c	d	e	f	g	h	i	j	k	l	m	n	o	p	q	r	s	t	u	
**MIC**, µg/mL *Mtb* (H37Rv)	0.031	0.031	6.2	0.050	0.031	0.004	0.016	0.125	0.031	1.6	3.1	0.008	0.031	1.6	1.6	6.2	0.8	0.062	0.062	0.8	1.6	0.005– 0.025
**IFD GScore**, (kcal/mol) and binding pose quality	−8.45 (**)	−8.61 (***)	−7.37 (**)	−8.24 (***)	−8.08 (***)	−8.80 (***)	−8.50 (***)	−7.90 (*)	−8.42 (**)	−7.88 (***)	−7.58 (***)	−9.05 (***)	−8.81 (***)	−7.85 (**)	−7.90 (*)	−7.72 (*)	−7.89 (*)	−8.03 (**)	−8.04 (**)	−8.57 (*)	−7.85 (*)	−6.68 (***)
**CLogD** at pH 7.2	1.79	3.09	0.73	2.79	3.31	3.86	3.89	4.43	2.31	2.02	0.74	4.03	4.06	2.01	1.83	1.42	1.26	1.61	2.39	2.89	_	_

## Data Availability

Data are contained within the Article and Supplementary Materials.

## Supplementary Materials

The following supporting information can be downloaded at https://www.mdpi.com/article/10.3390/molecules29133071/s1: Copies of ^1^H and ^13^C NMR spectra; molecular modeling data and visualization.
