# Supplementary material for: The Nitrofuran-Warhead-Equipped Spirocyclic Azetidines Show Excellent Activity against Mycobacterium tuberculosis
Source: Molecules. 2024 Jun 27;29(13):3071. doi: 10.3390/molecules29133071 (PMC11243650; doi:10.3390/molecules29133071)

The Nitrofuran-Warhead-Equipped Spirocyclic Azetidines Show Excellent Activity against *M. Tuberculosis*

Kristina Komarova [1], Lyubov Vinogradova [1], Alexey Lukin [1], Maxim Zhuravlev [1], Dmitry Deniskin [1], Mikhail Chudinov [1,*], Maxim Gureev [2], Marine Dogonadze [3], Natalia Zabolotnykh [3], Tatiana Vinogradova [3], Anastasia Lavrova [3,4] and Petr Yablonskiy [5]

[1] Lomonosov Institute of Fine Chemical Technologies, MIREA—Russian Technological University,
119454 Moscow, Russia; kristinka-komarova.1999@mail.ru (K.K.); vlv010599@yandex.ru (L.V.);
alex-look@yandex.ru (A.L.); max.2903@mail.ru (M.Z.); deniskin.02d@mail.ru (D.D.)
[2] Institute of Cytology, Russian Academy of Sciences, Tikhoretsky Ave 4, 194064 St-Petersburg, Russia; max_technik@mail.ru
[3] Saint-Petersburg State Research Institute of Phthisiopulmonology of the Ministry of Healthcare of the
Russian Federation, 191036 Saint Petersburg, Russia; marine-md@mail.ru (M.D.); vinogradova@spbniif.ru (T.V.); zabol-natal@yandex.ru (N.Z.); aurebours@googlemail.com (A.L.)
[4] Sophya Kovalevskaya North-West Mathematical Research Center, Immanuel Kant Baltic Federal University, 236041 Kaliningrad, Russia
[5] Department of Hospital Surgery, Faculty of Medicine, Saint Petersburg State University, 199034 Saint Petersburg, Russia; piotr_yablonskii@mail.ru
* Correspondence: chudinov@mirea.ru

NMR spectra of synthesized compounds

^{1}H NMR spectra for compound 6 3
^{1}H NMR spectra for compound 4 4
^{1}H and ^{13}C NMR spectra for compound 7e 5
^{1}H and ^{13}C NMR spectra for compound 7f 6
^{1}H and ^{13}C NMR spectra for compound 7g 7
^{1}H and ^{13}C NMR spectra for compound 7h 8
^{1}H and ^{13}C NMR spectra for compound 7i 9
^{1}H and ^{13}C NMR spectra for compound 7j 10
^{1}H and ^{13}C NMR spectra for compound 7k 11
^{1}H and ^{13}C NMR spectra for compound 7l 12
^{1}H and ^{13}C NMR spectra for compound 7m 13
^{1}H and ^{13}C NMR spectra for compound 7n 14
^{1}H and ^{13}C NMR spectra for compound 7o 15
^{1}H and ^{13}C NMR spectra for compound 7p 16
^{1}H and ^{13}C NMR spectra for compound 7q 17
^{1}H and ^{13}C NMR spectra for compound 7r 18

^{1}H and ^{13}C NMR spectra for compound 3a 19
^{1}H and ^{13}C NMR spectra for compound 3b 20
^{1}H and ^{13}C NMR spectra for compound 3c 21
^{1}H and ^{13}C NMR spectra for compound 3d 22
^{1}H and ^{13}C NMR spectra for compound 3e 23
^{1}H and ^{13}C NMR spectra for compound 3f 24
^{1}H and ^{13}C NMR spectra for compound 3g 25
^{1}H and ^{13}C NMR spectra for compound 3h 26
^{1}H and ^{13}C NMR spectra for compound 3i 27
^{1}H and ^{13}C NMR spectra for compound 3j 28
^{1}H and ^{13}C NMR spectra for compound 3k 29
^{1}H and ^{13}C NMR spectra for compound 3l 30
^{1}H and ^{13}C NMR spectra for compound 3m 31
^{1}H and ^{13}C NMR spectra for compound 3n 32
^{1}H and ^{13}C NMR spectra for compound 3o 33
^{1}H and ^{13}C NMR spectra for compound 3p 34
^{1}H and ^{13}C NMR spectra for compound 3q 35
^{1}H and ^{13}C NMR spectra for compound 3r 36
^{1}H and ^{13}C NMR spectra for compound 3u 37
^{1}H and ^{13}C NMR spectra for compound 3t 38
^{1}H and ^{13}C NMR spectra for compound 3s 39

^{1}H NMR spectra for compound 6

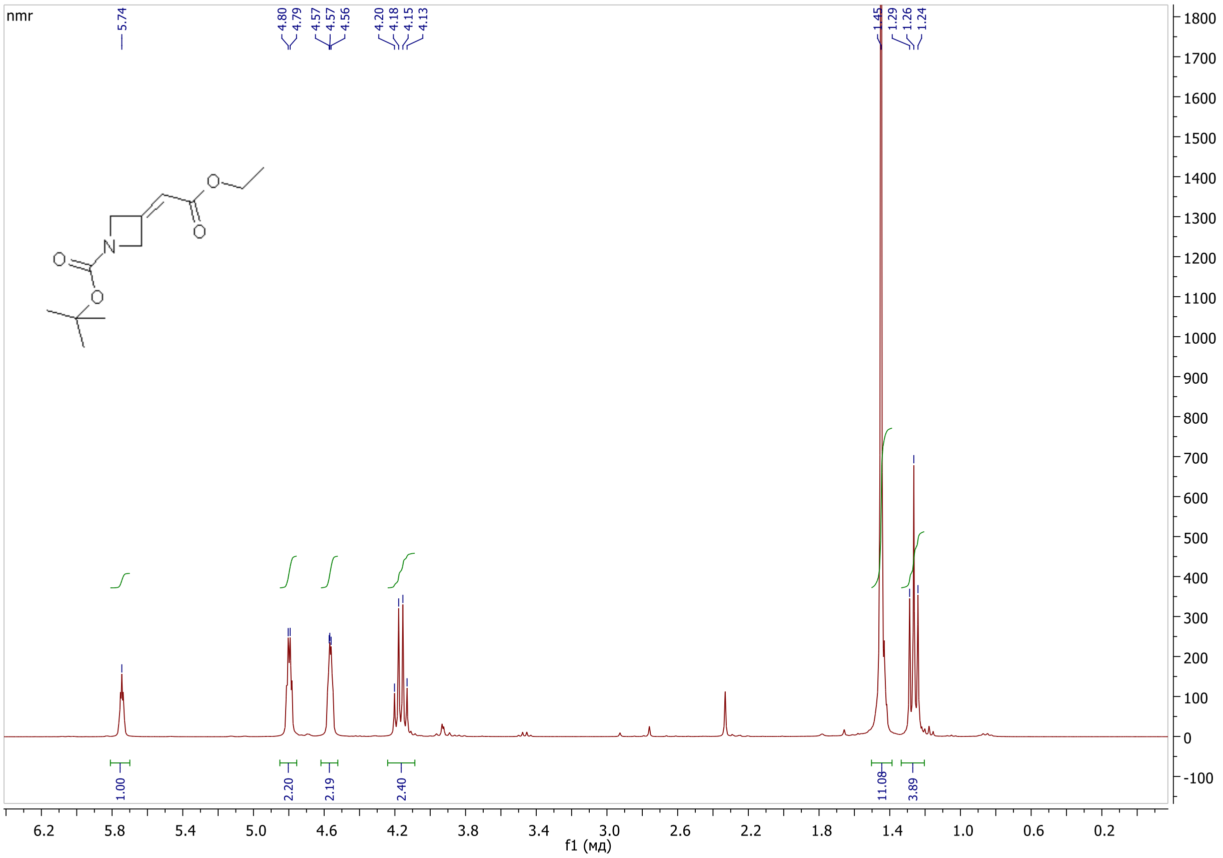

^{1}H NMR spectra for compound 4

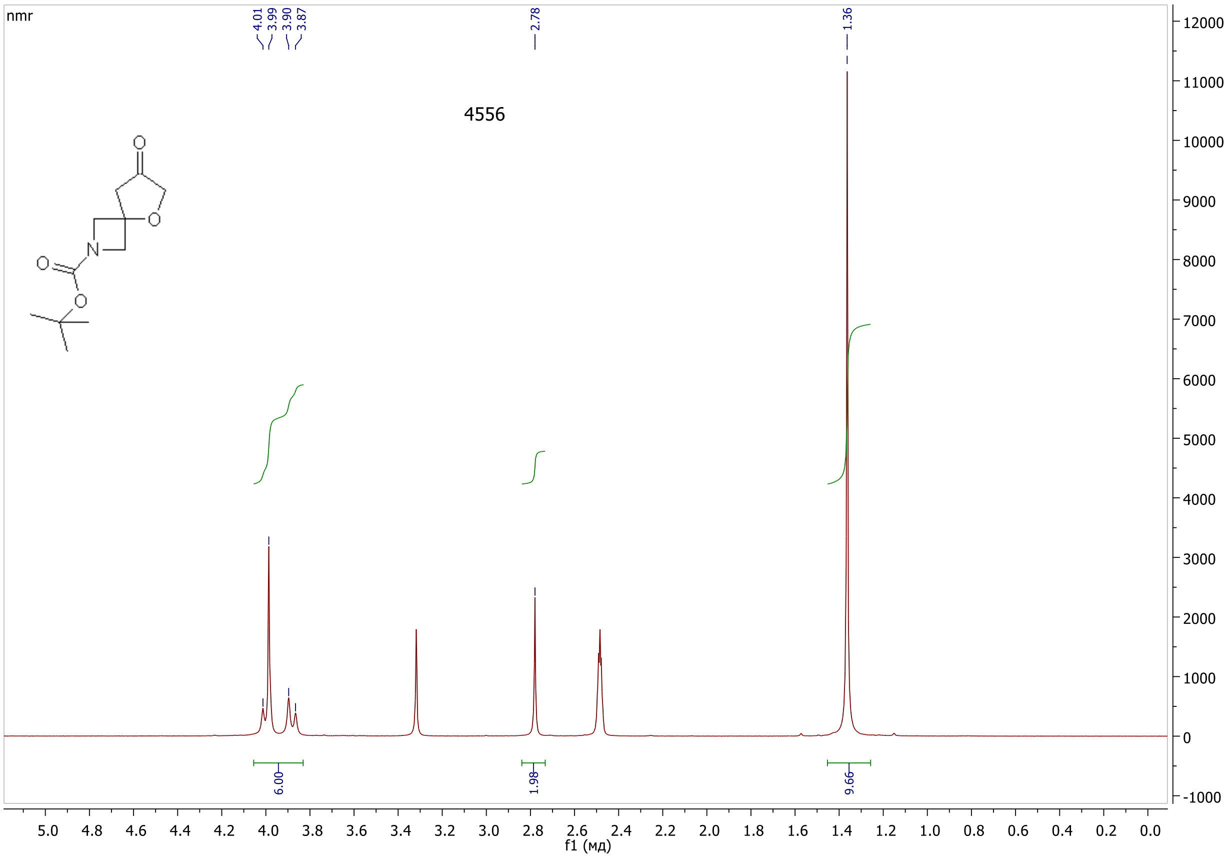

^{1}H and ^{13}C NMR spectra for compound 7e

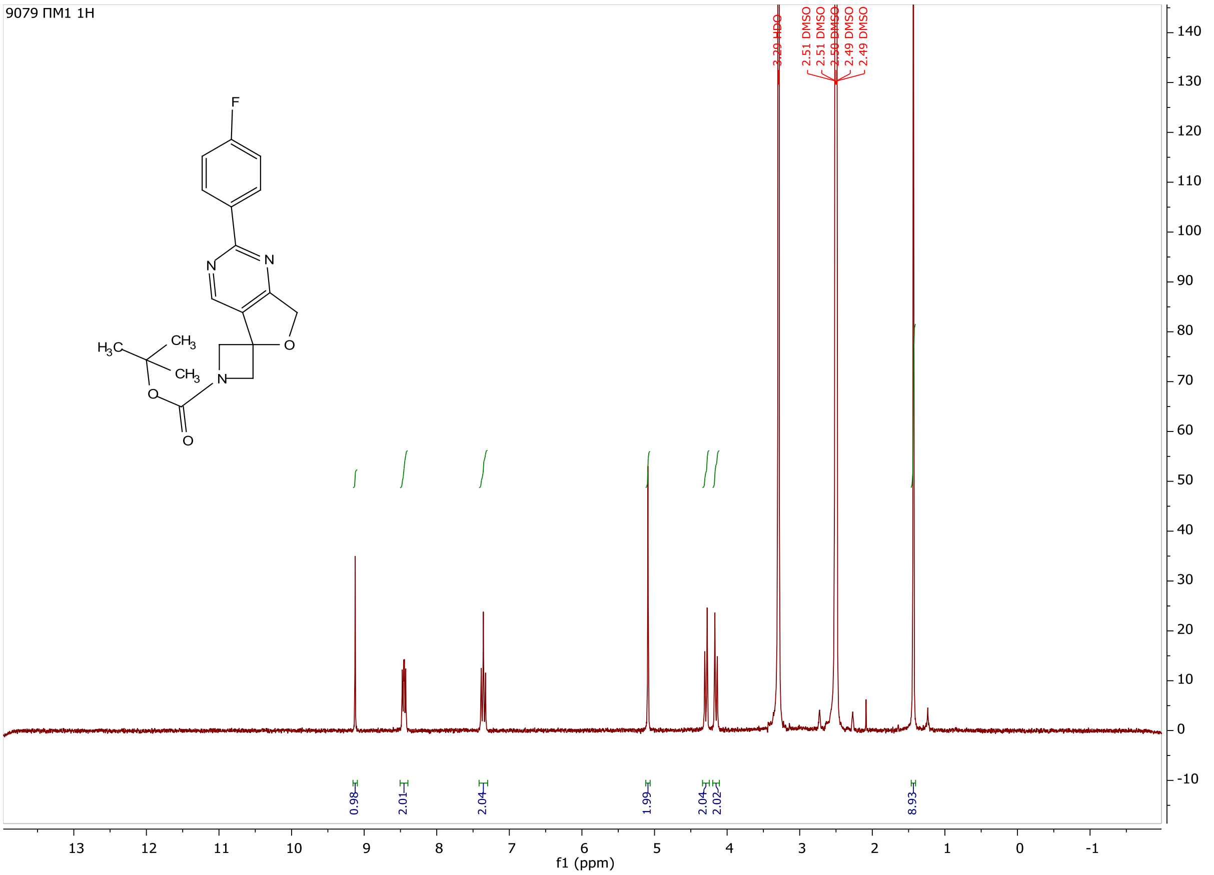

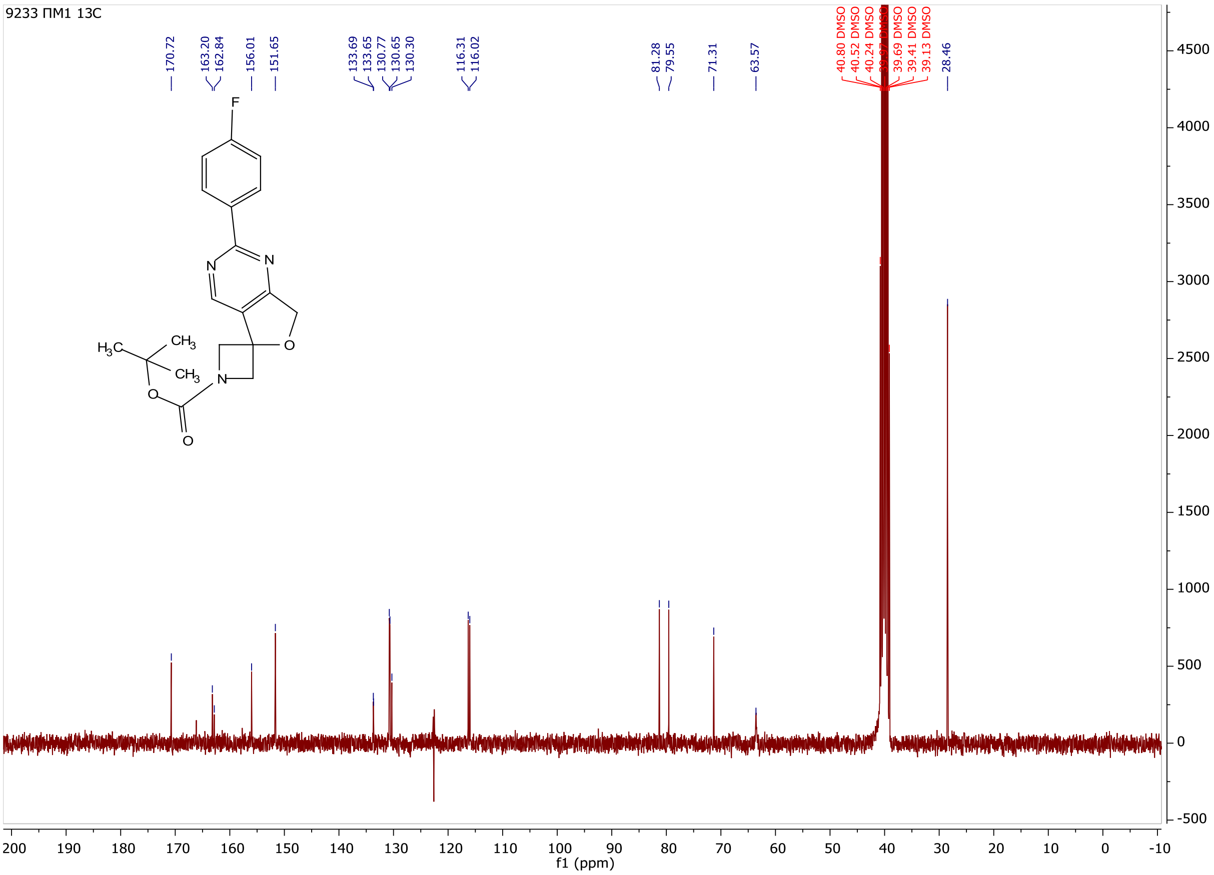

^{1}H and ^{13}C NMR spectra for compound 7f

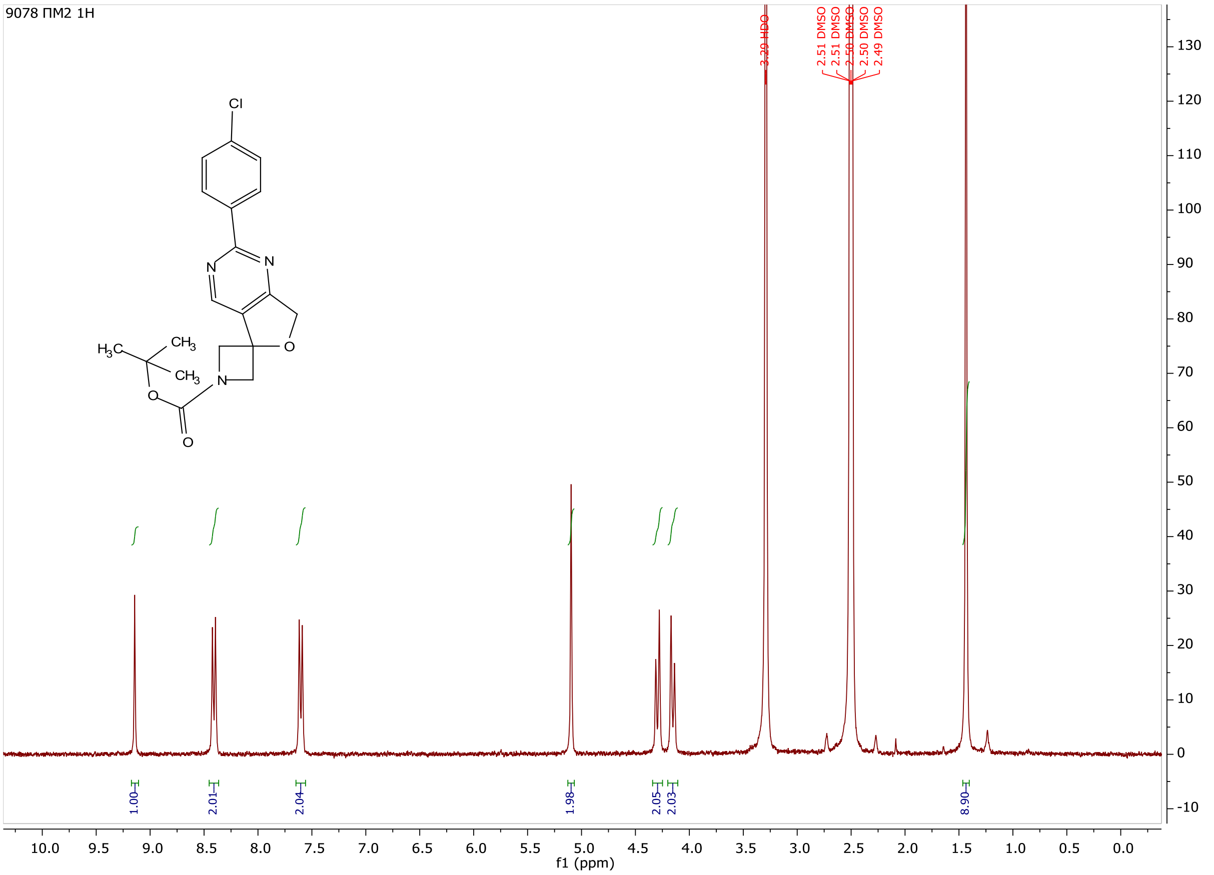

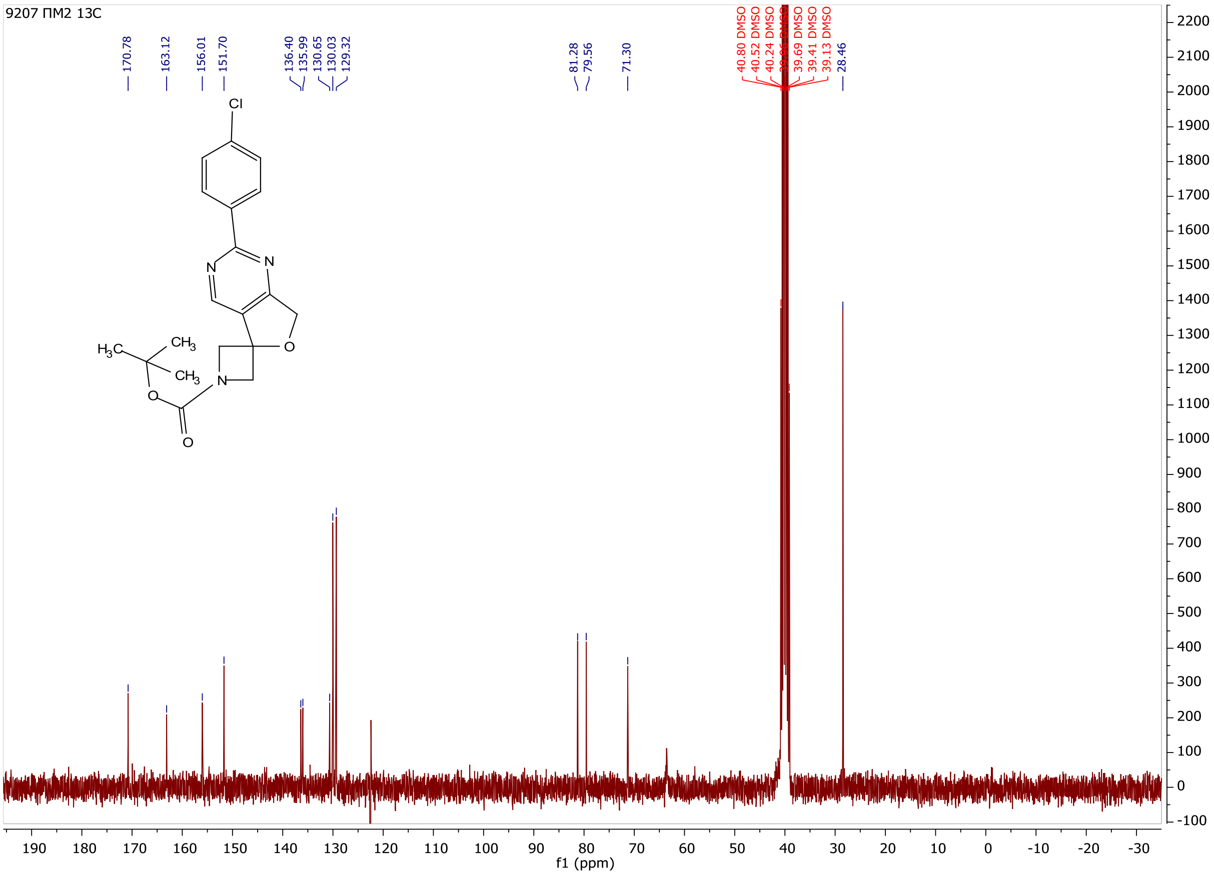

^{1}H and ^{13}C NMR spectra for compound 7g

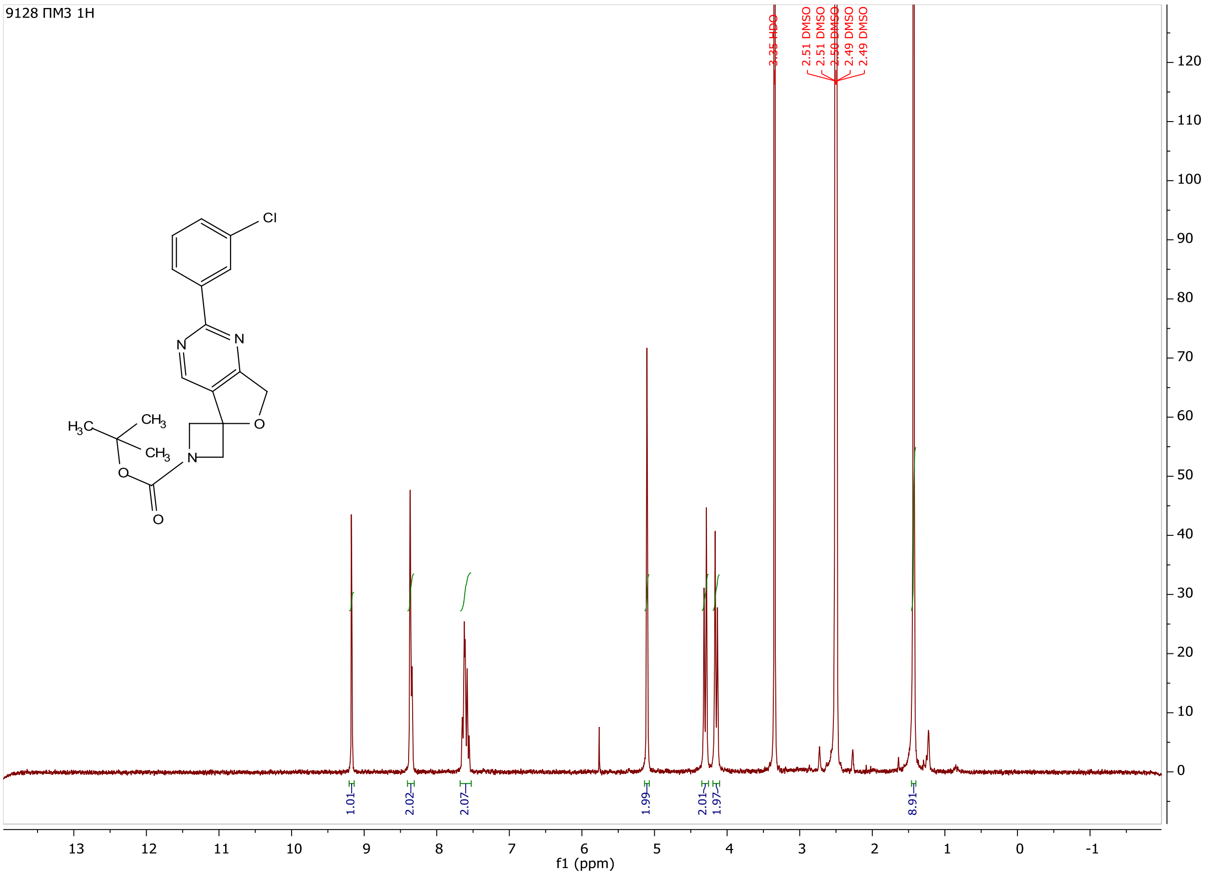

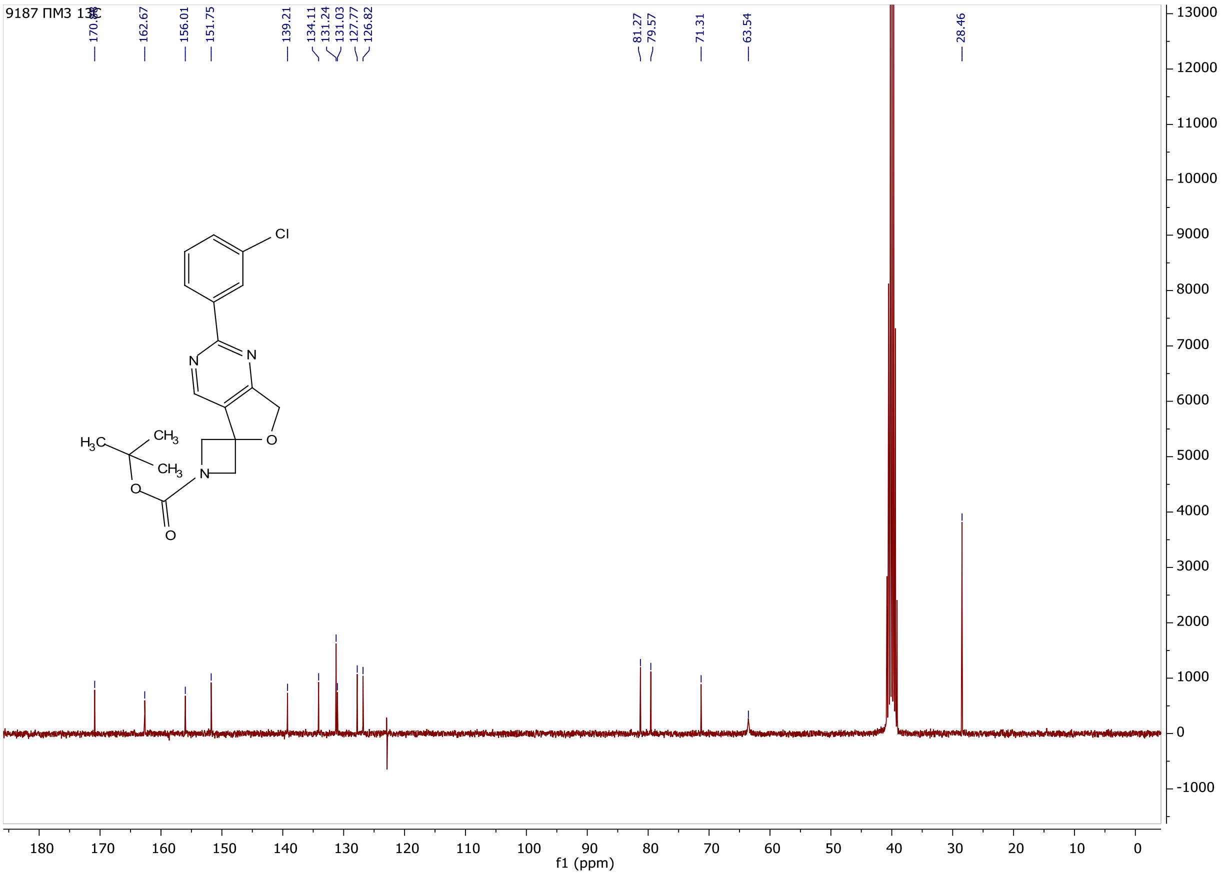

^{1}H and ^{13}C NMR spectra for compound 7h

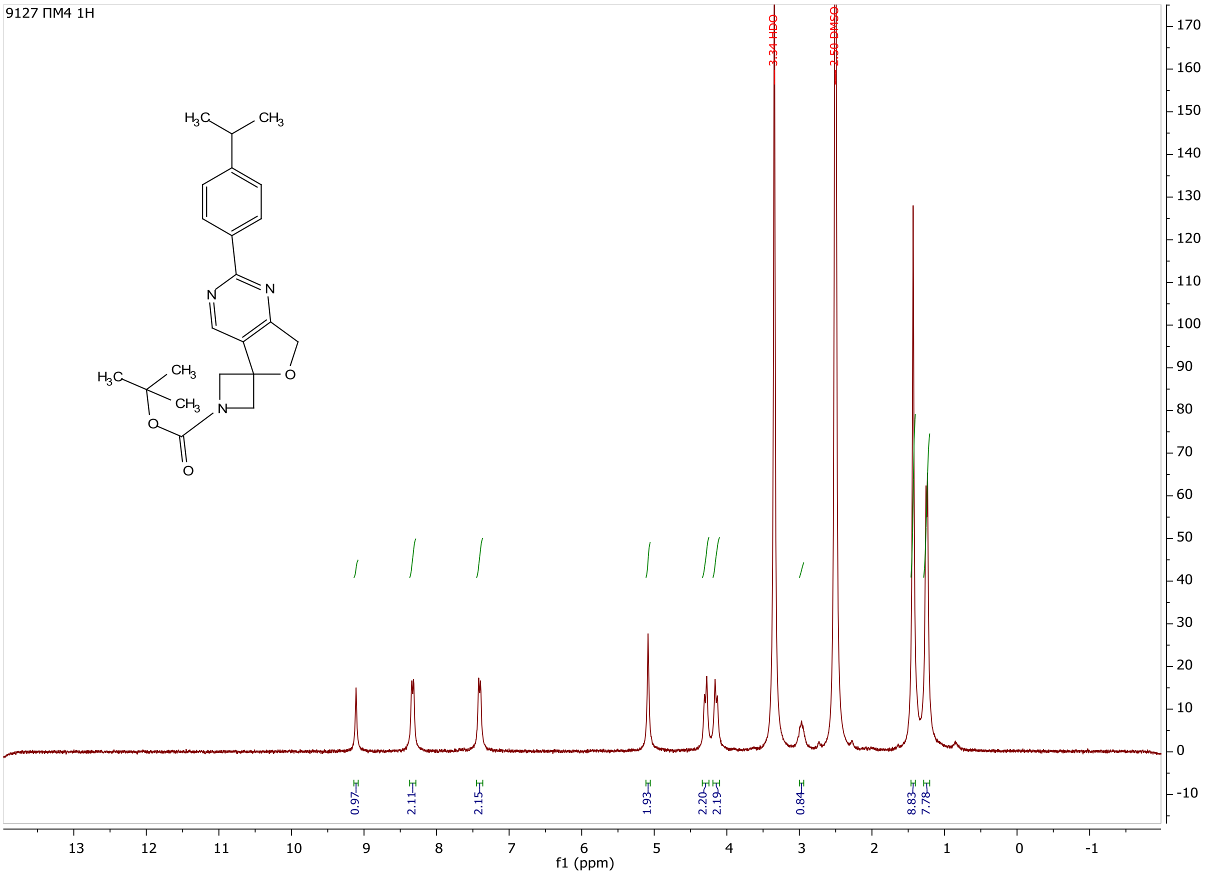

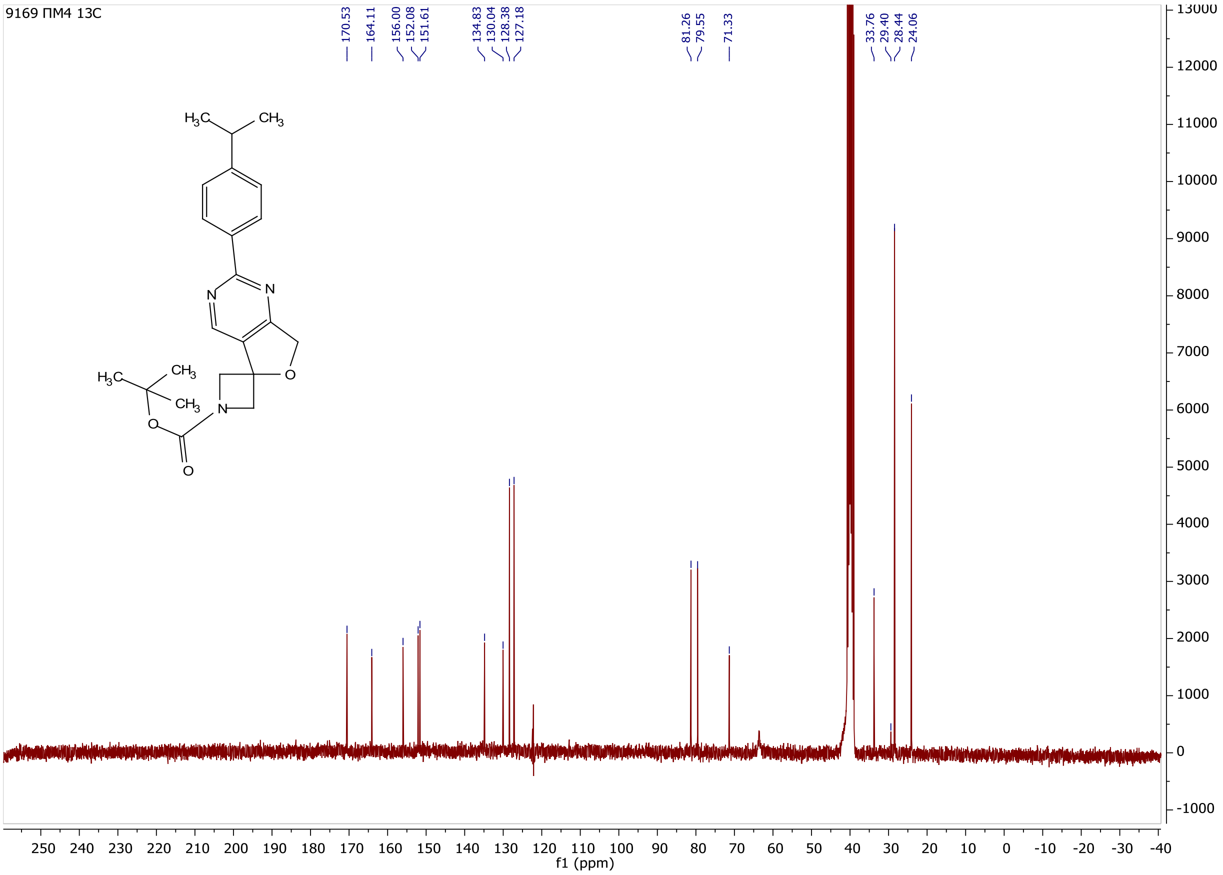

^{1}H and ^{13}C NMR spectra for compound 7i

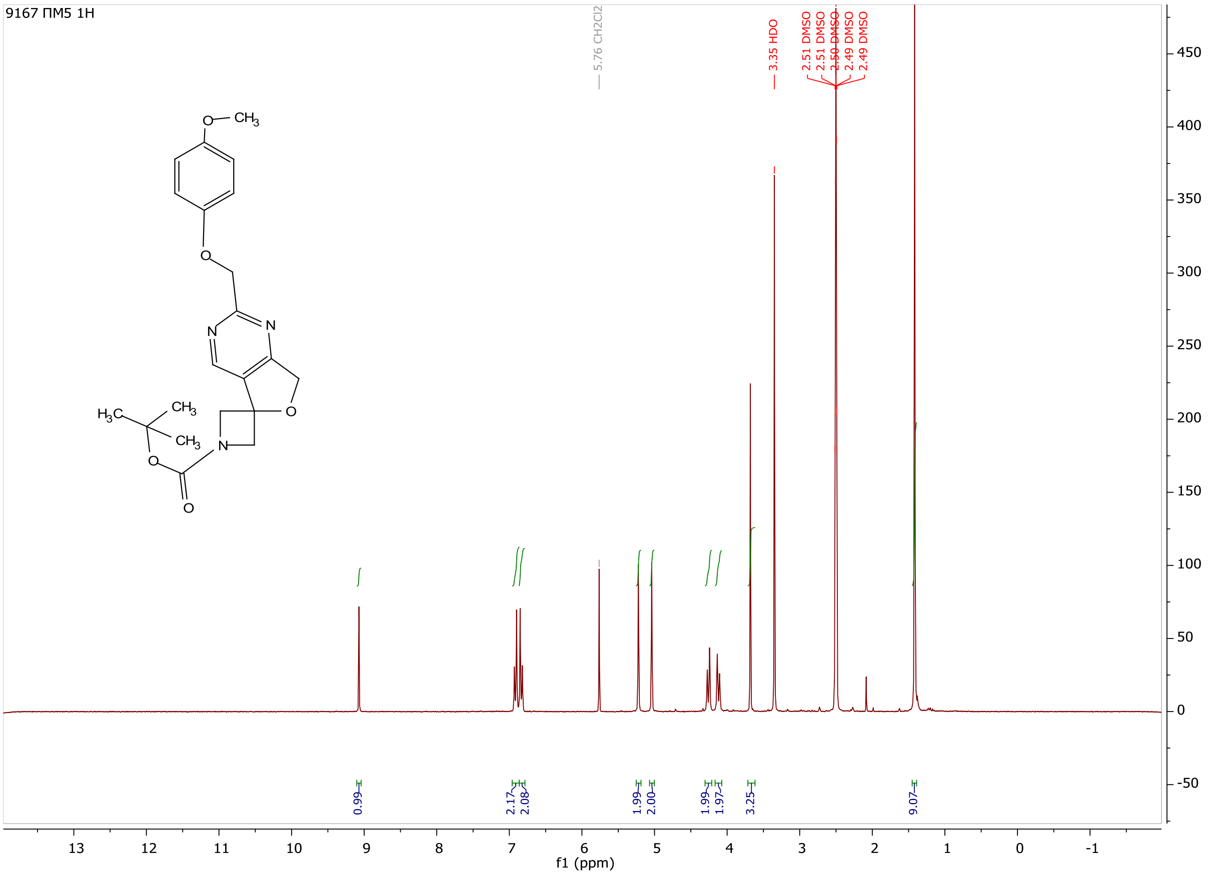

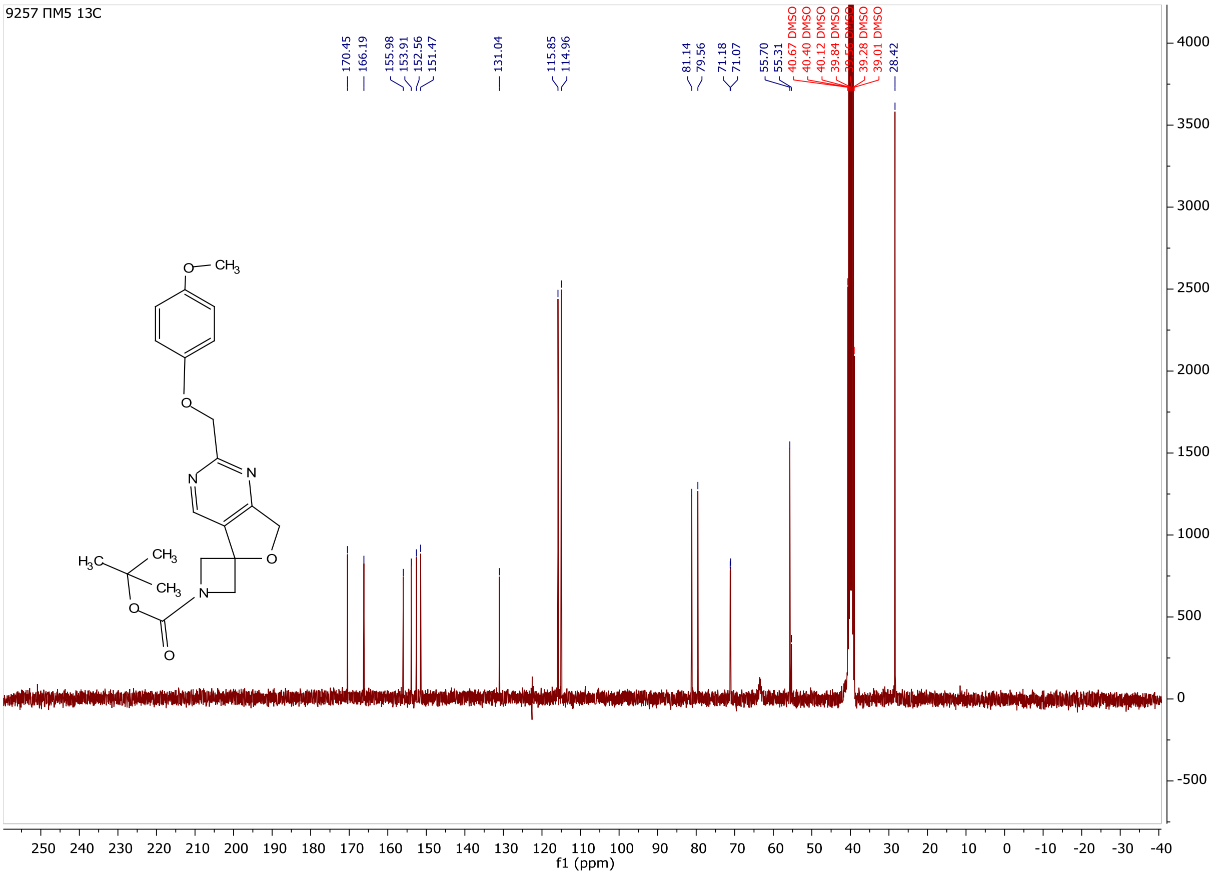

^{1}H and ^{13}C NMR spectra for compound 7j

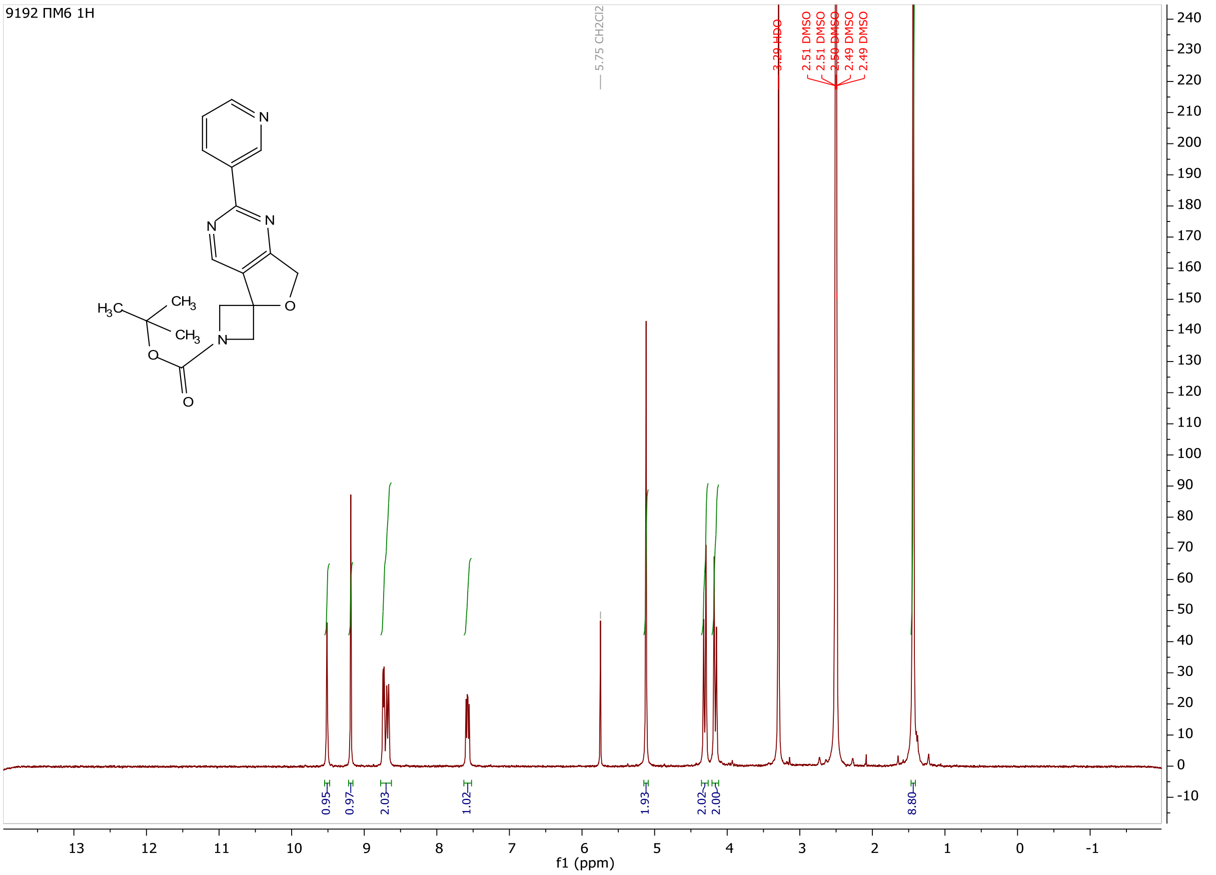

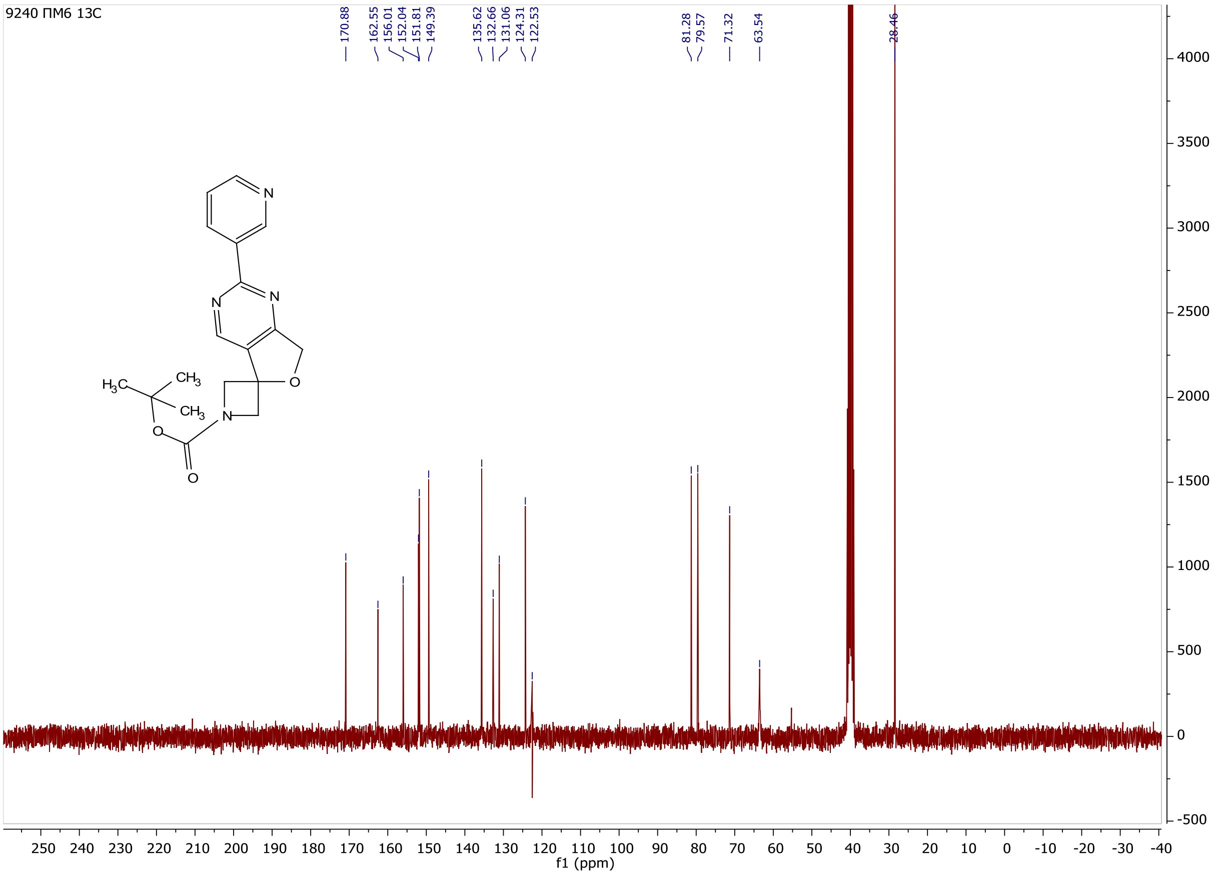

^{1}H and ^{13}C NMR spectra for compound 7k

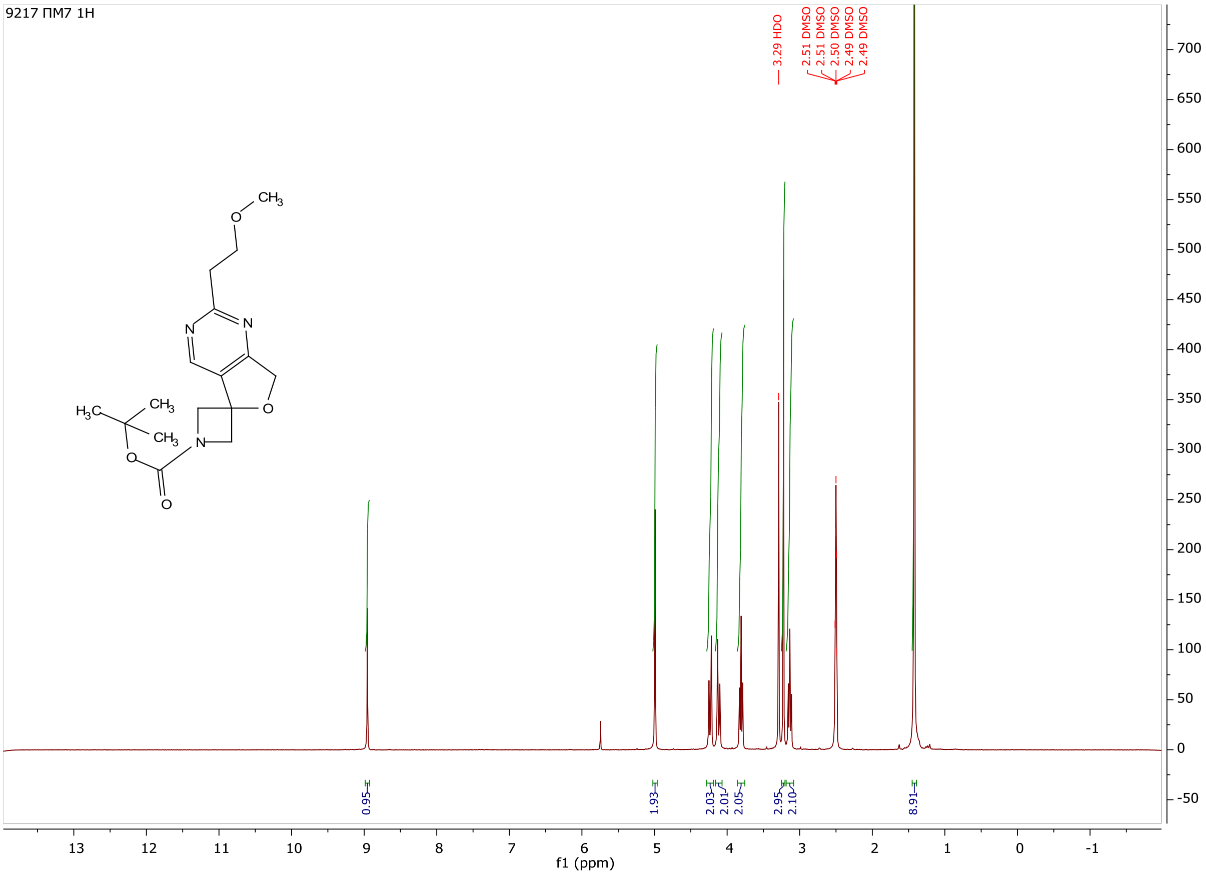

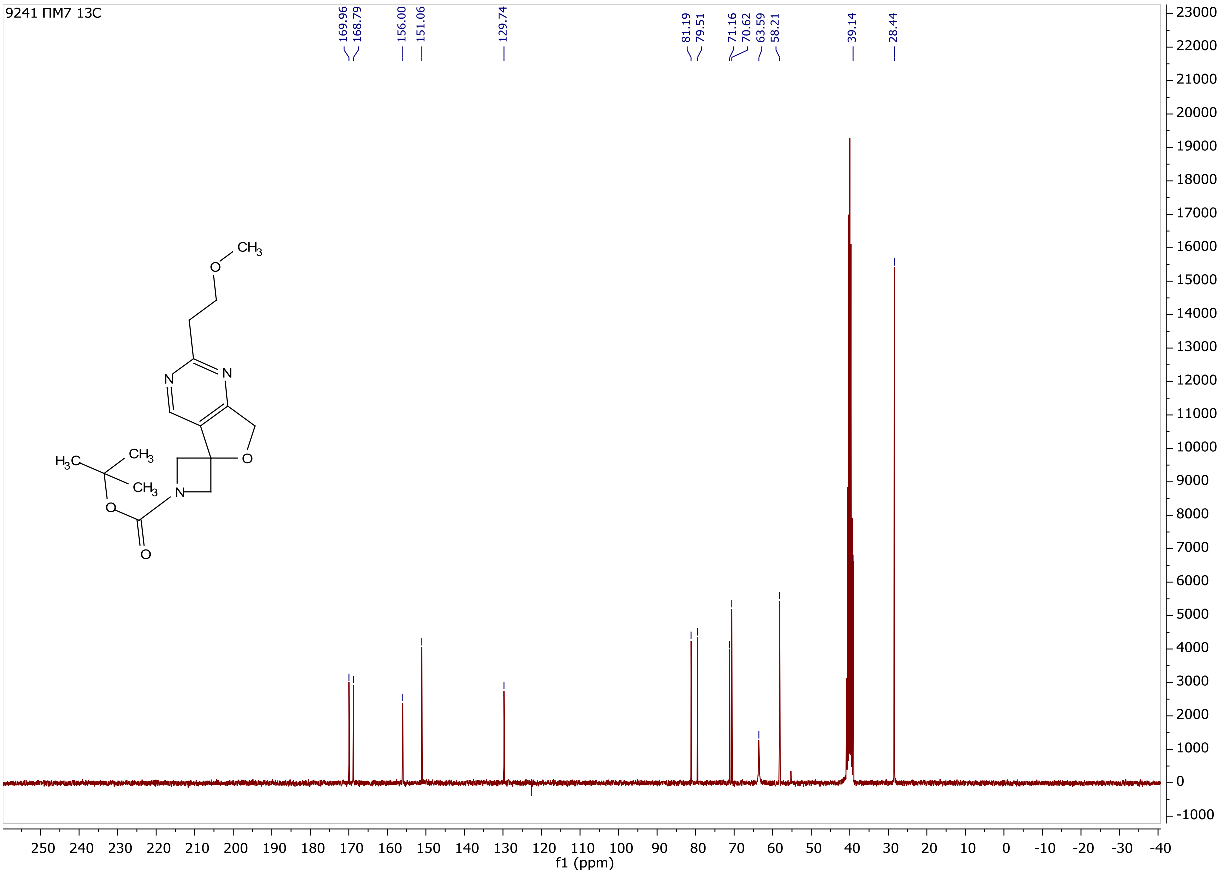

^{1}H and ^{13}C NMR spectra for compound 7l

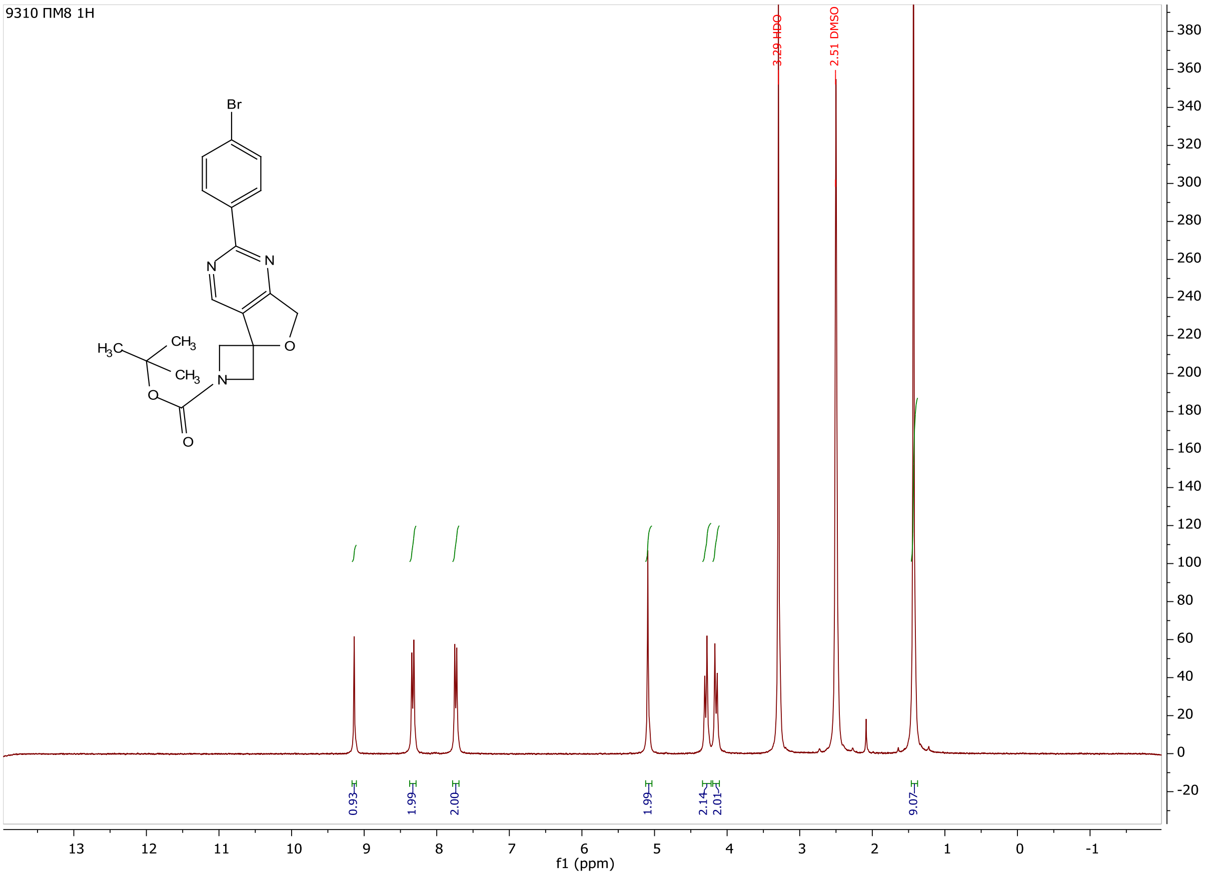

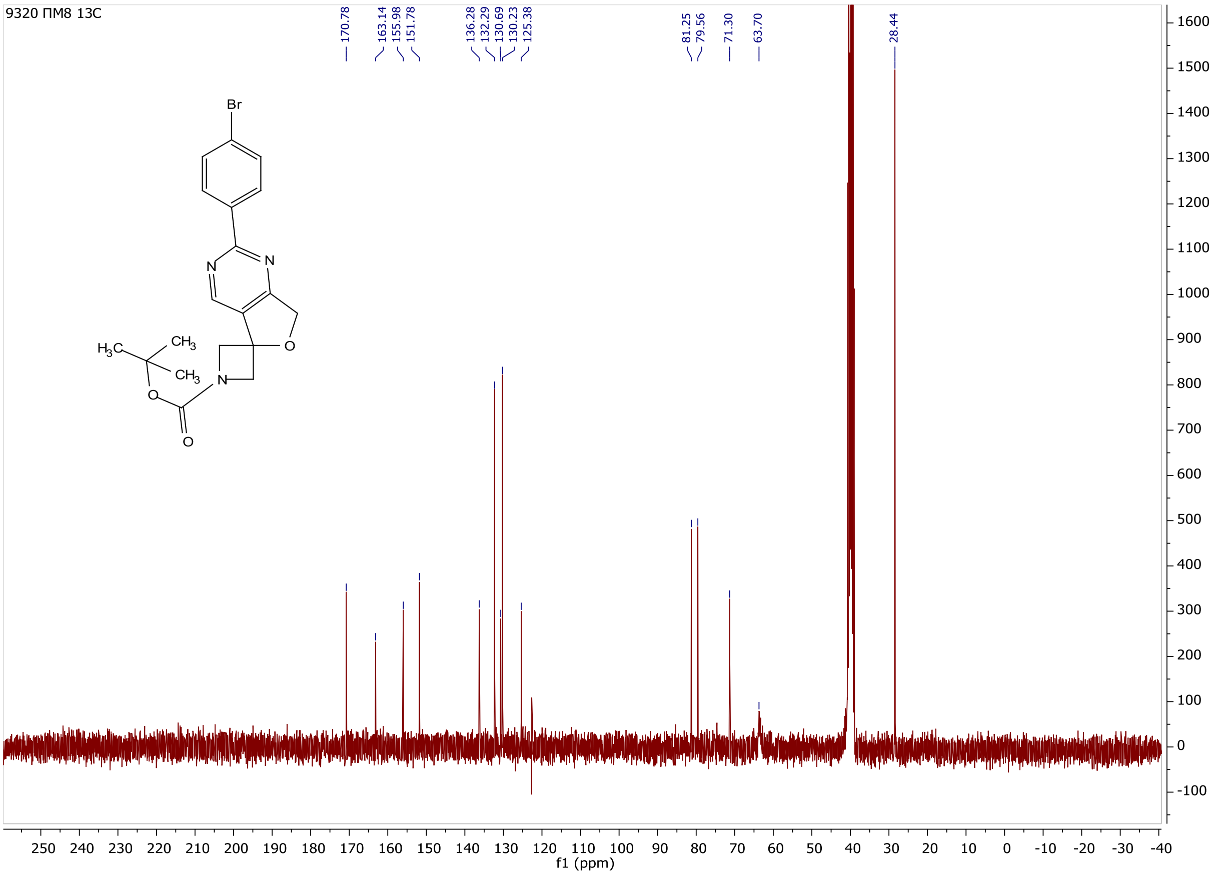

^{1}H and ^{13}C NMR spectra for compound 7m

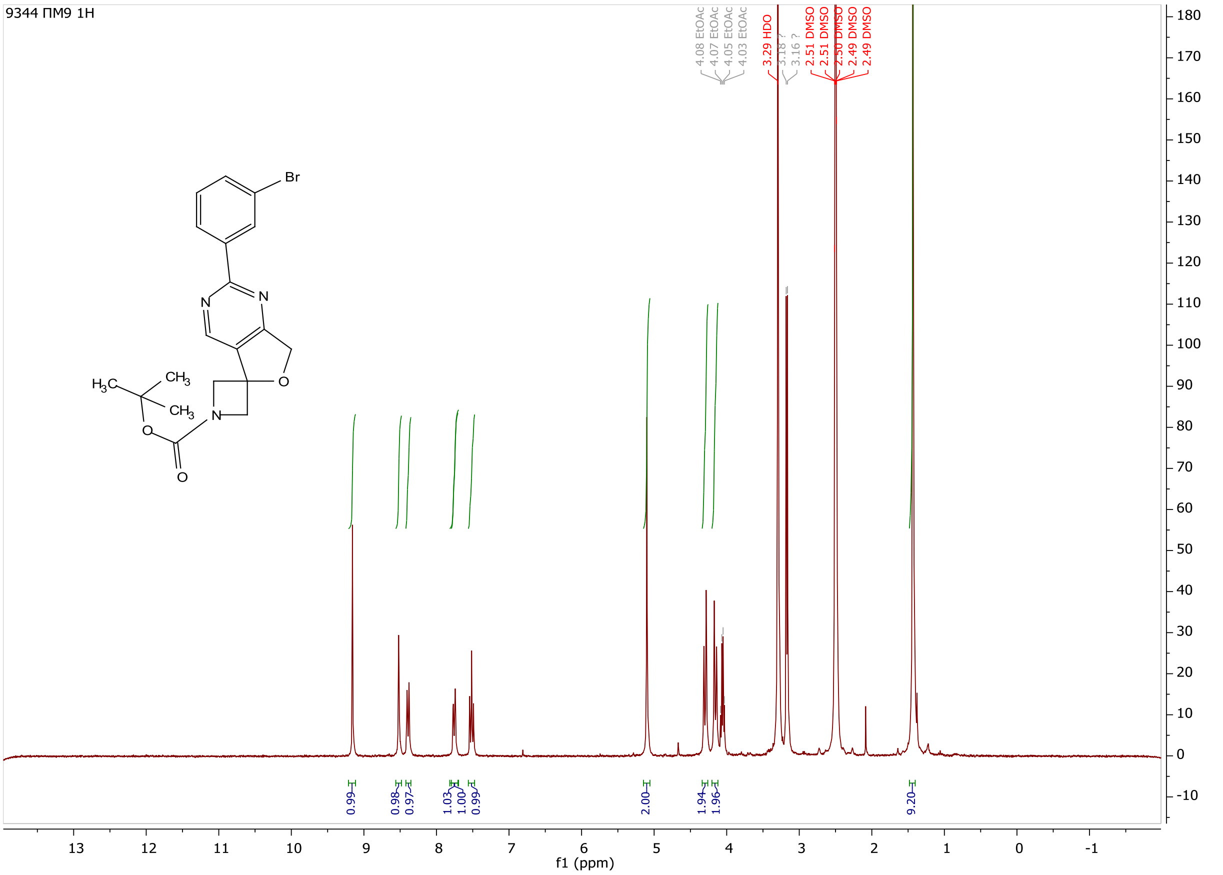

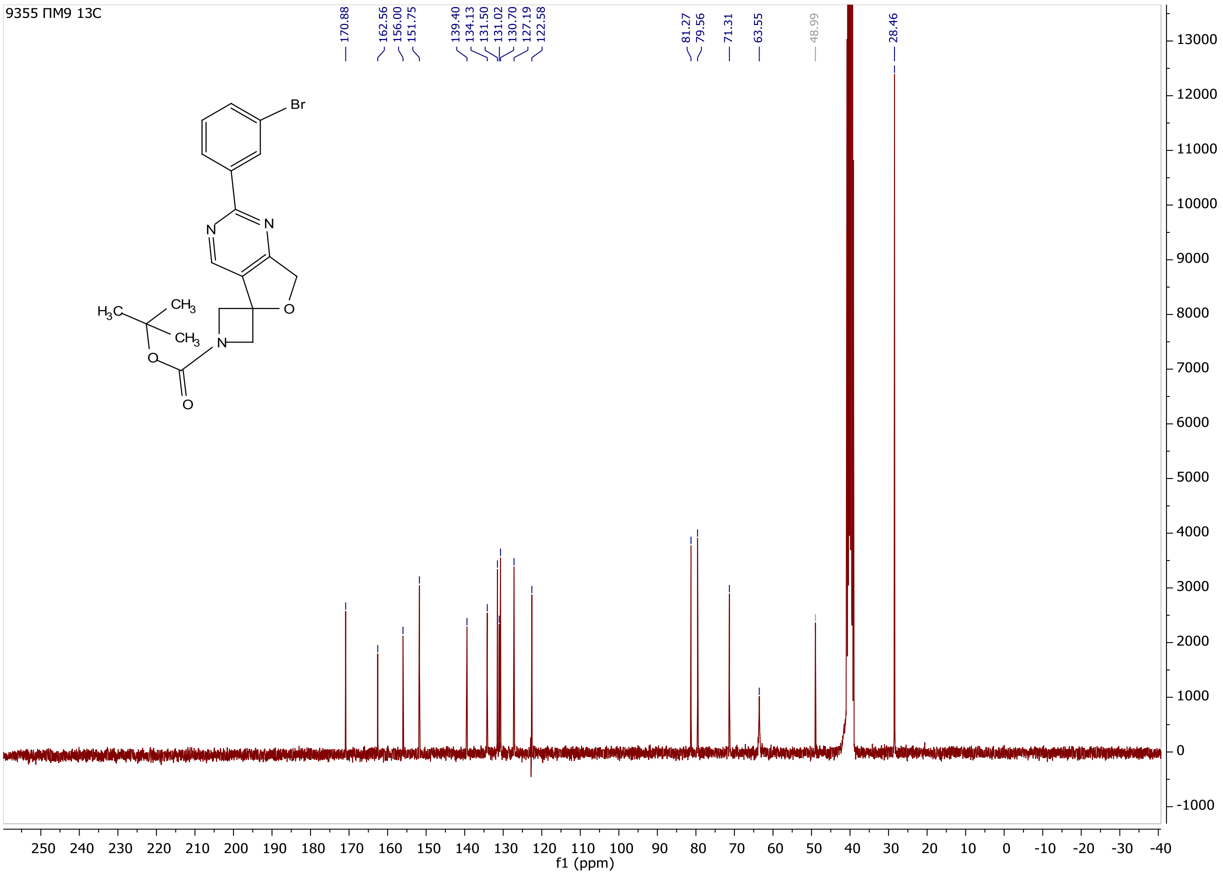

^{1}H and ^{13}C NMR spectra for compound 7n

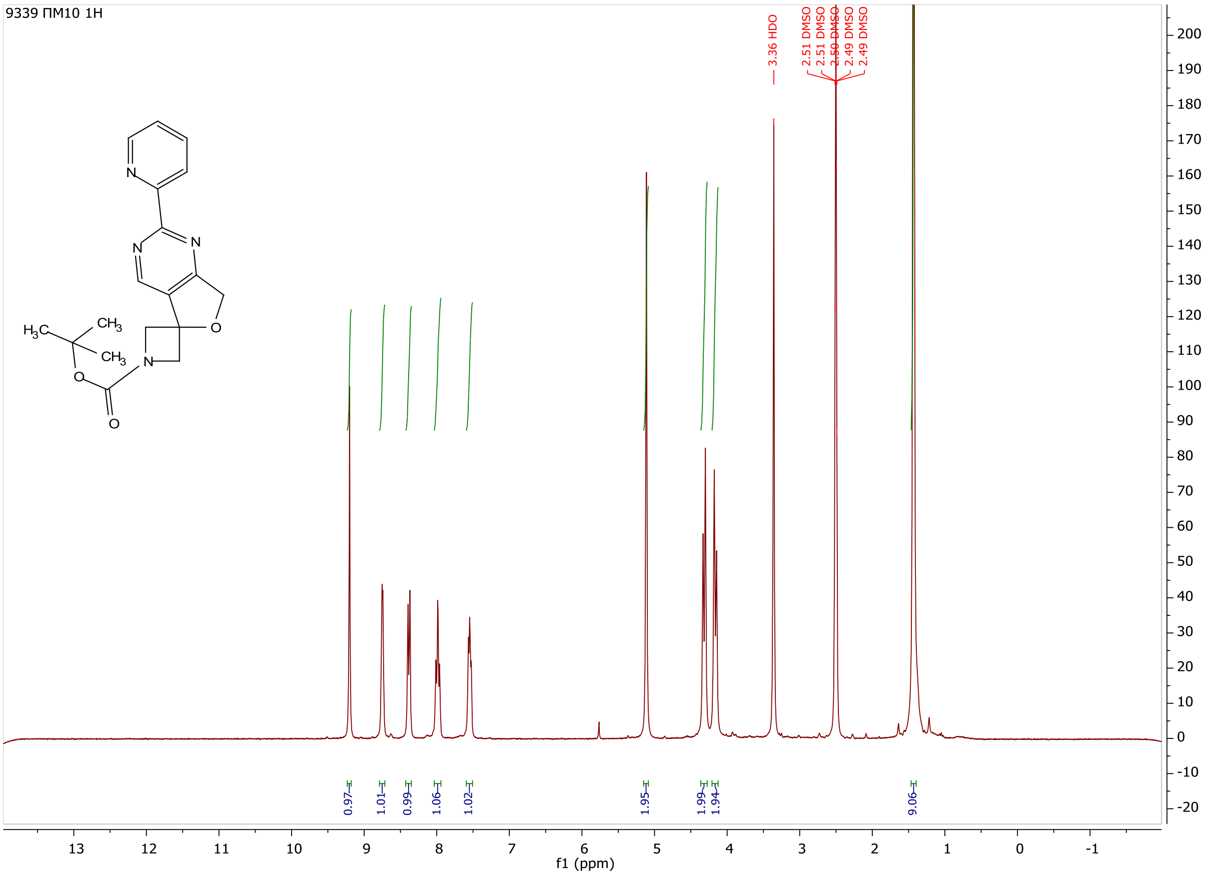

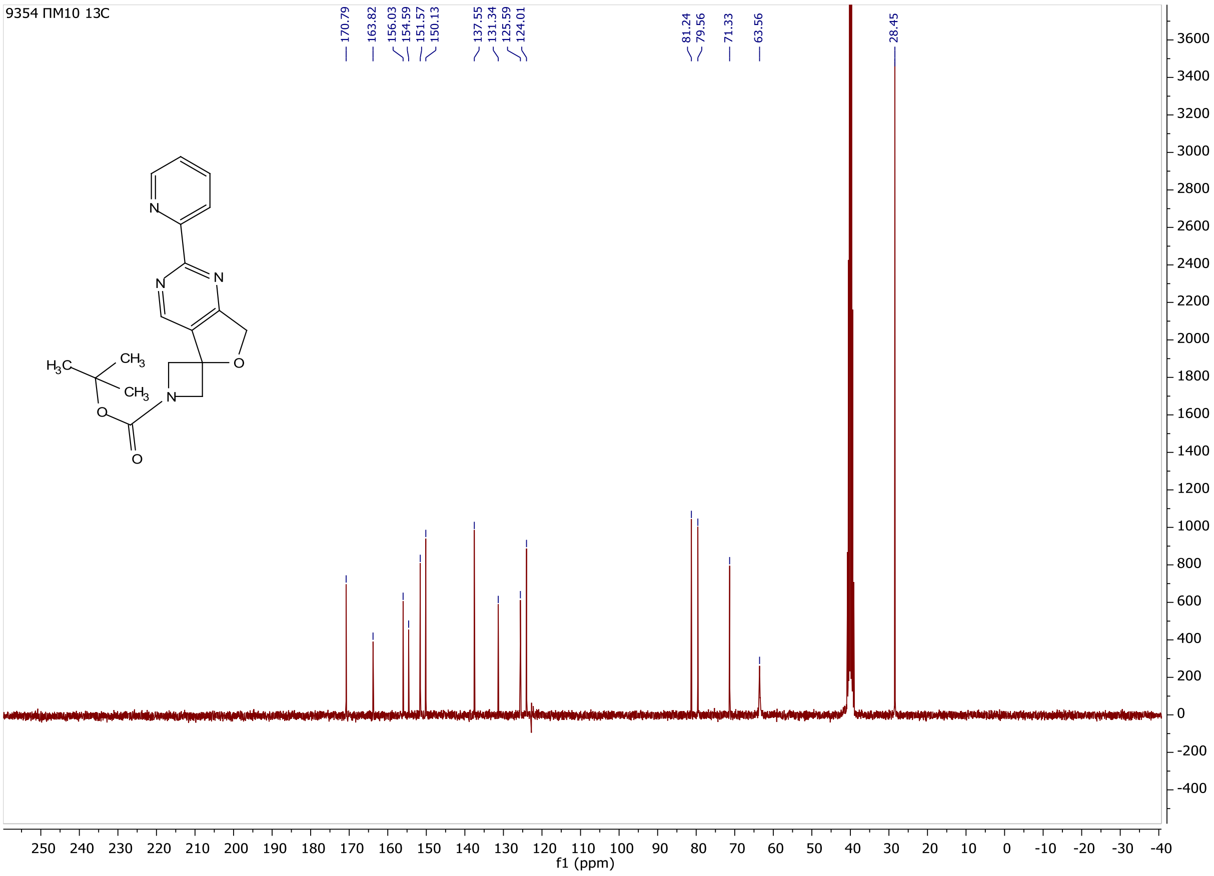

^{1}H and ^{13}C NMR spectra for compound 7o

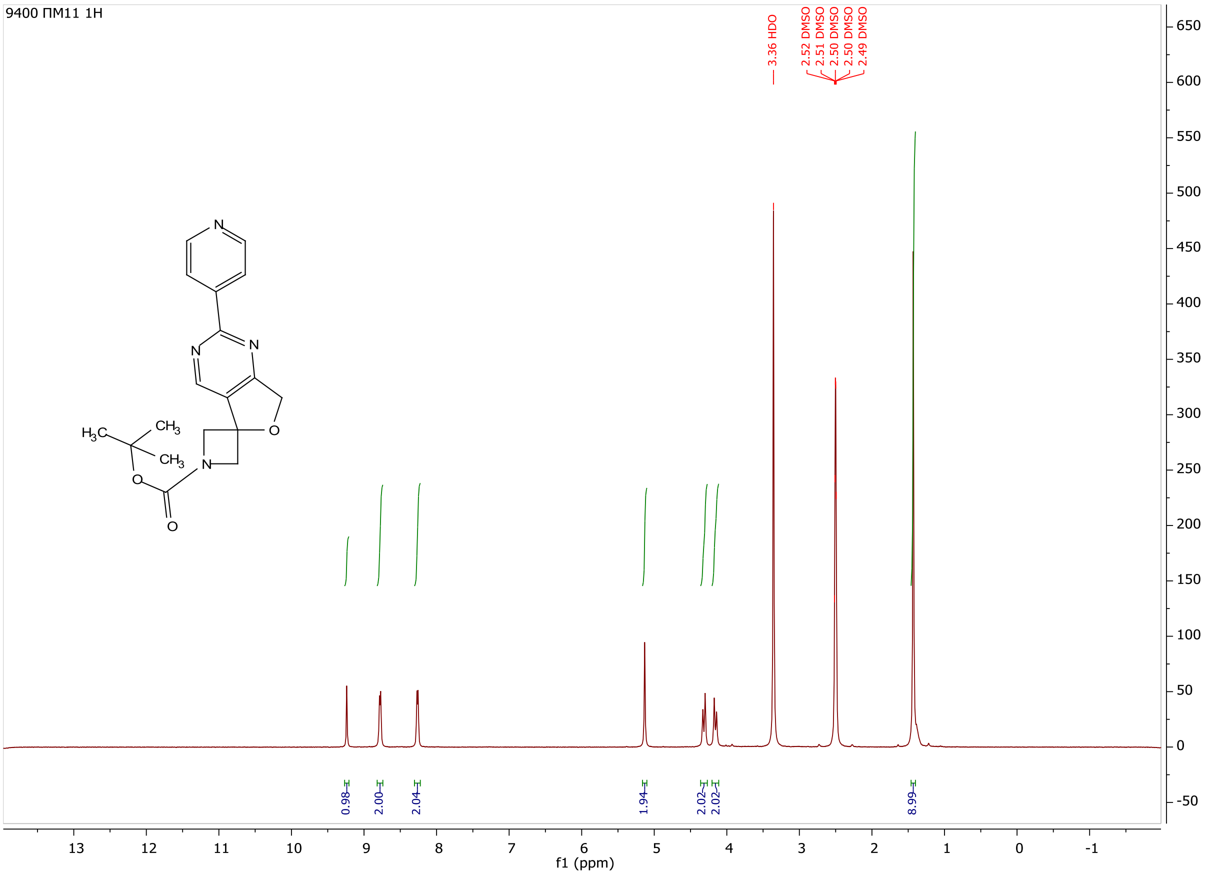

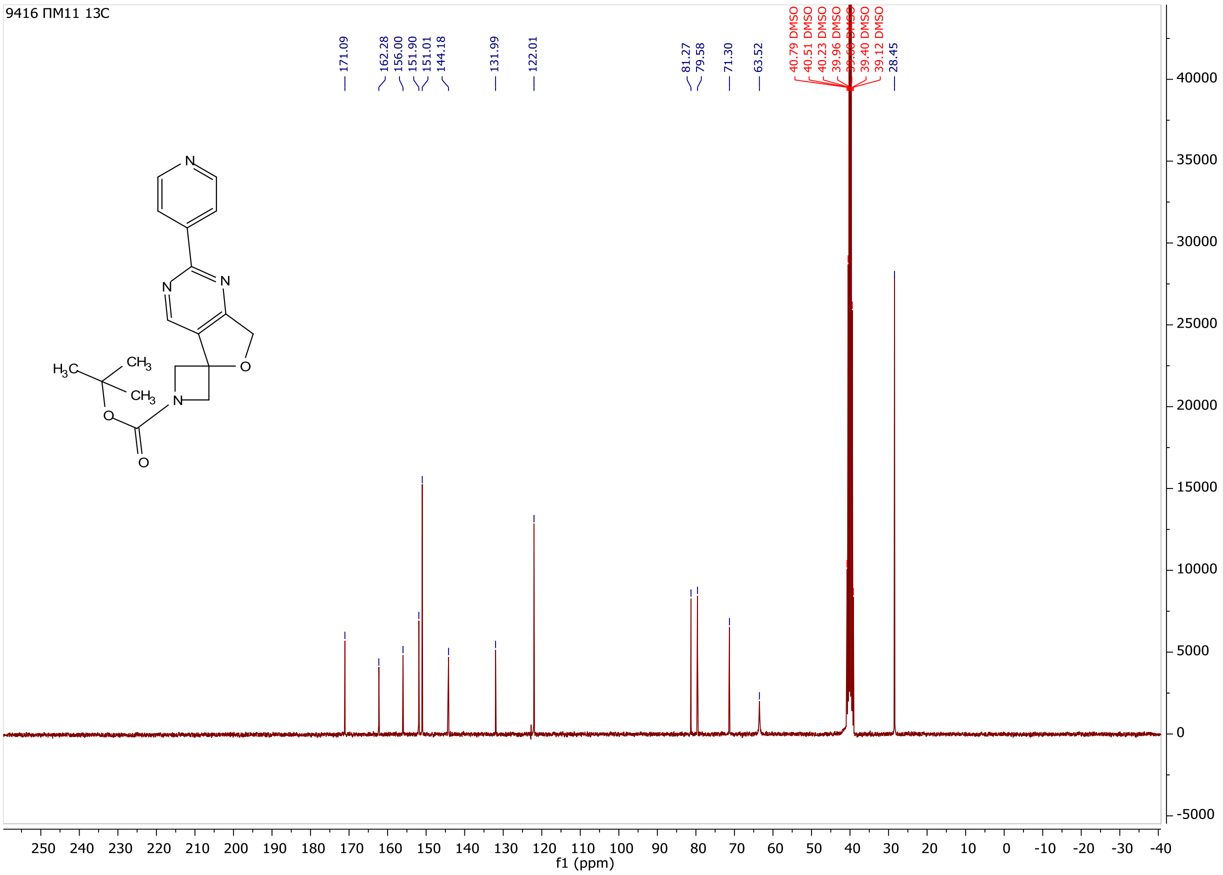

^{1}H and ^{13}C NMR spectra for compound 7p

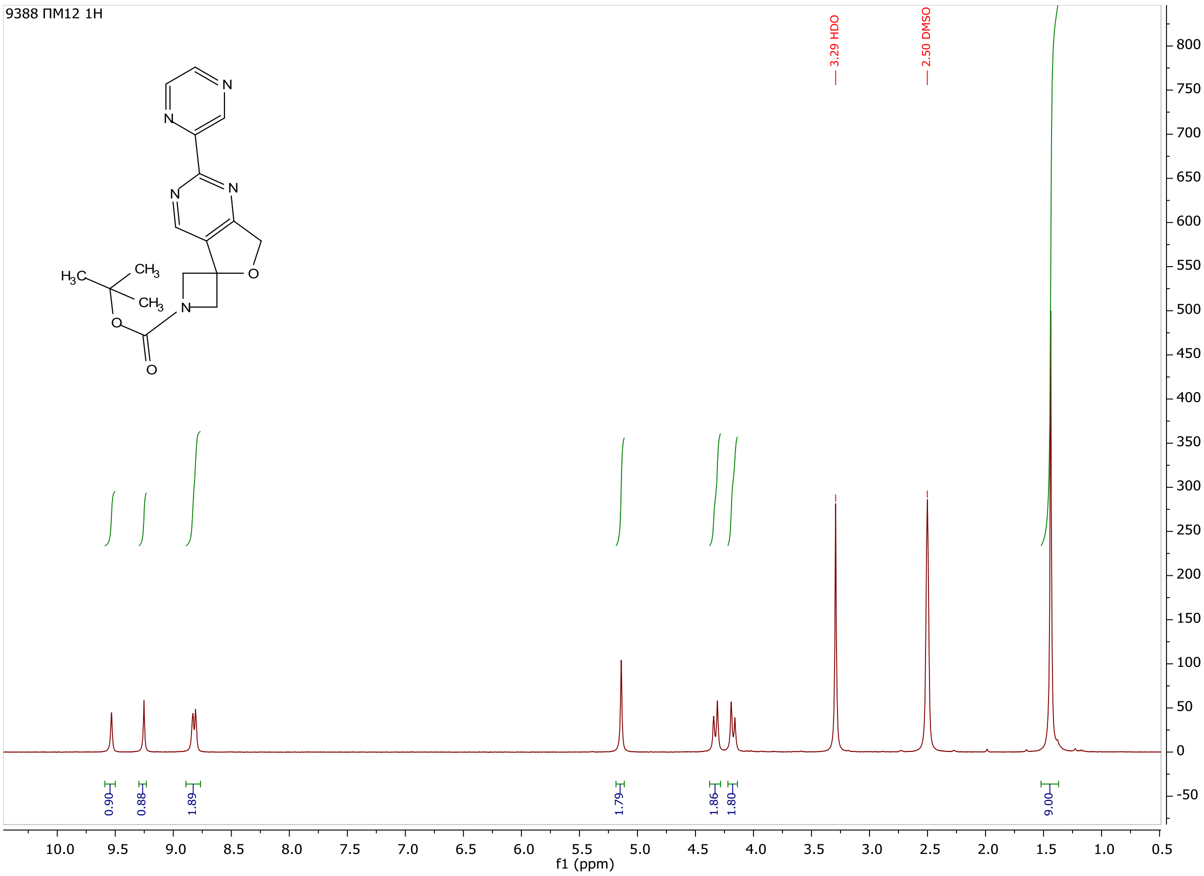

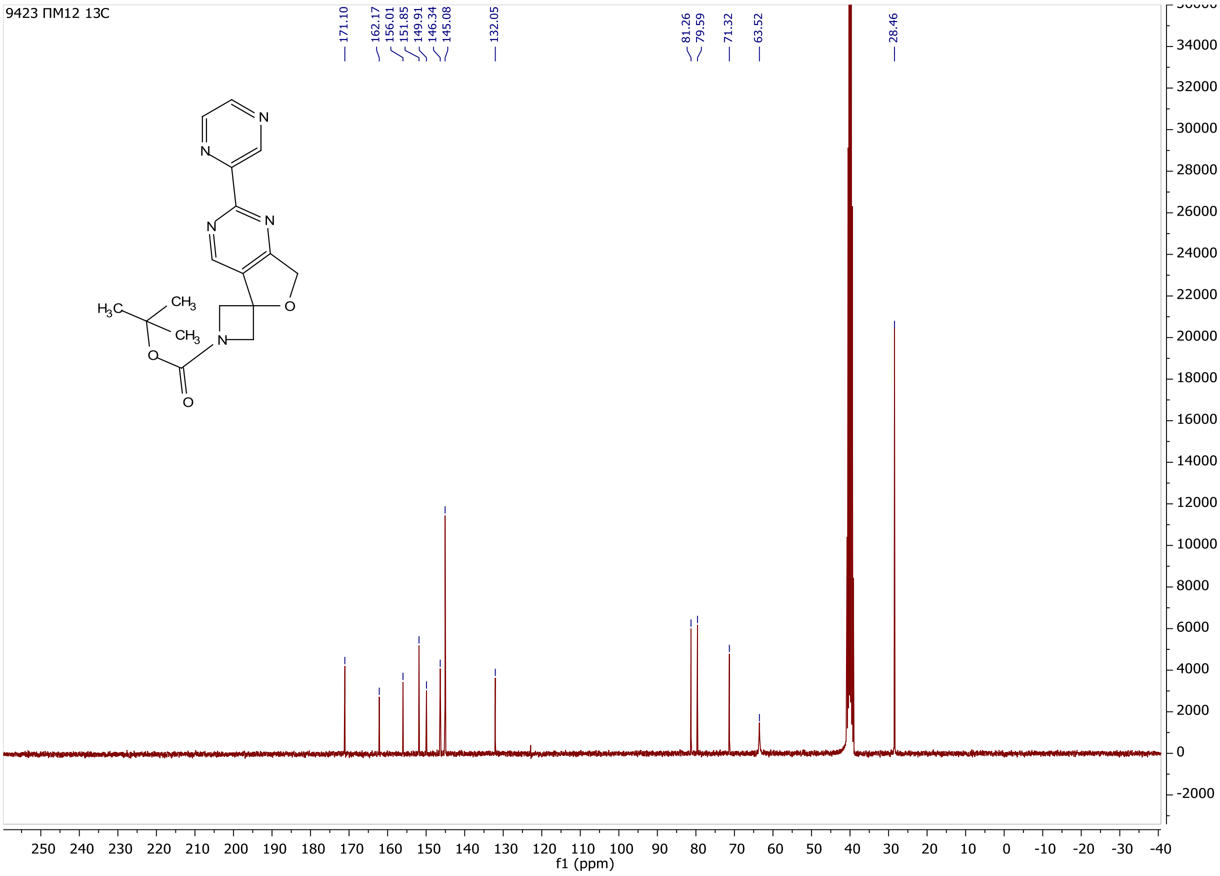

^{1}H and ^{13}C NMR spectra for compound 7q

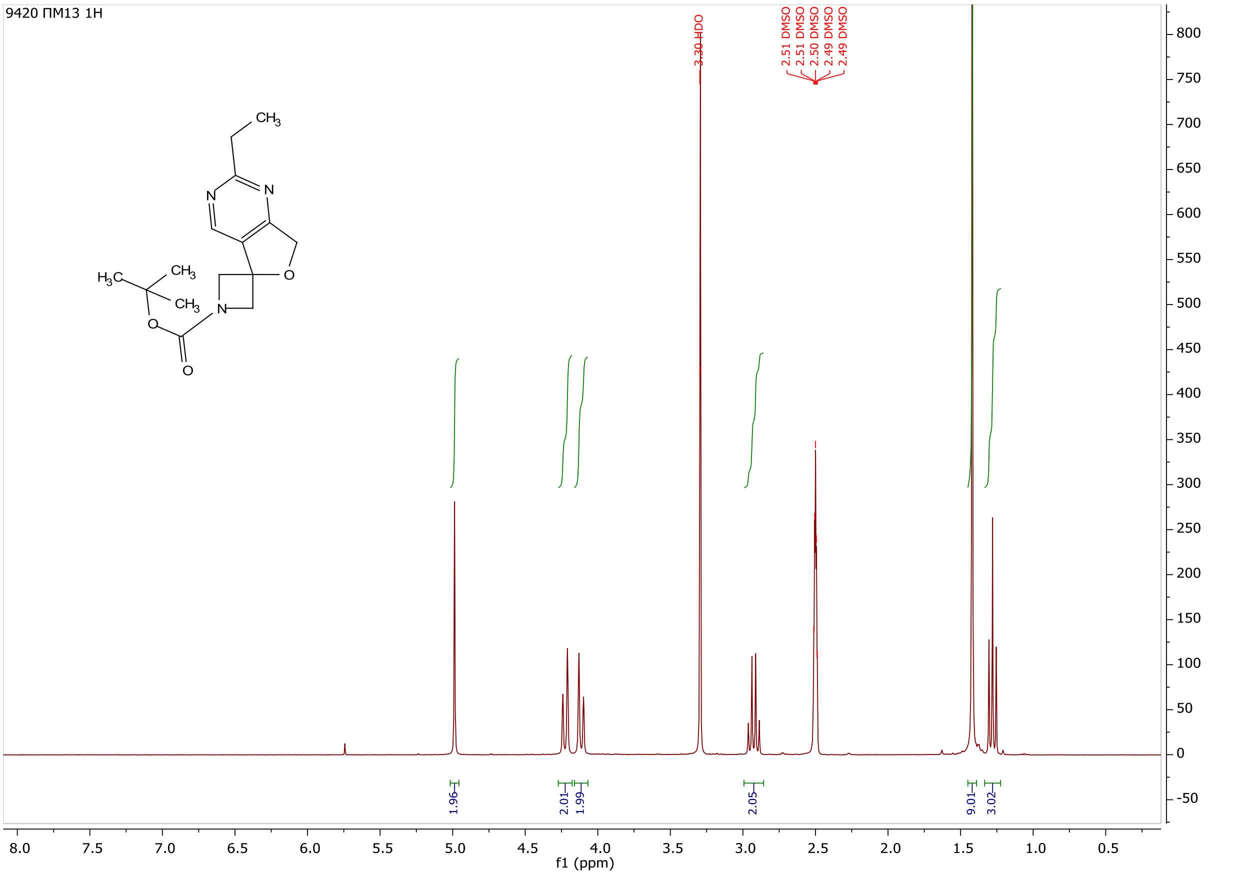

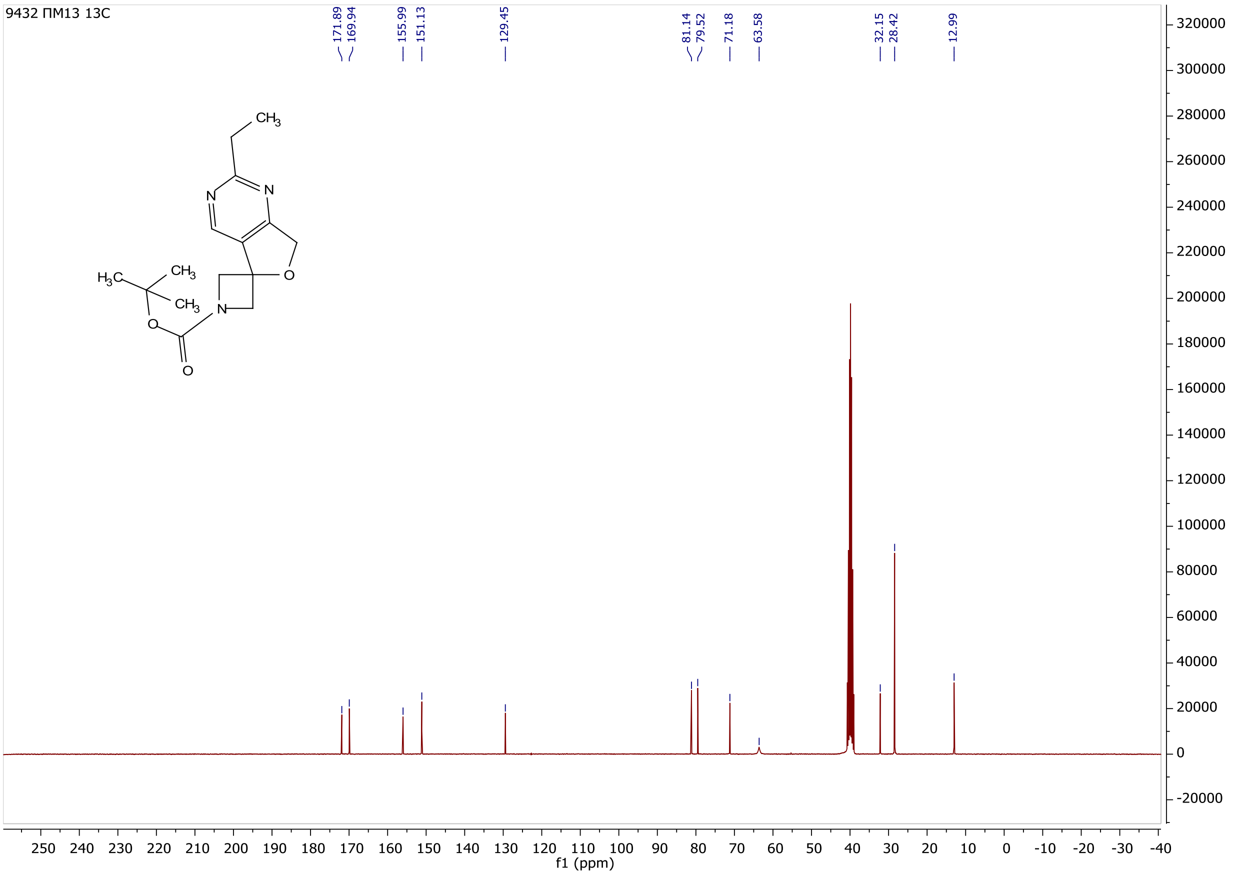

^{1}H and ^{13}C NMR spectra for compound 7r

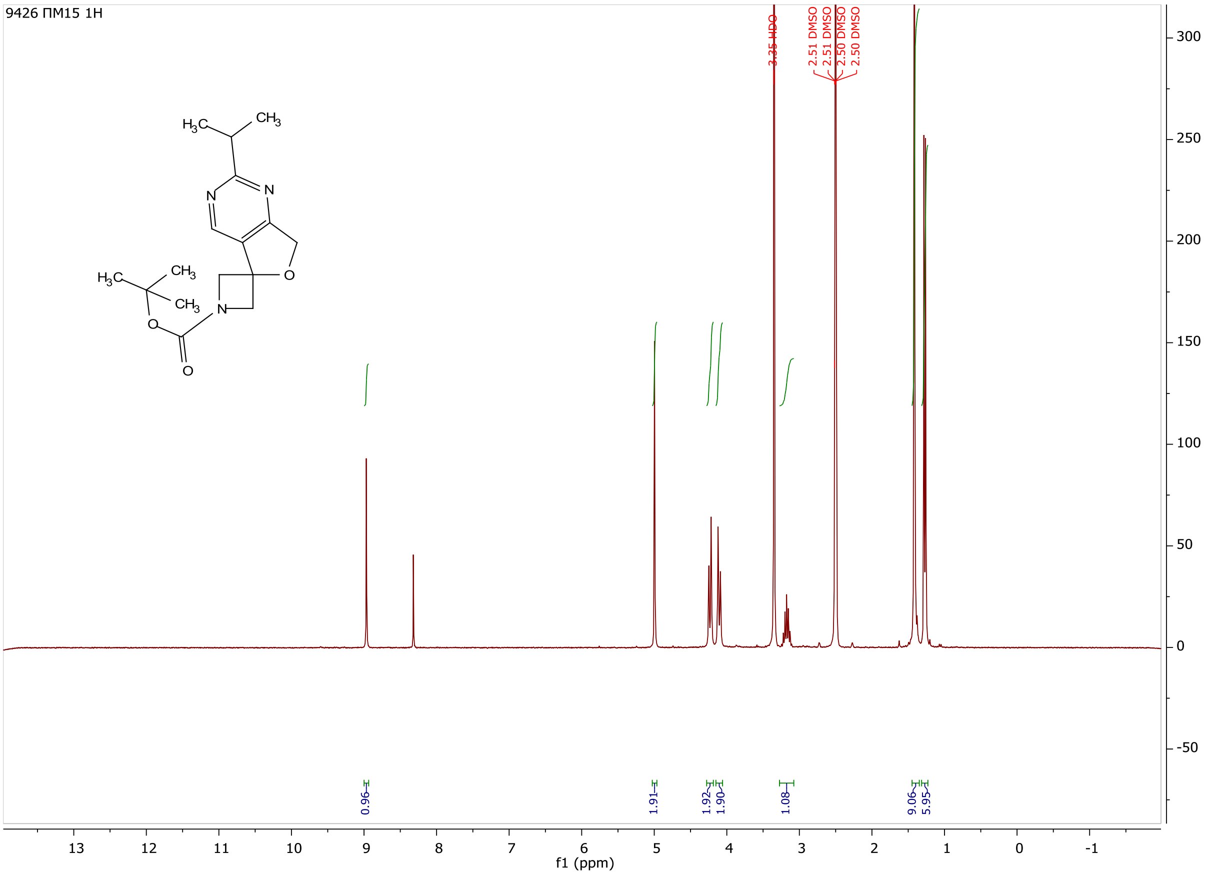

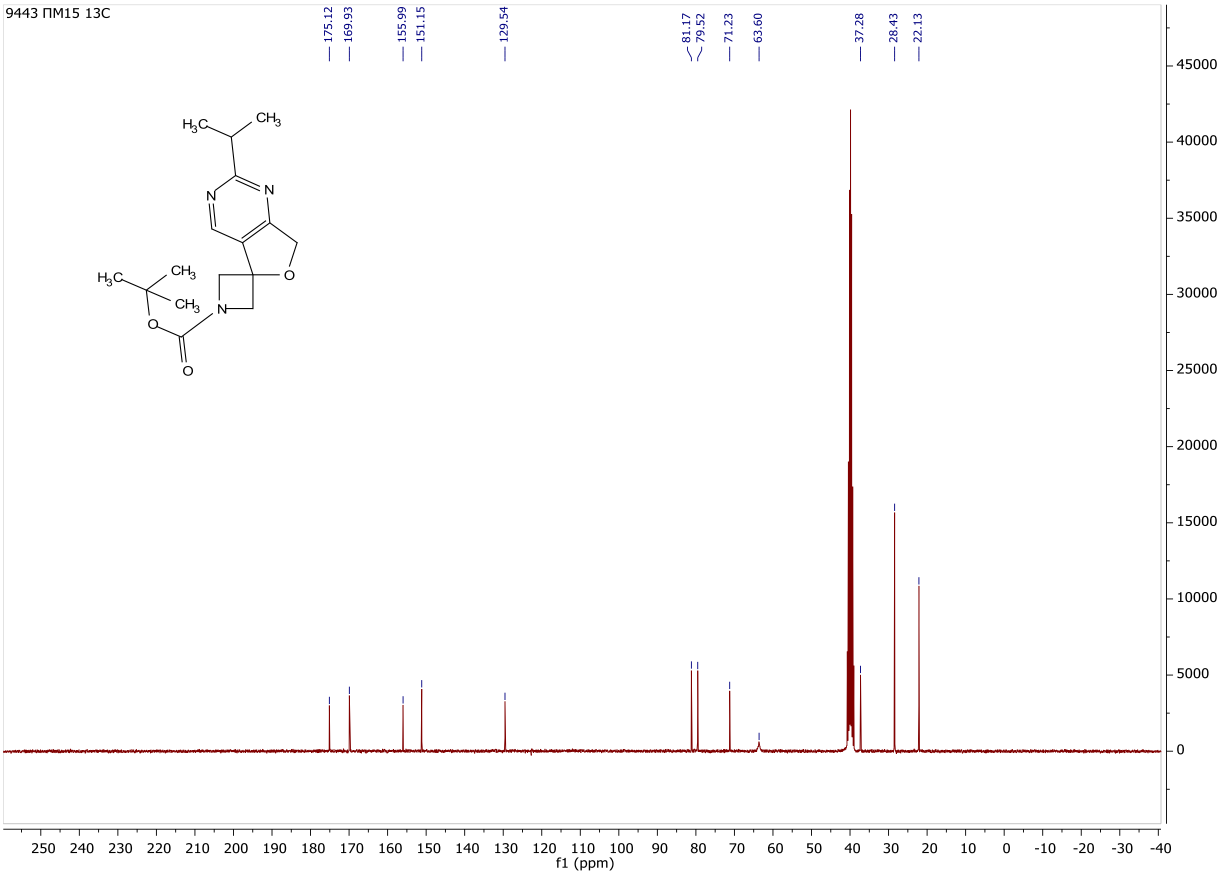

^{1}H and ^{13}C NMR spectra for compound 3a

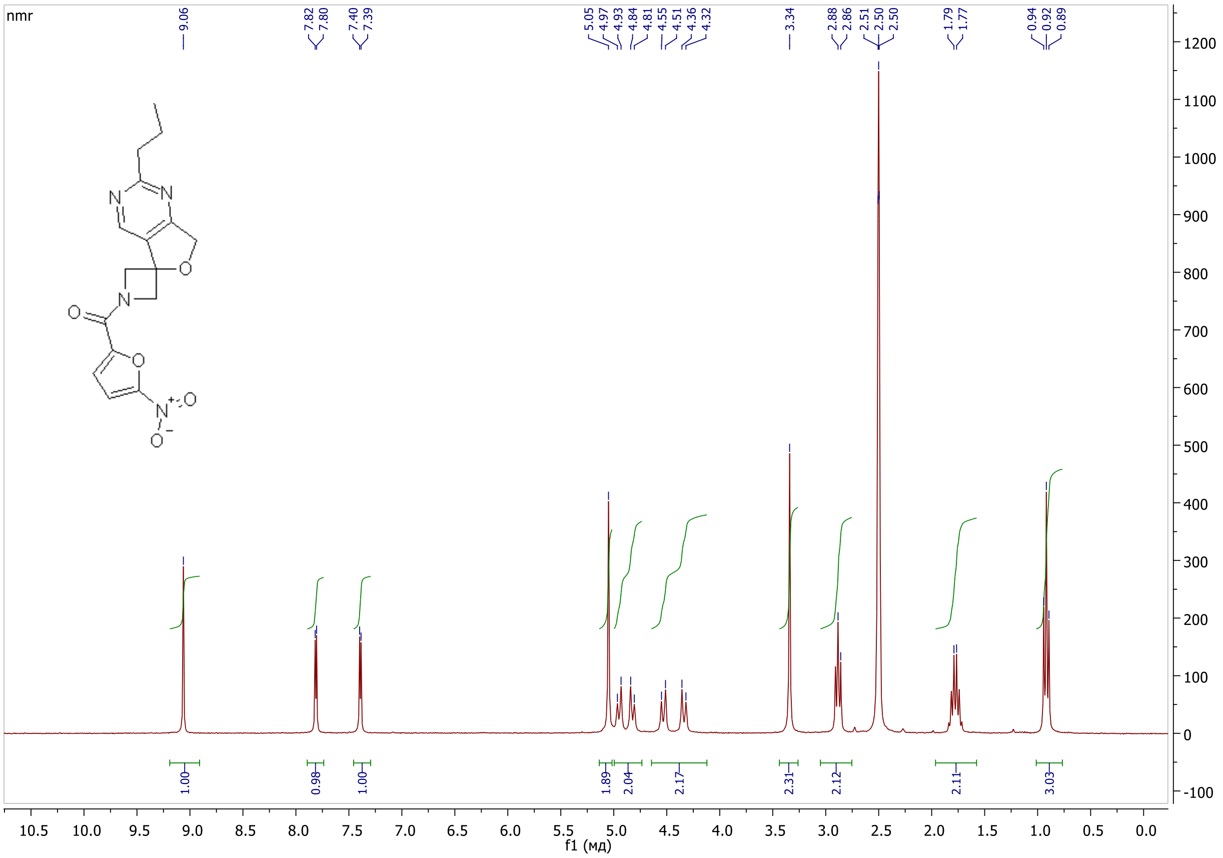

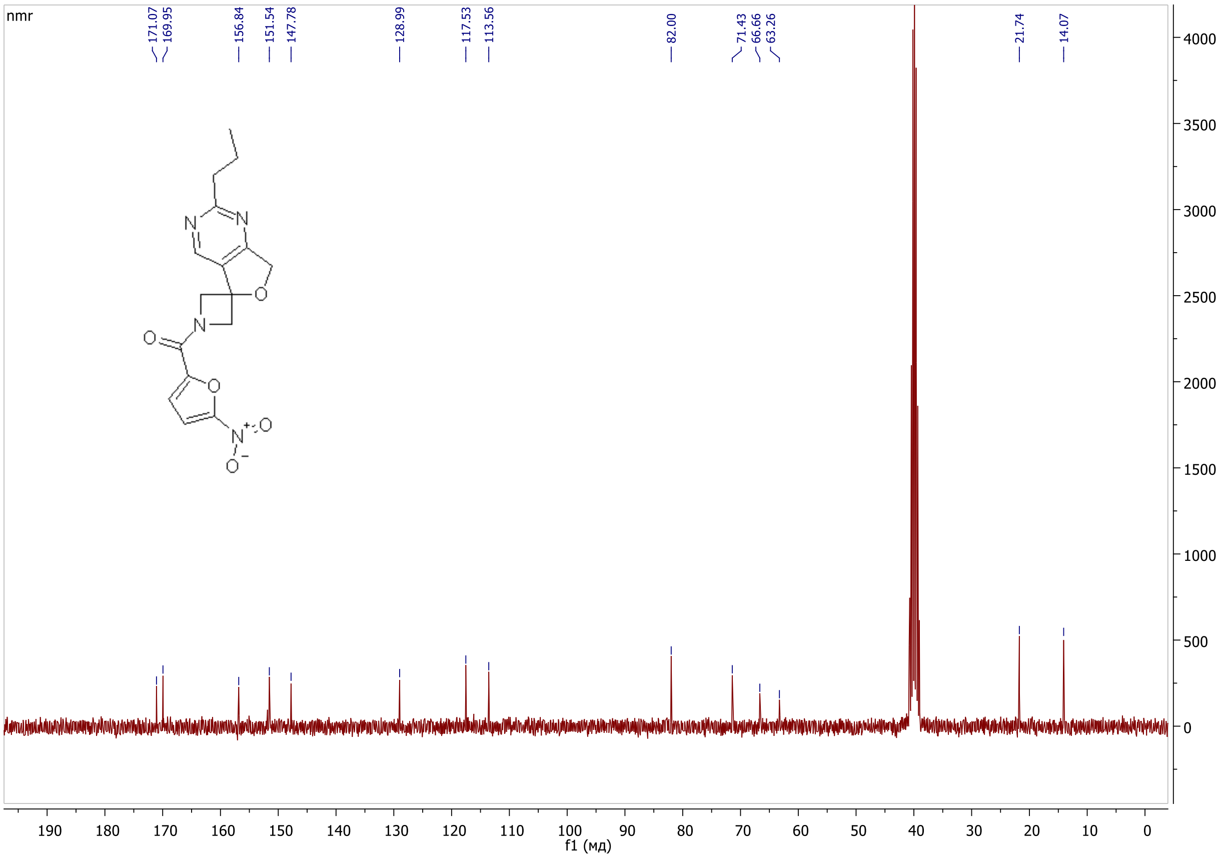

^{1}H and ^{13}C NMR spectra for compound 3b

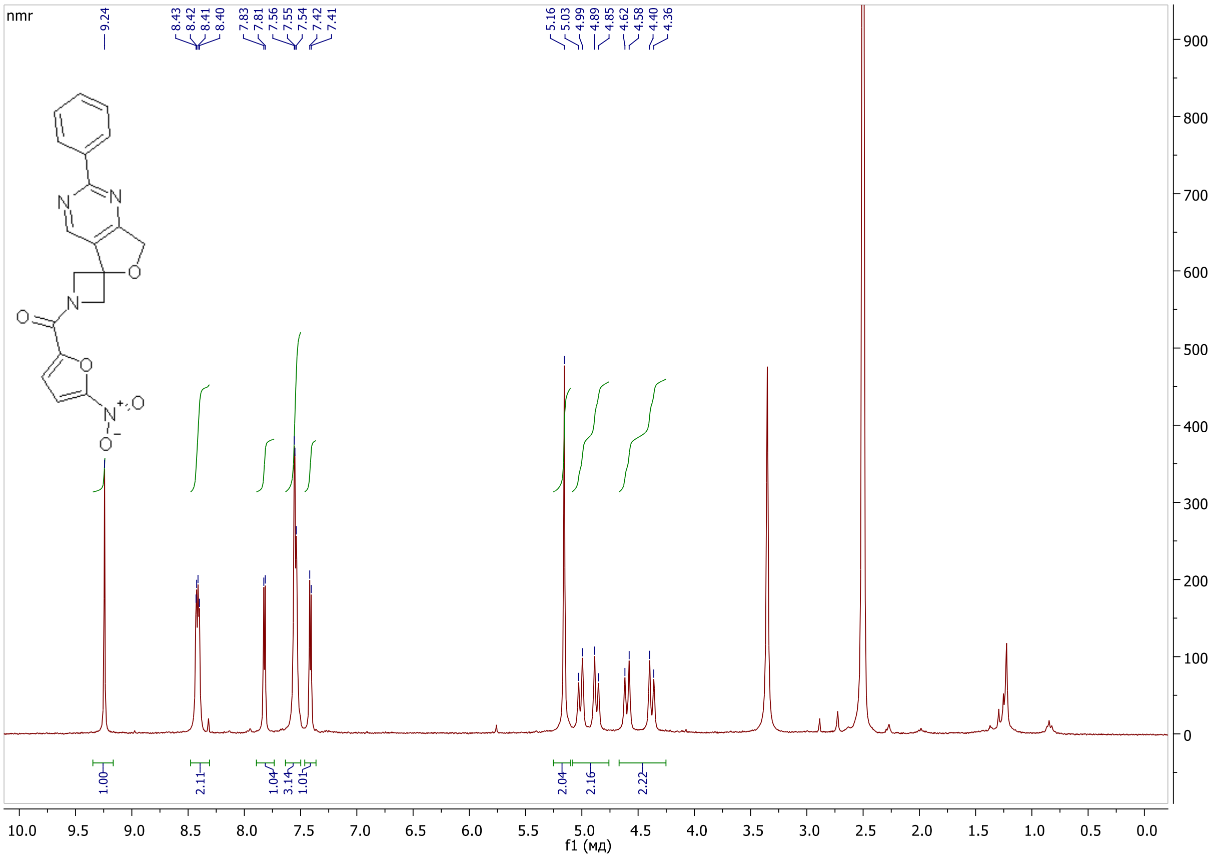

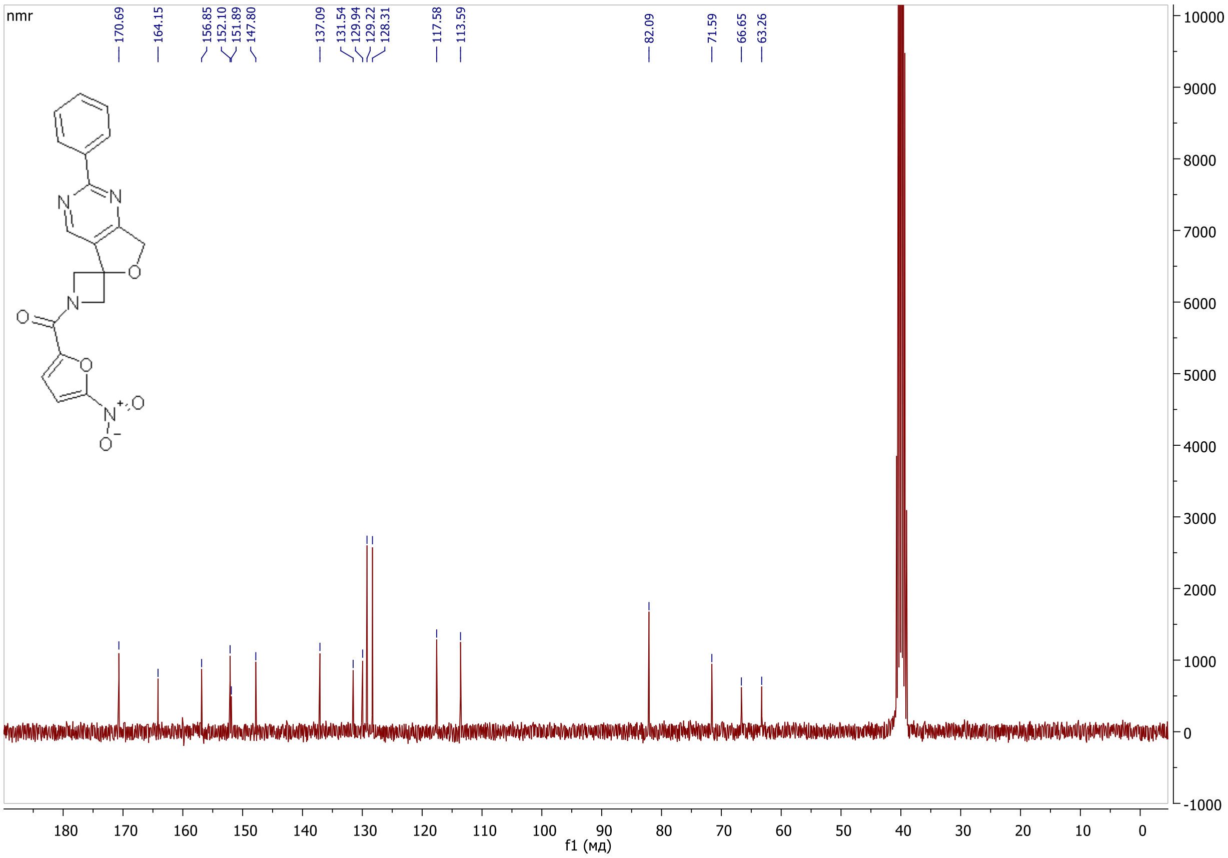

^{1}H and ^{13}C NMR spectra for compound 3c

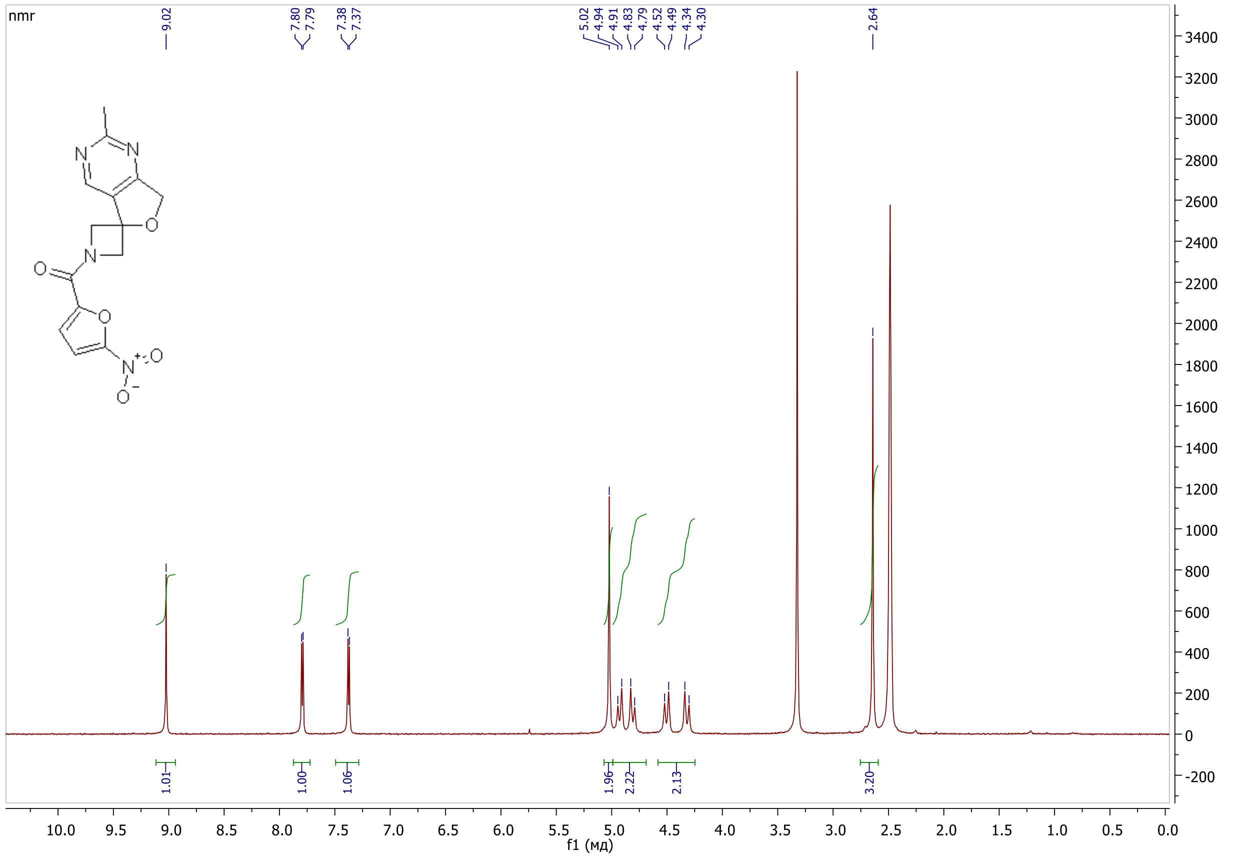

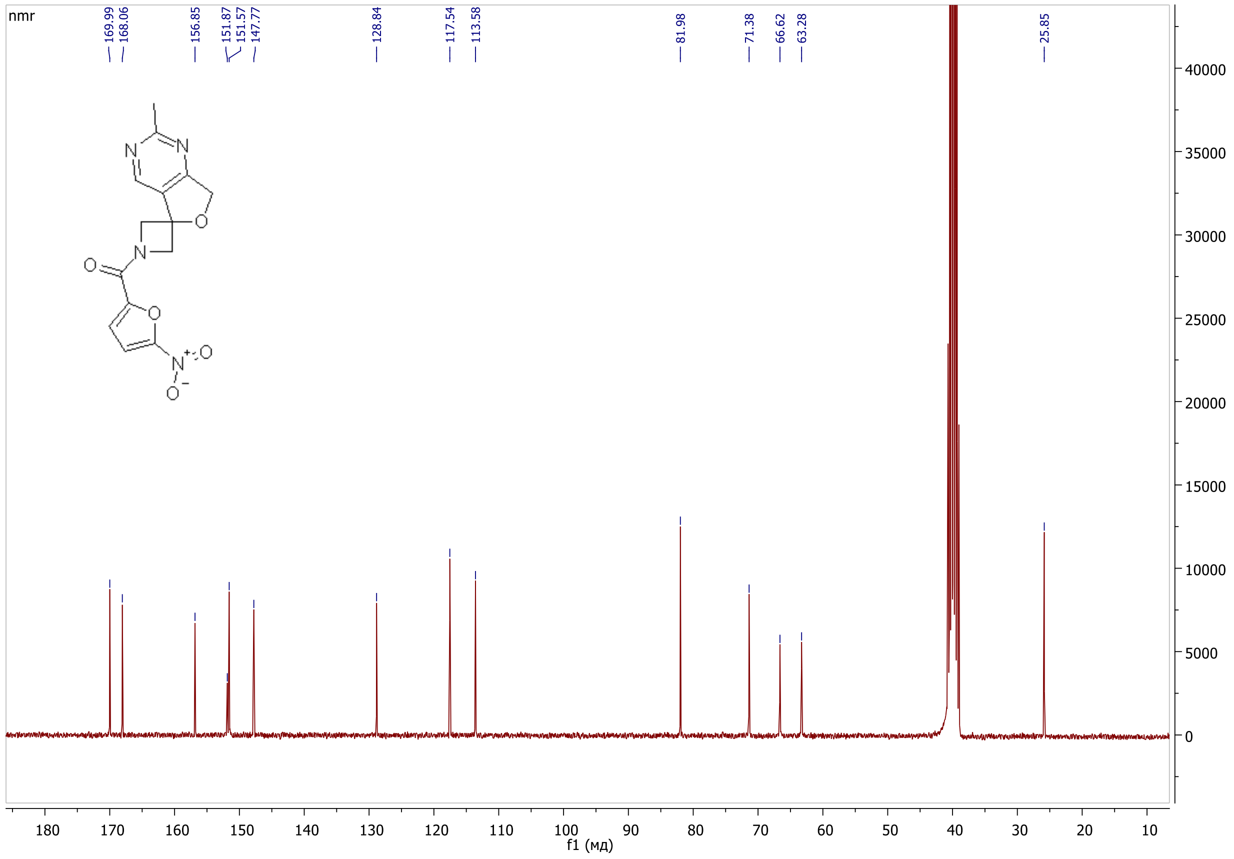

^{1}H and ^{13}C NMR spectra for compound 3d

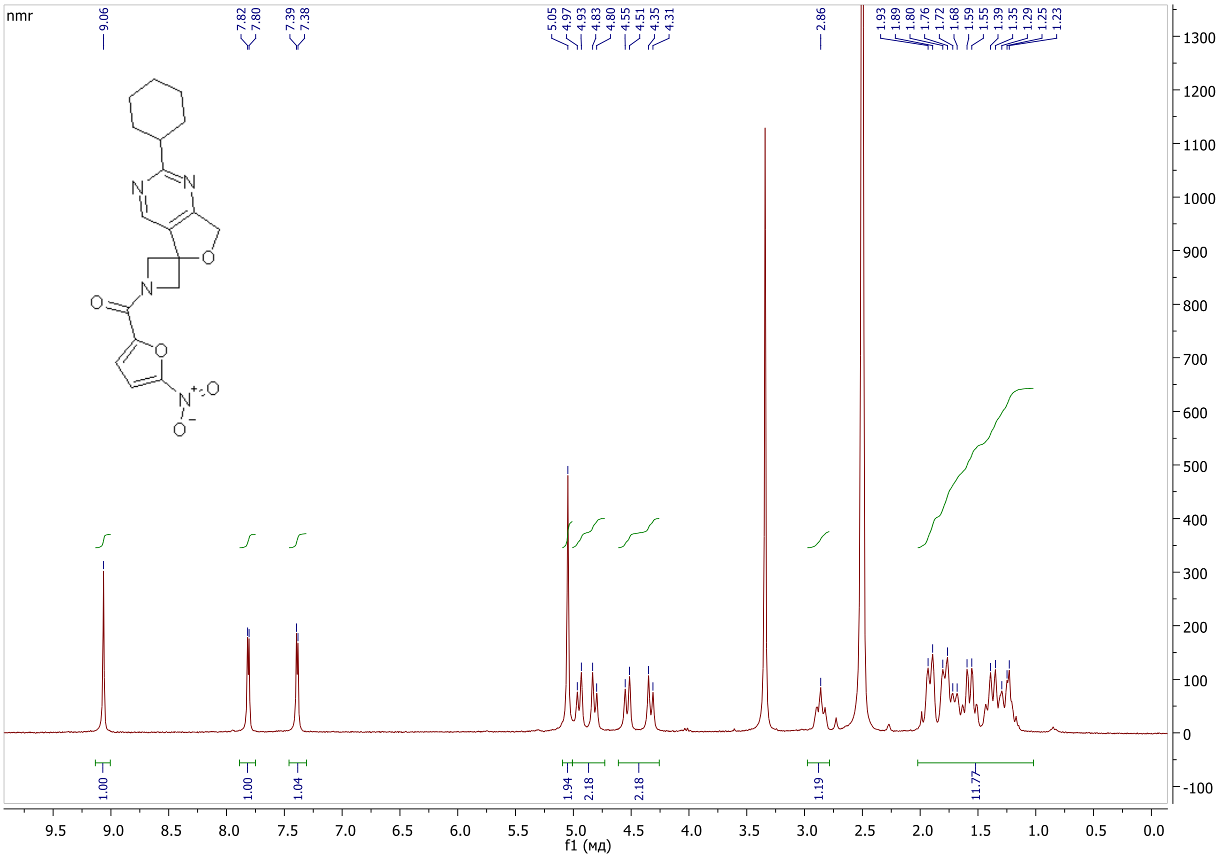

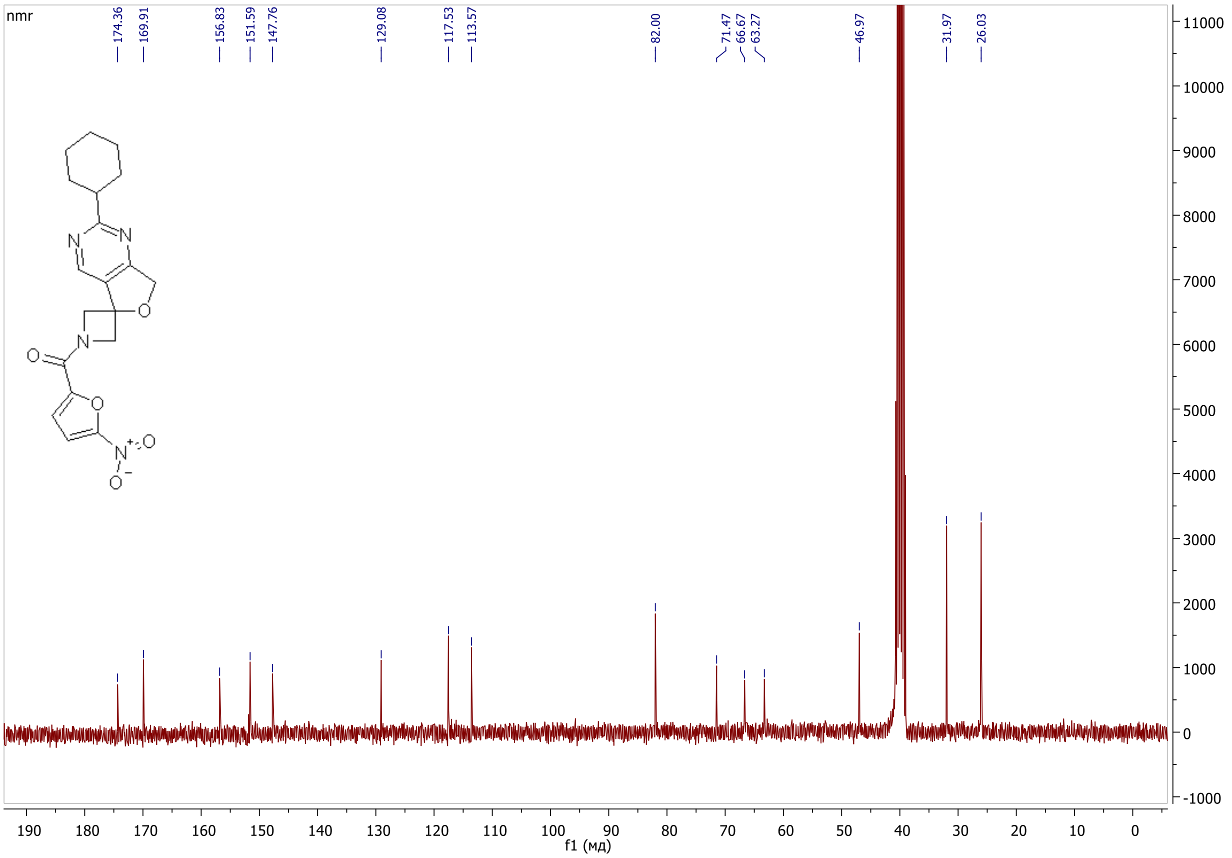

^{1}H and ^{13}C NMR spectra for compound 3e

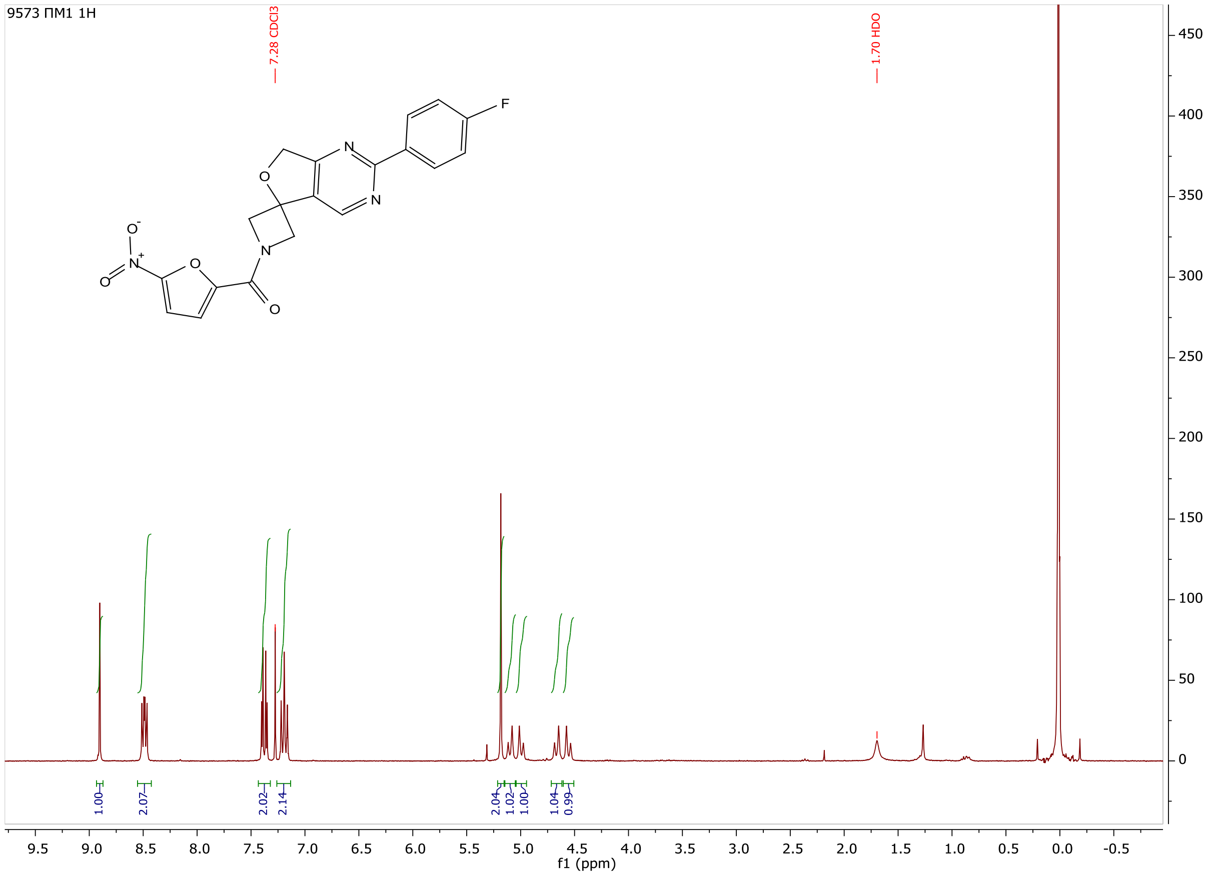

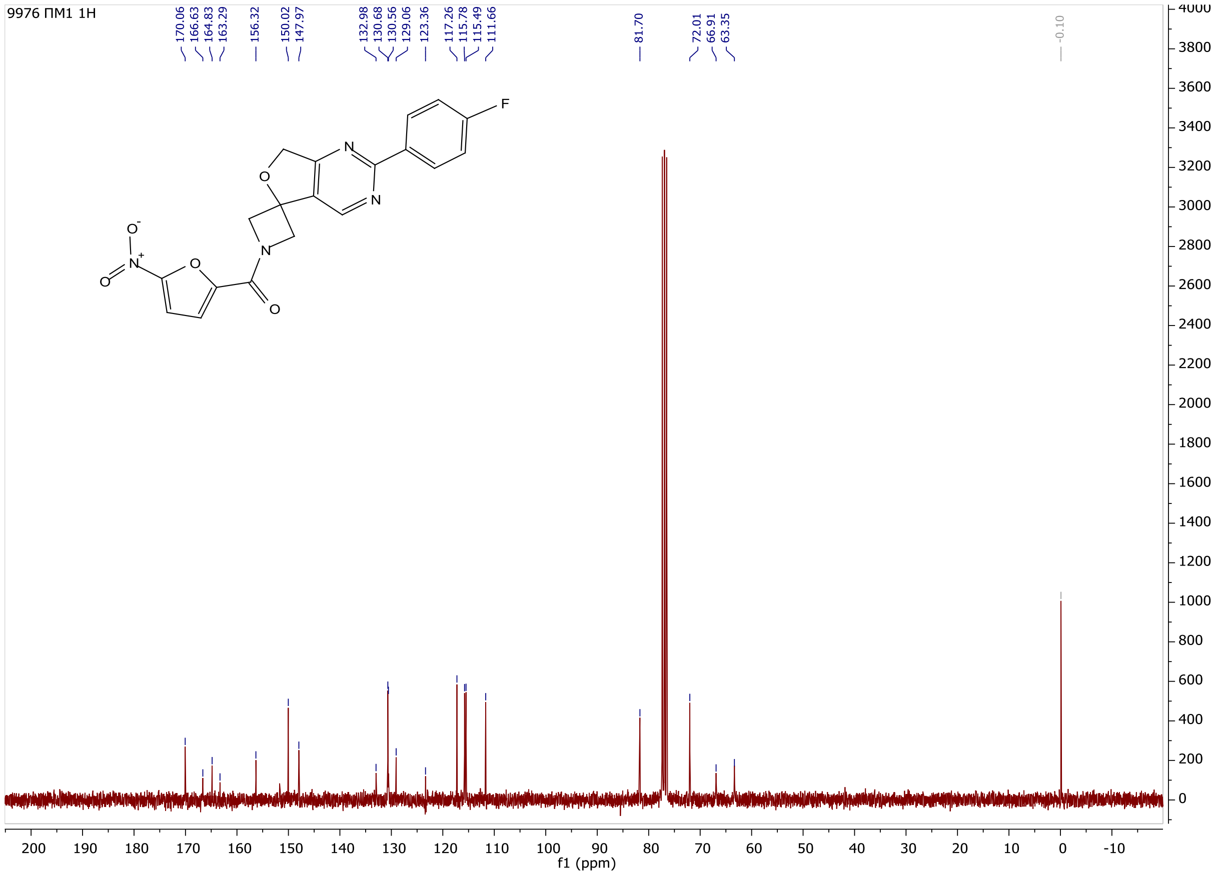

^{1}H and ^{13}C NMR spectra for compound 3f

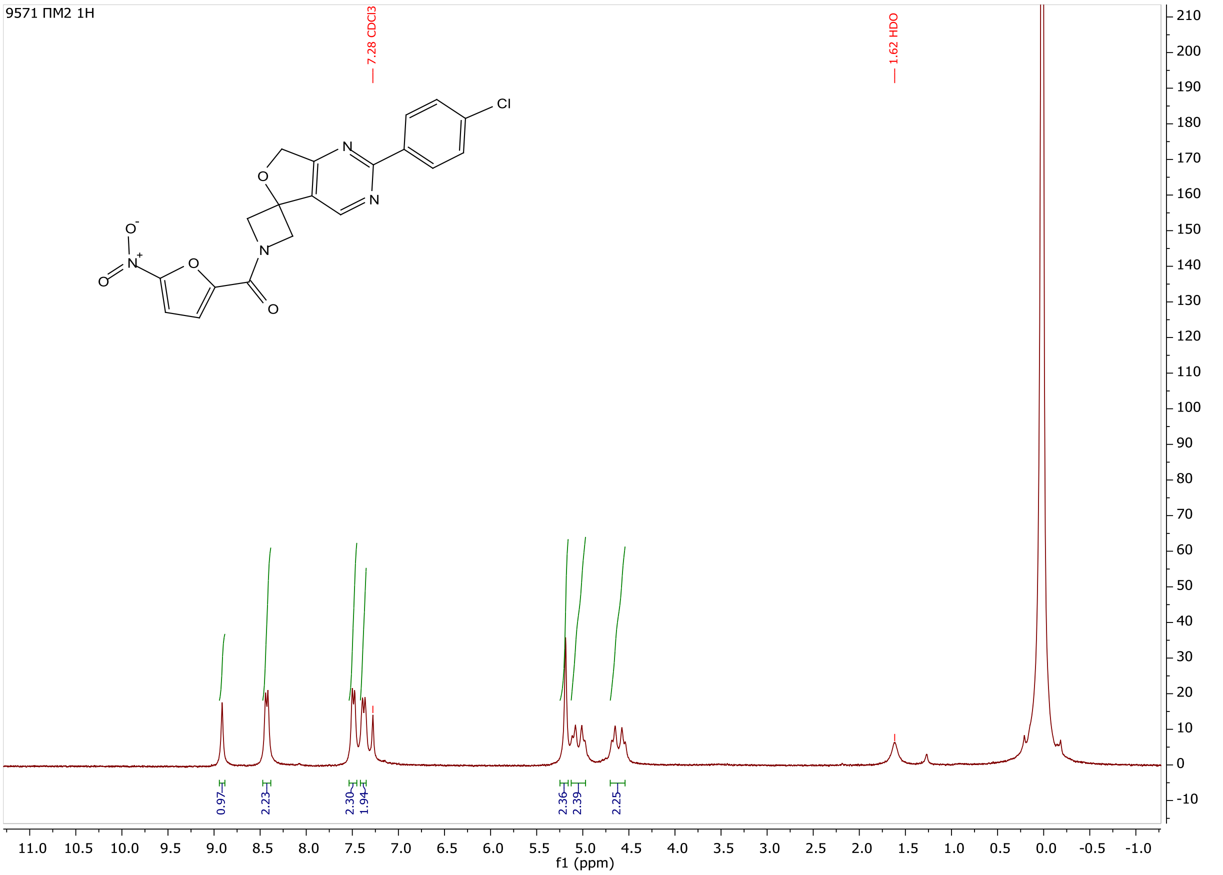

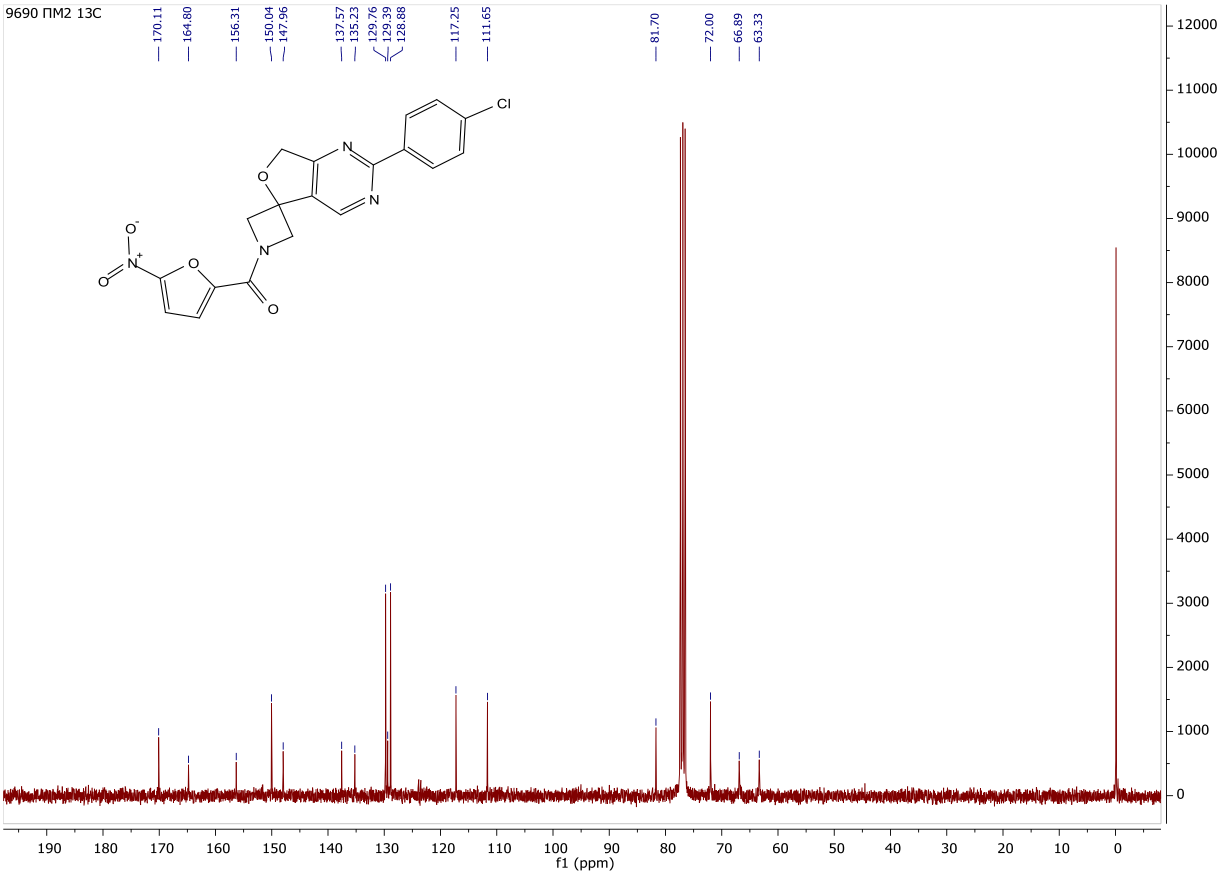

^{1}H and ^{13}C NMR spectra for compound 3g

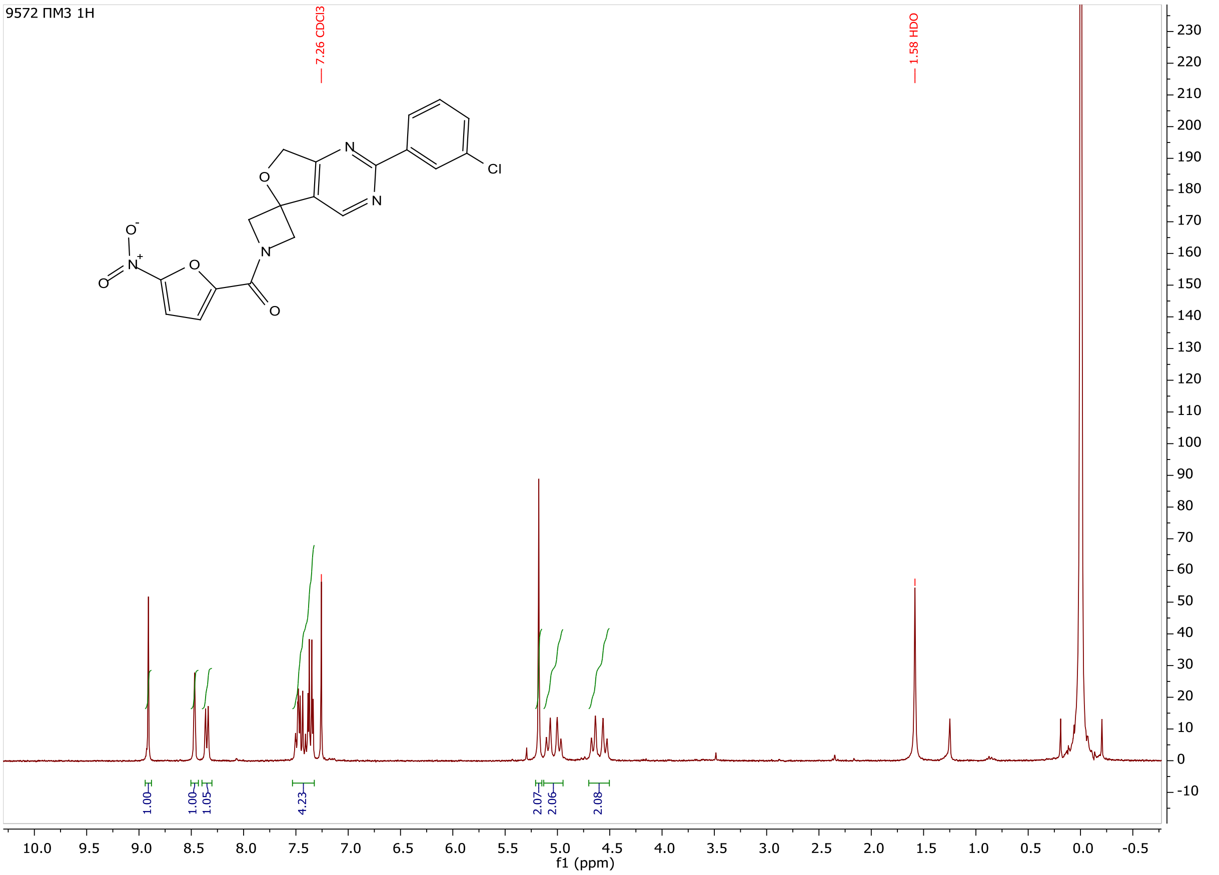

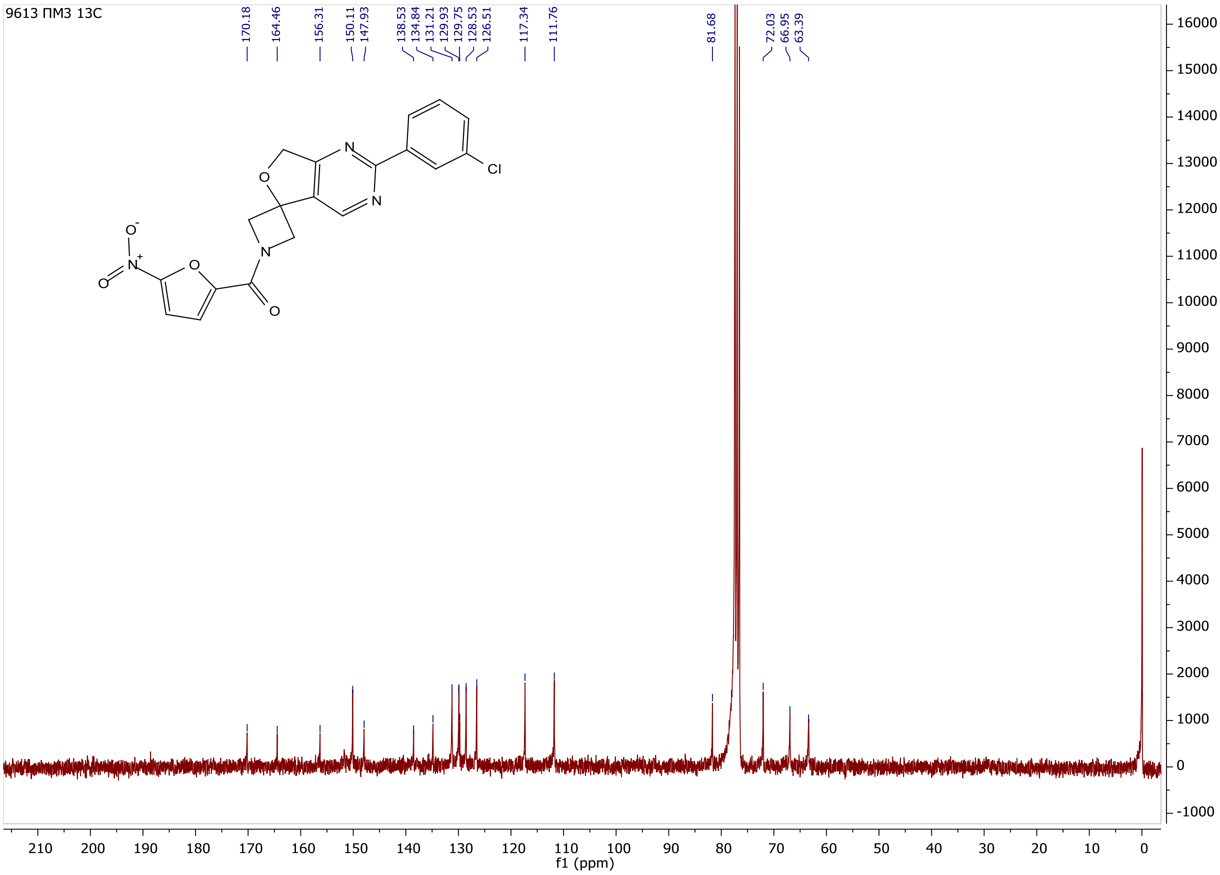

^{1}H and ^{13}C NMR spectra for compound 3h

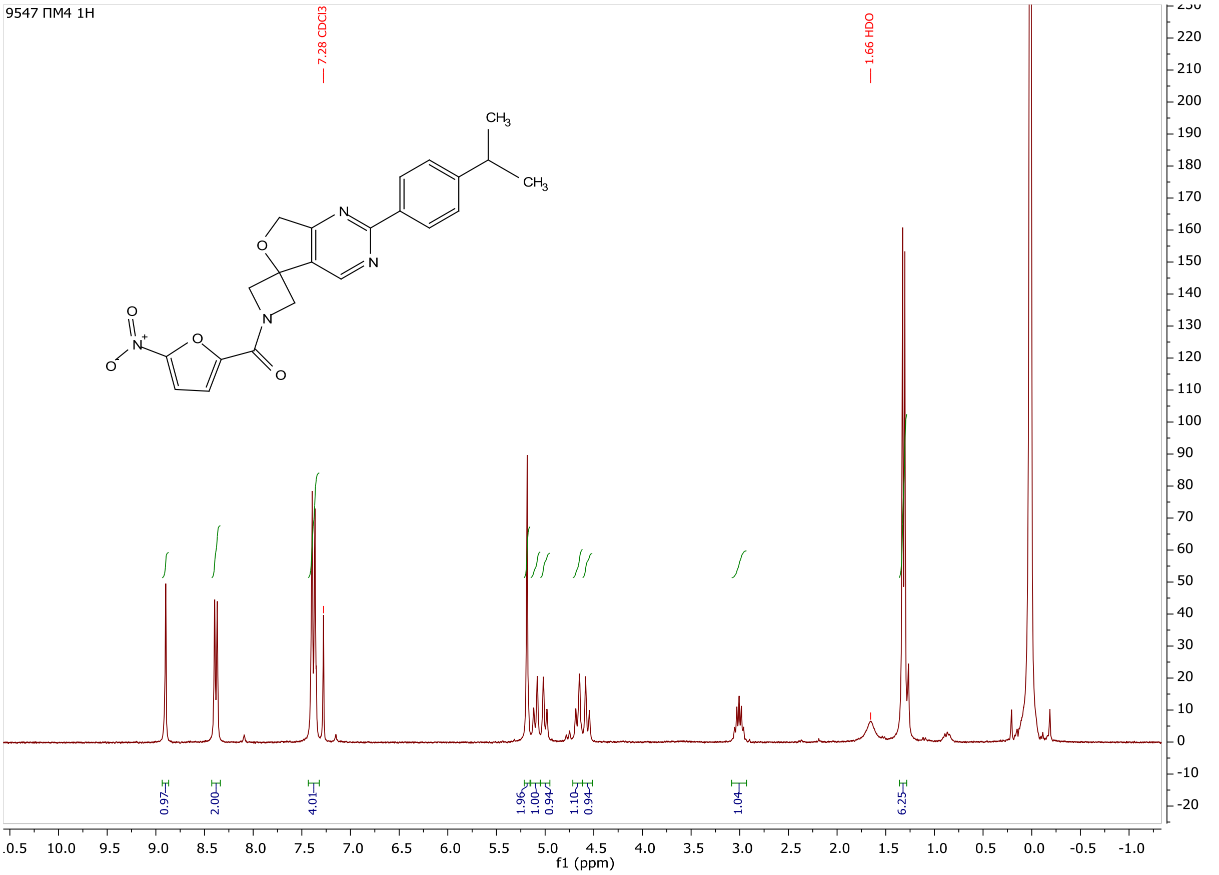

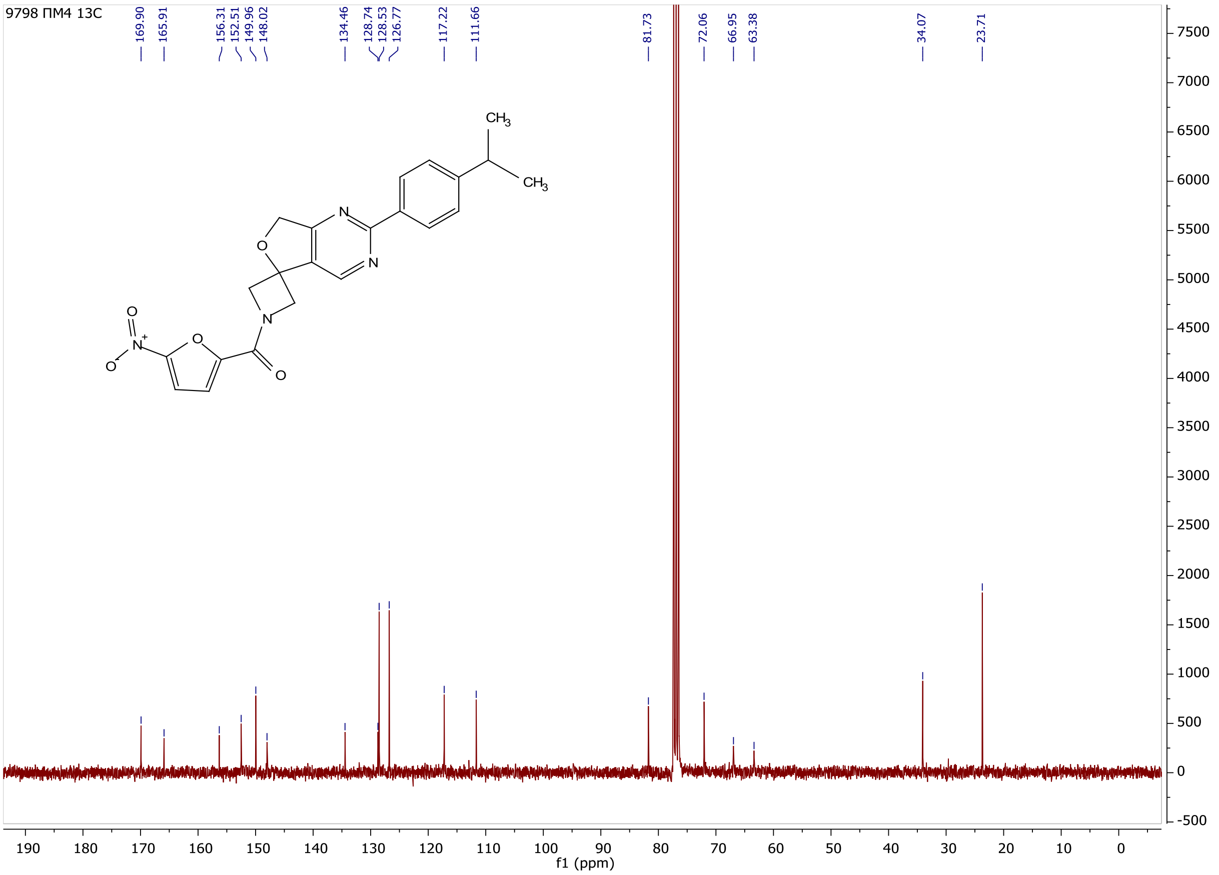

^{1}H and ^{13}C NMR spectra for compound 3i

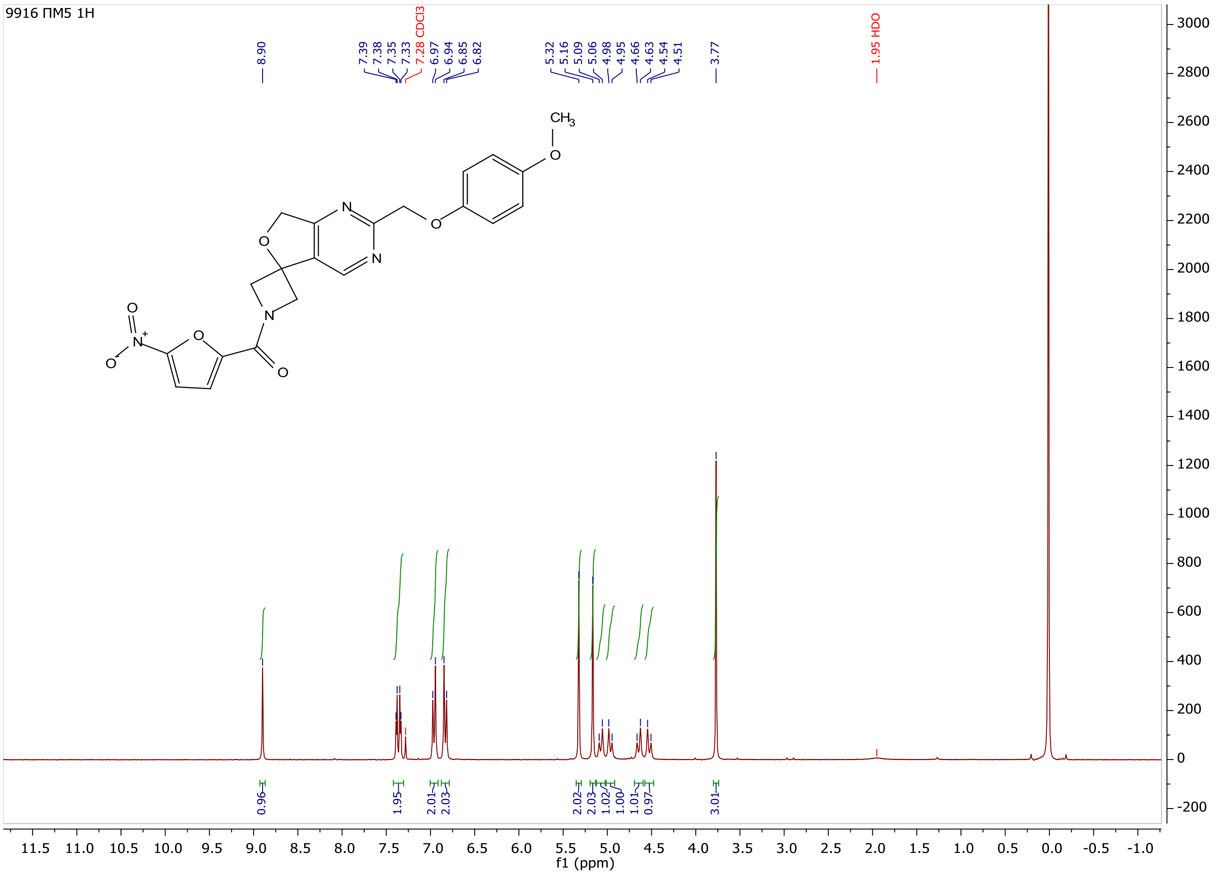

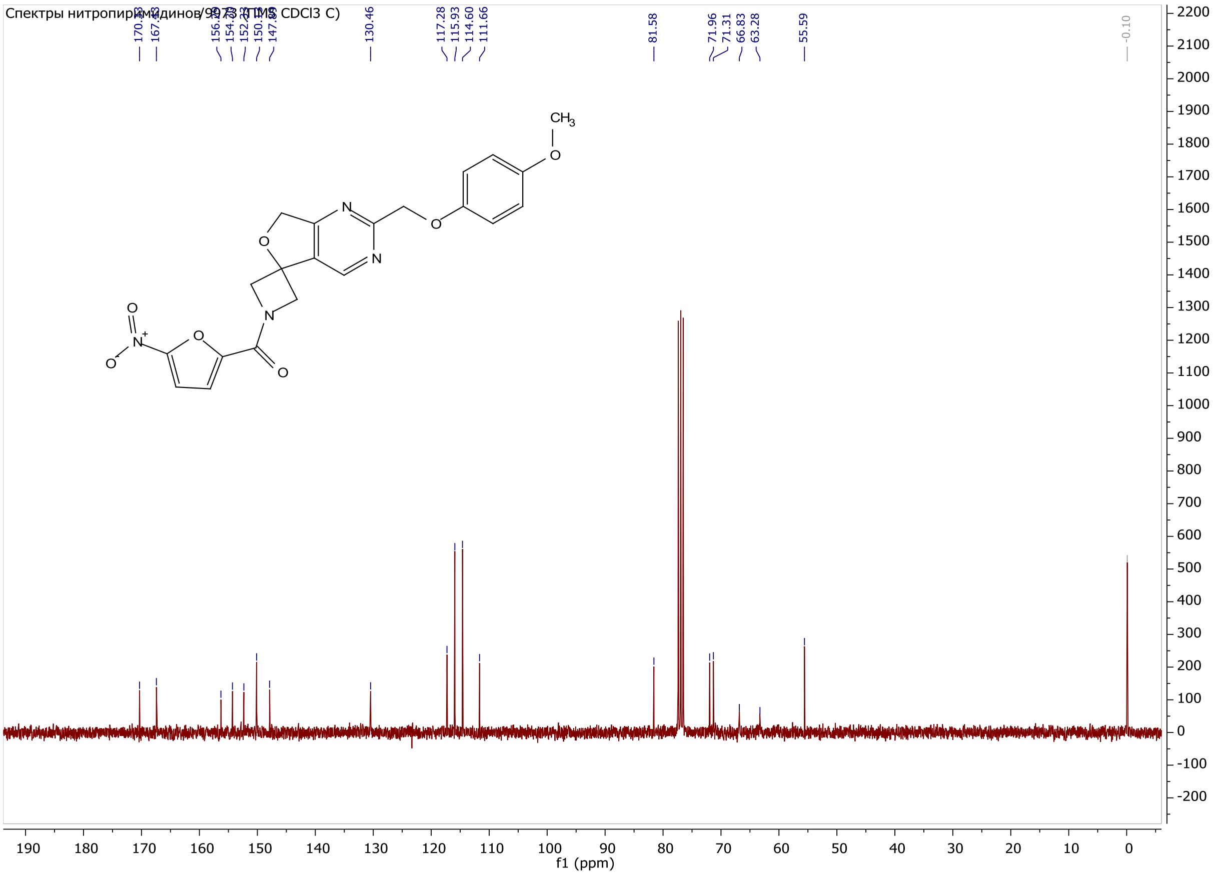

^1H and ^{13}C NMR spectra for compound 3j

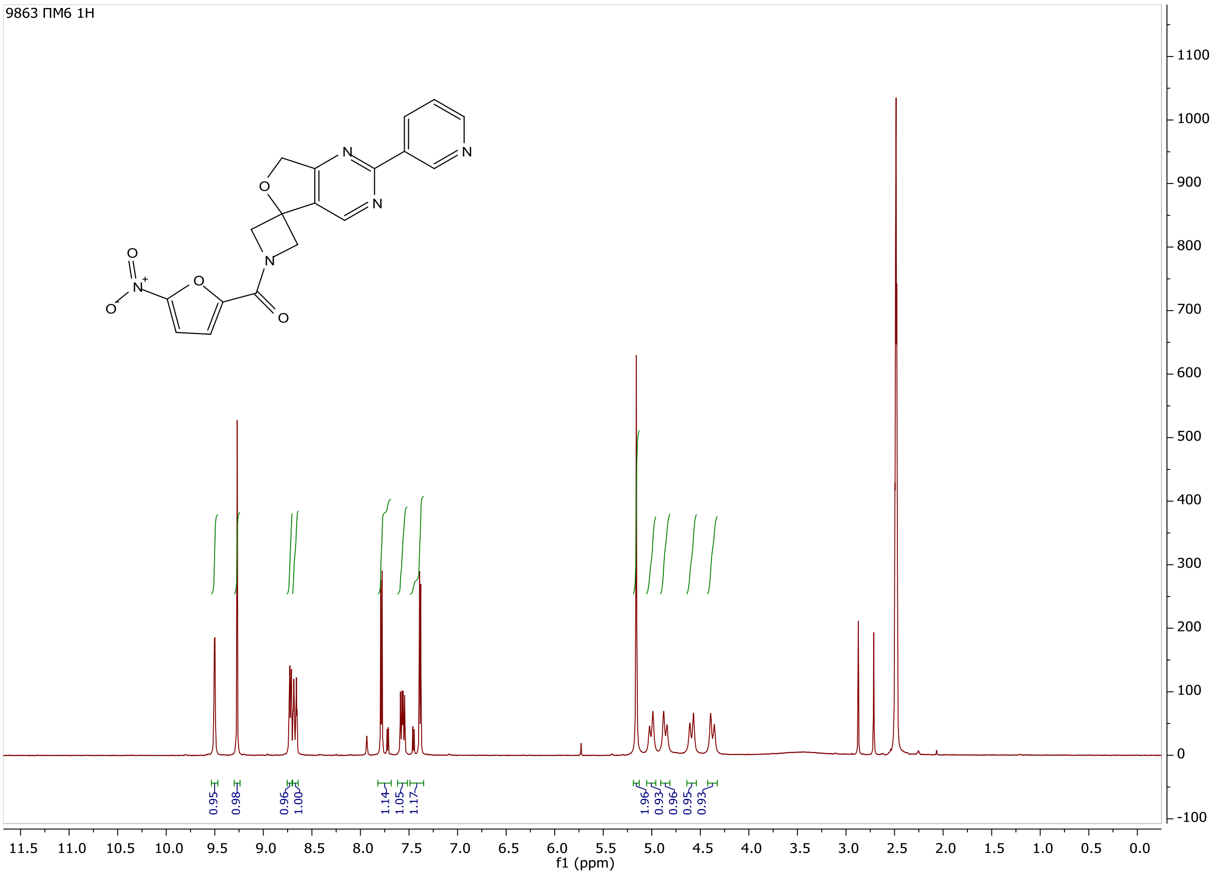

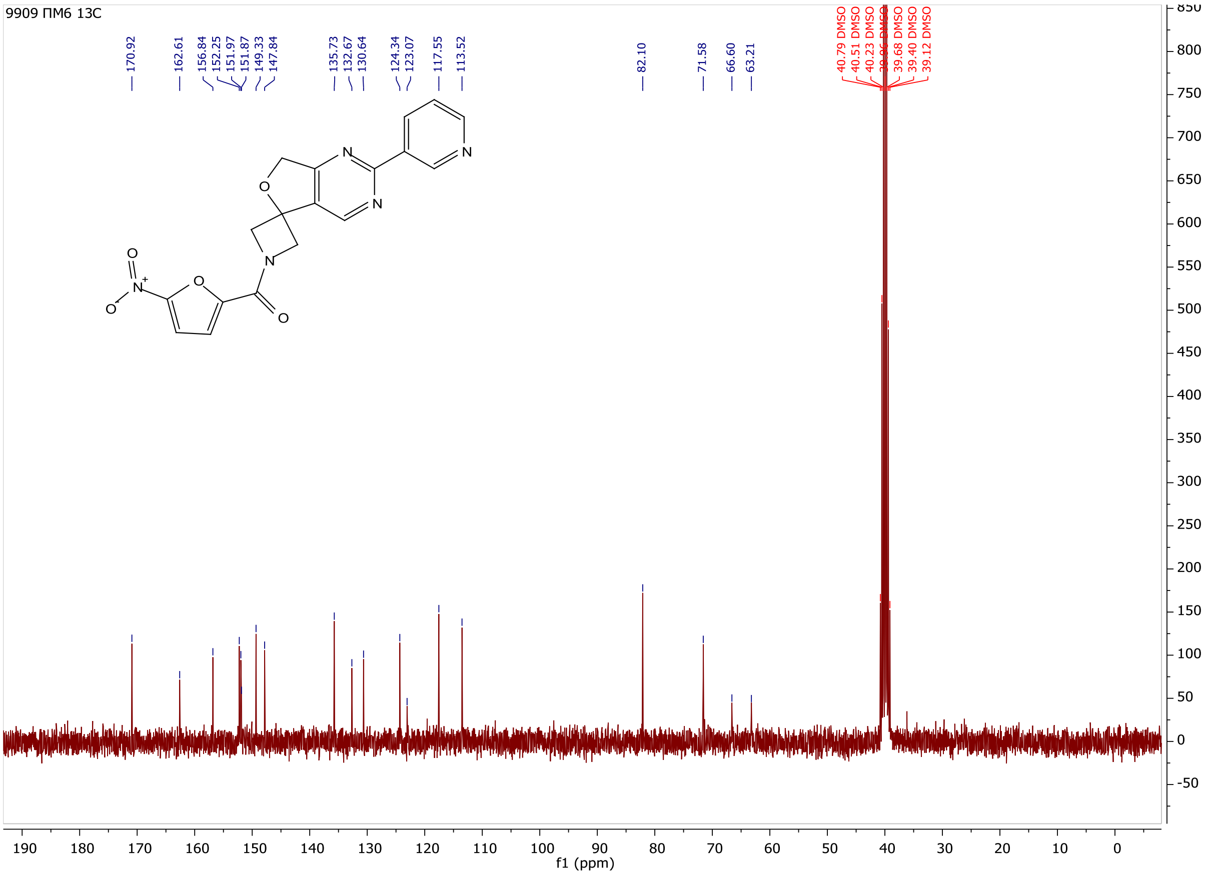

^{1}H and ^{13}C NMR spectra for compound 3k

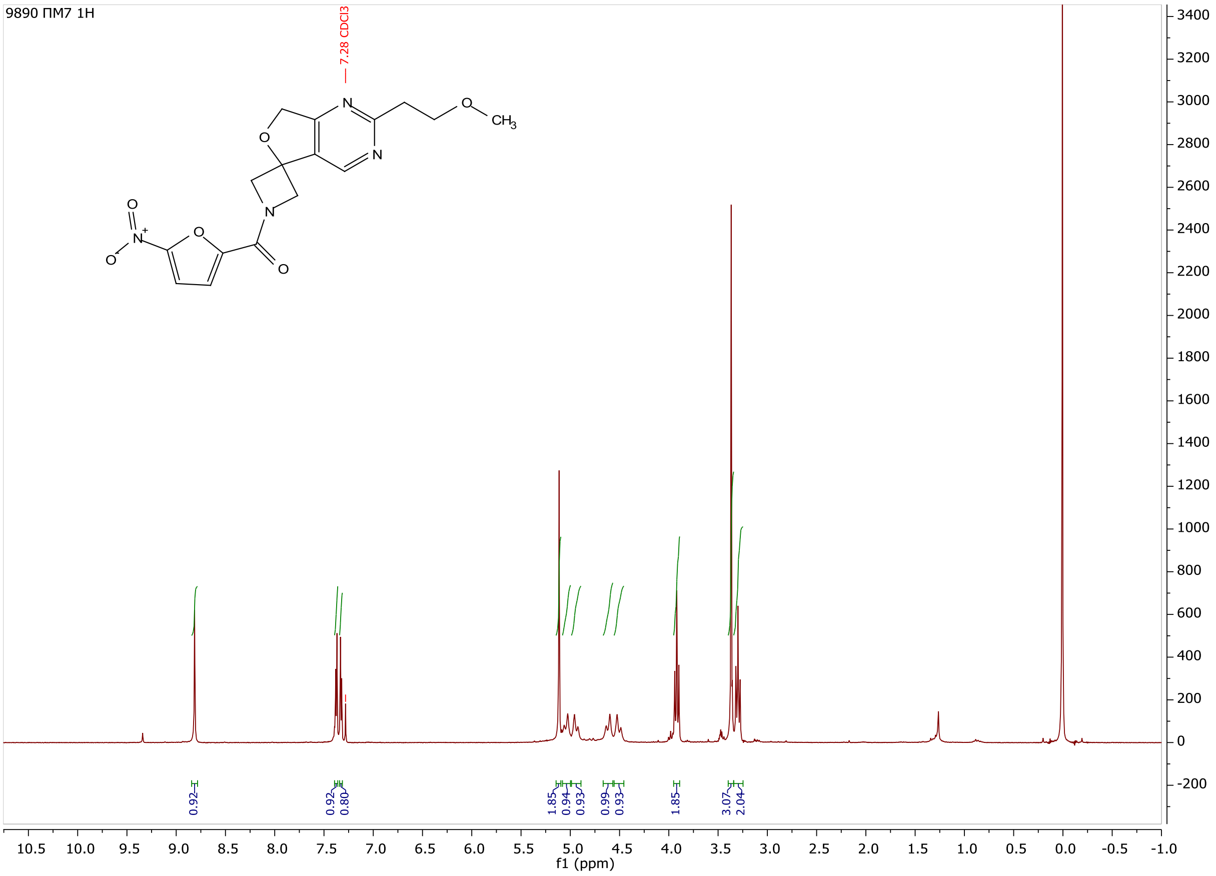

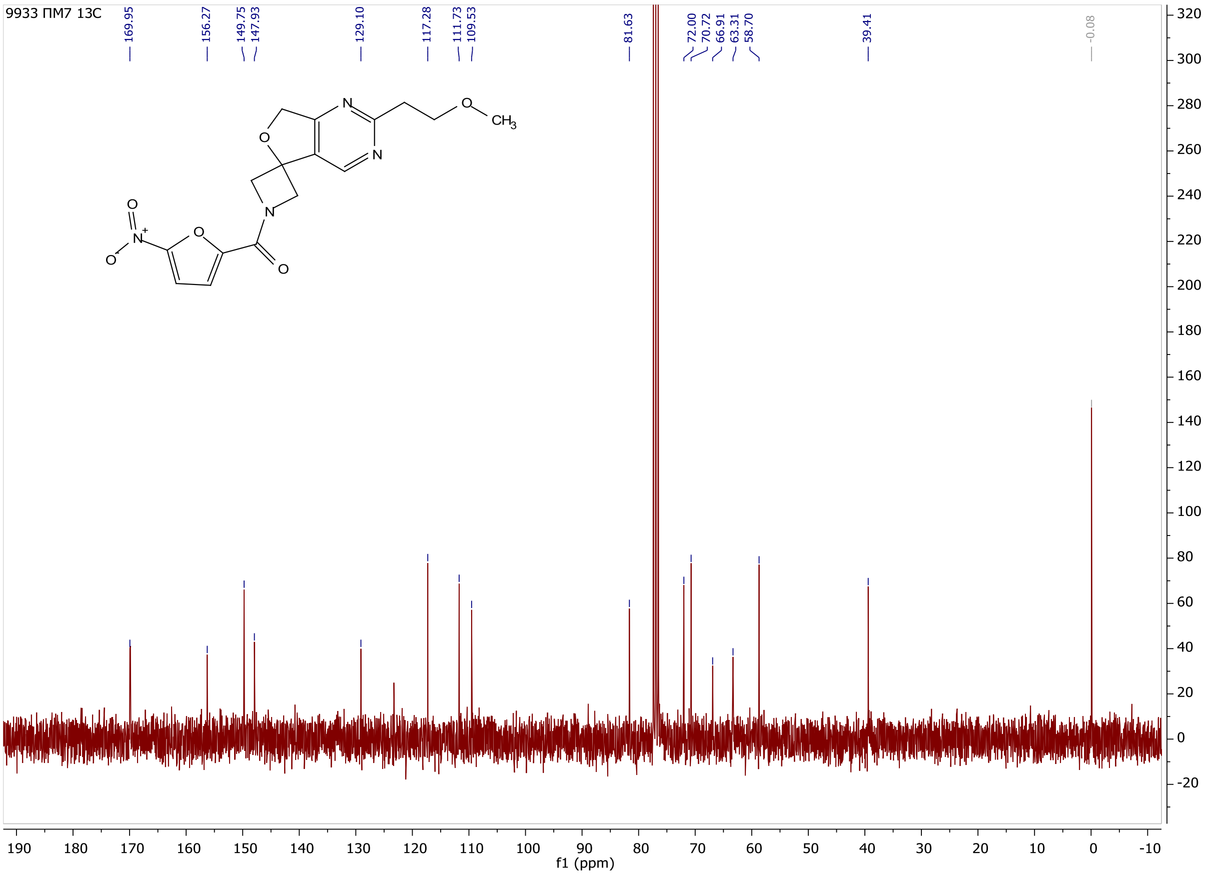

^{1}H and ^{13}C NMR spectra for compound 3l

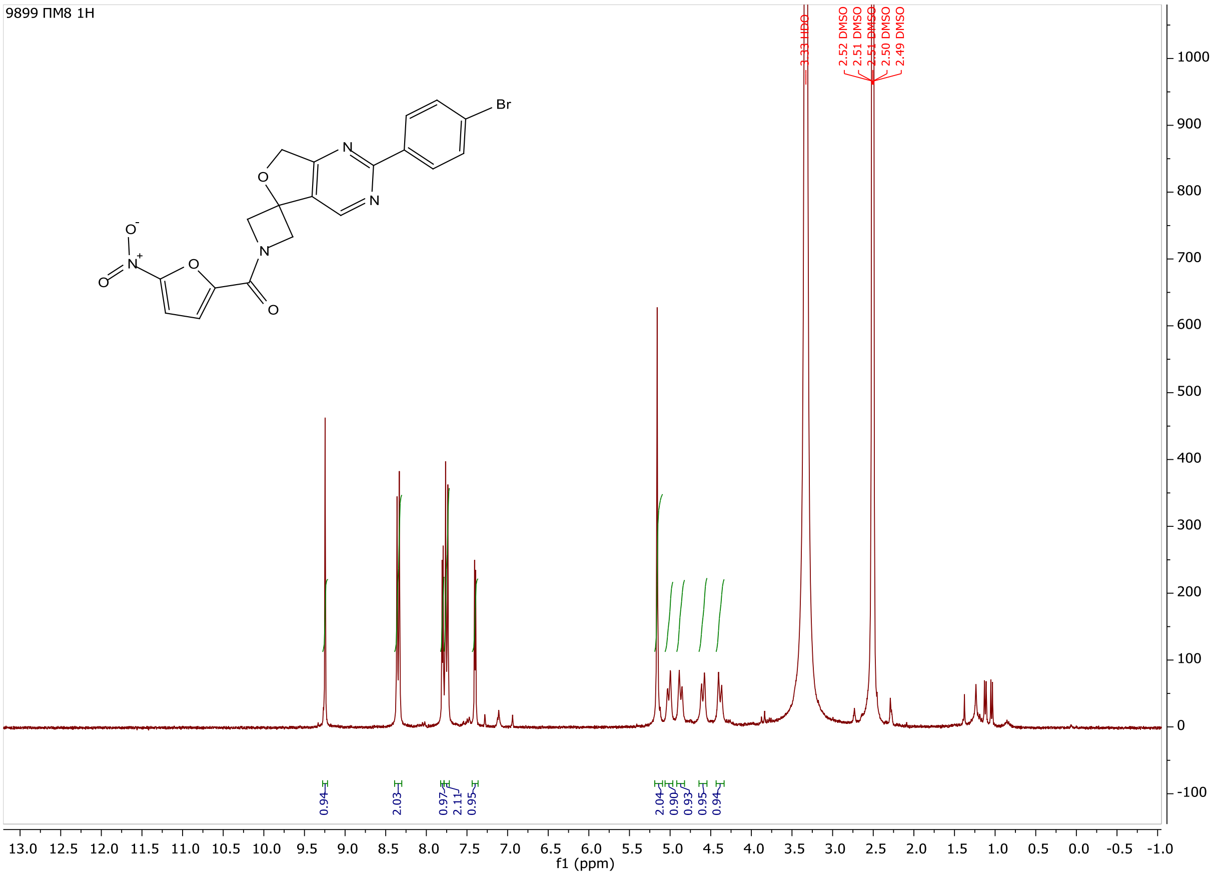

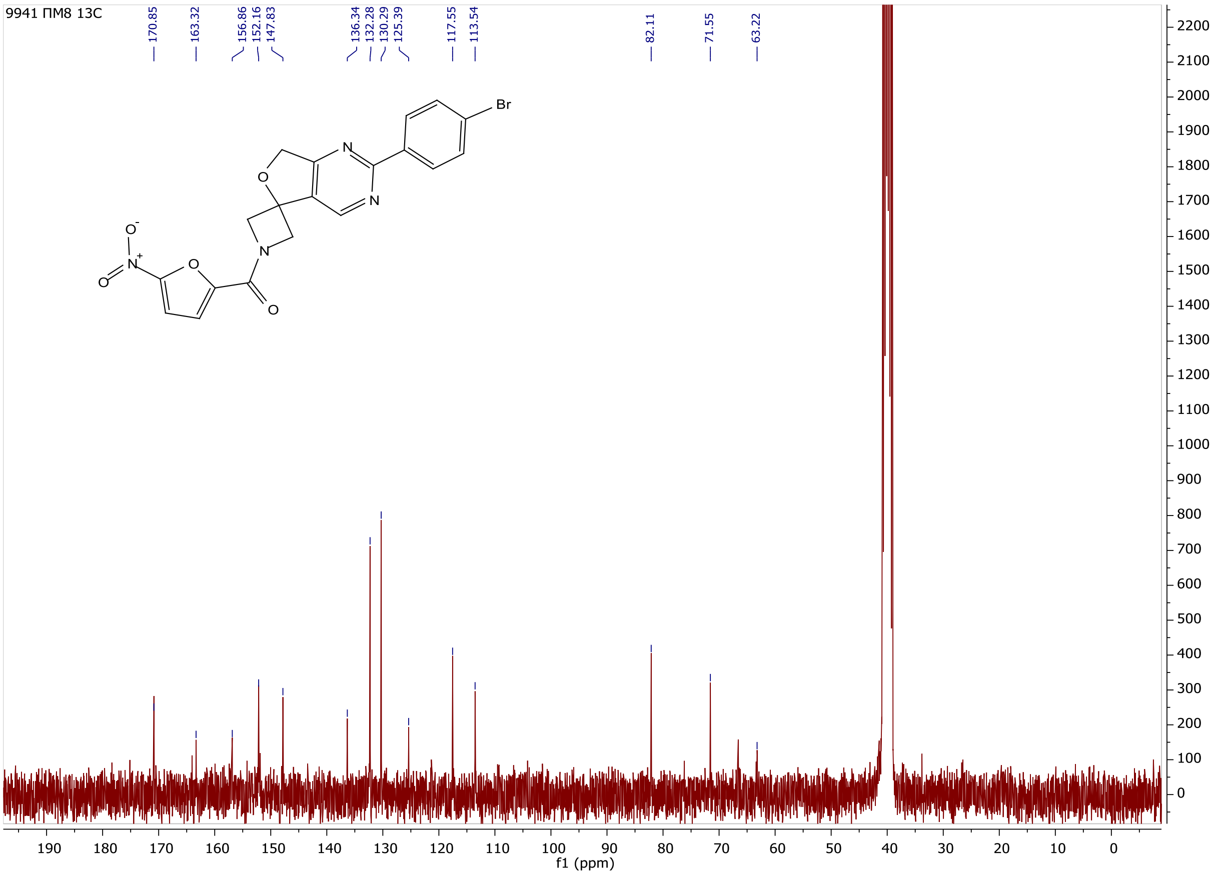

^{1}H and ^{13}C NMR spectra for compound 3m

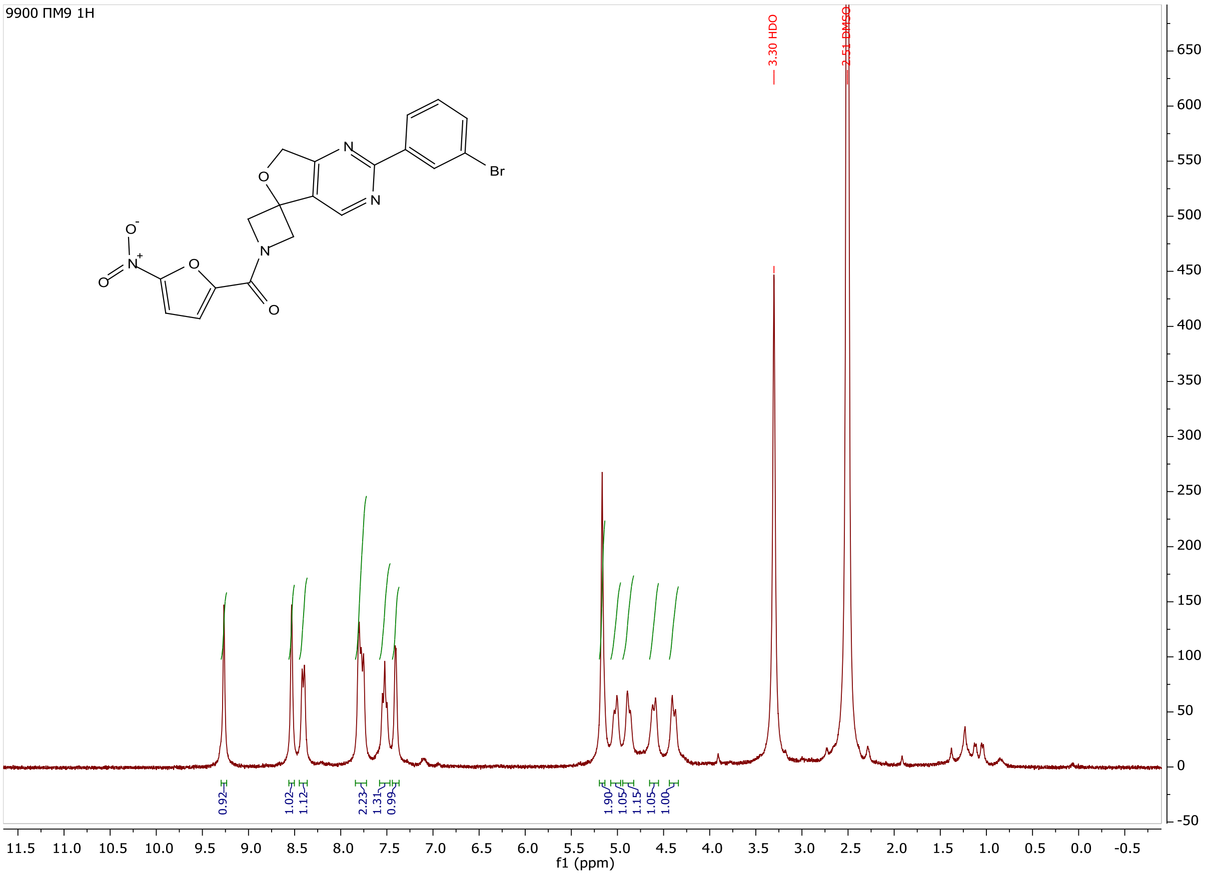

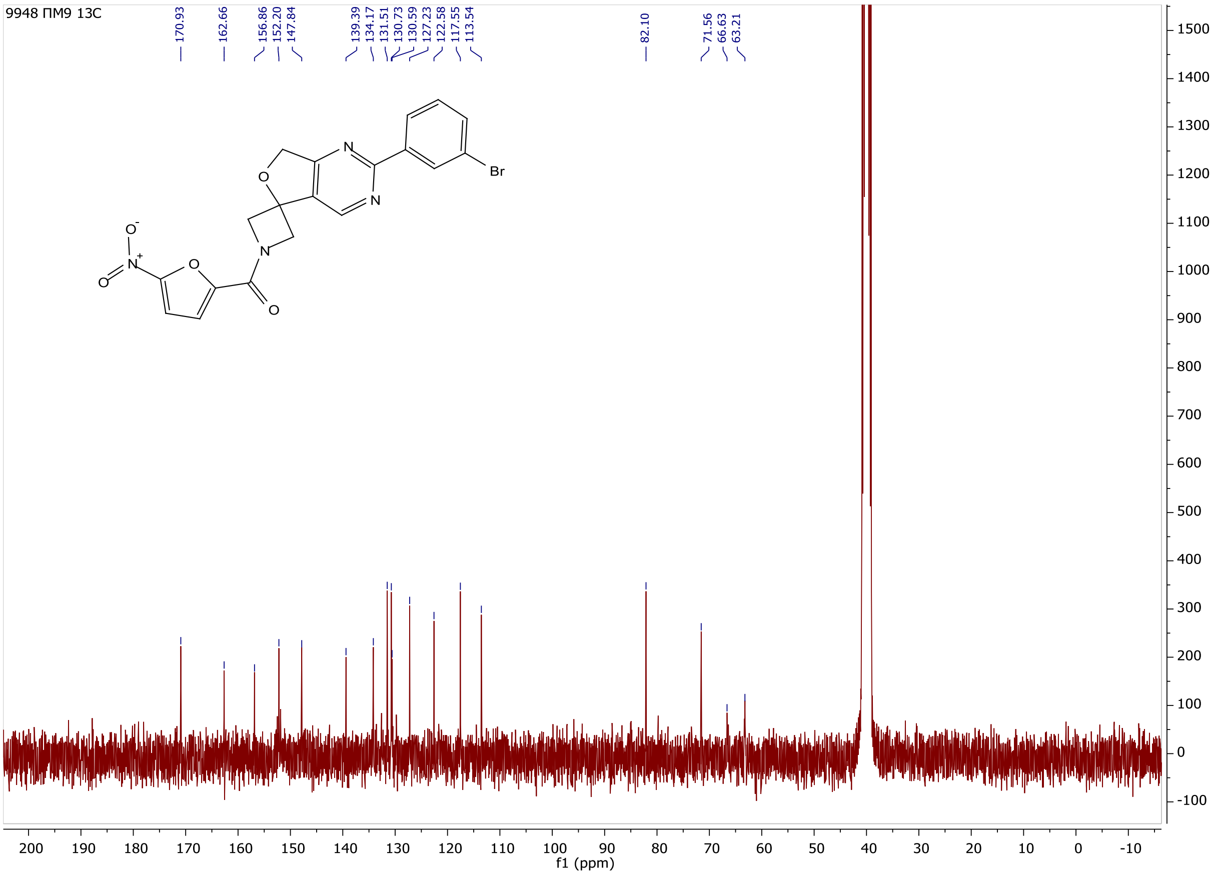

^{1}H and ^{13}C NMR spectra for compound 3n

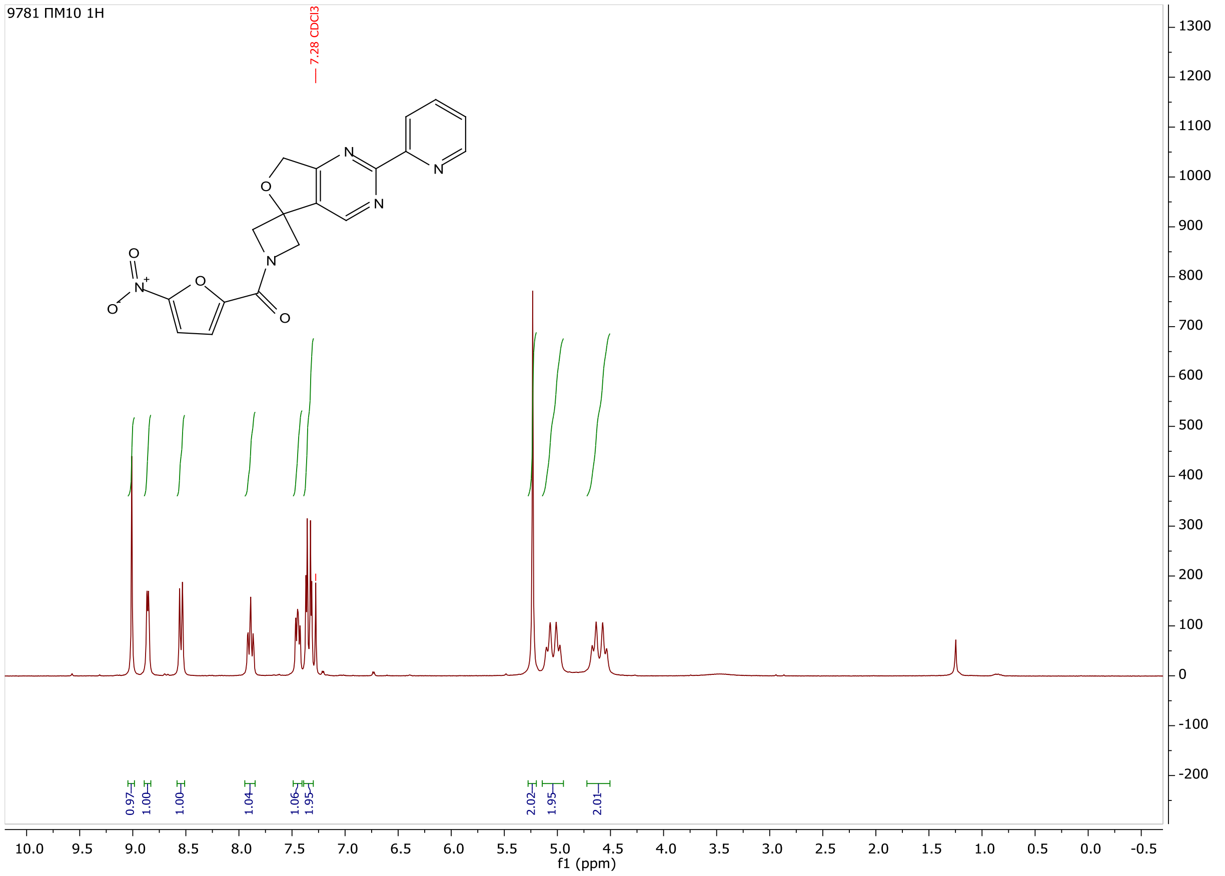

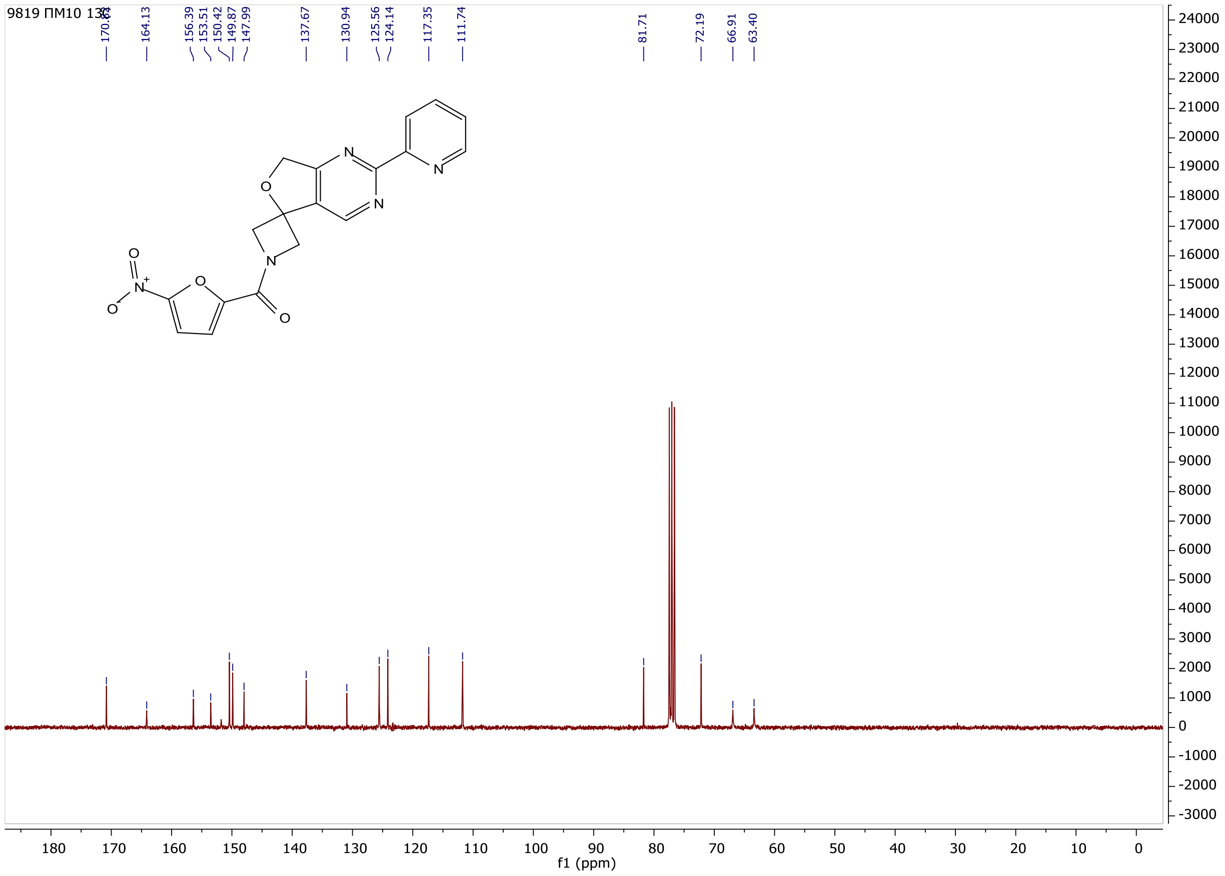

^{1}H and ^{13}C NMR spectra for compound 3o

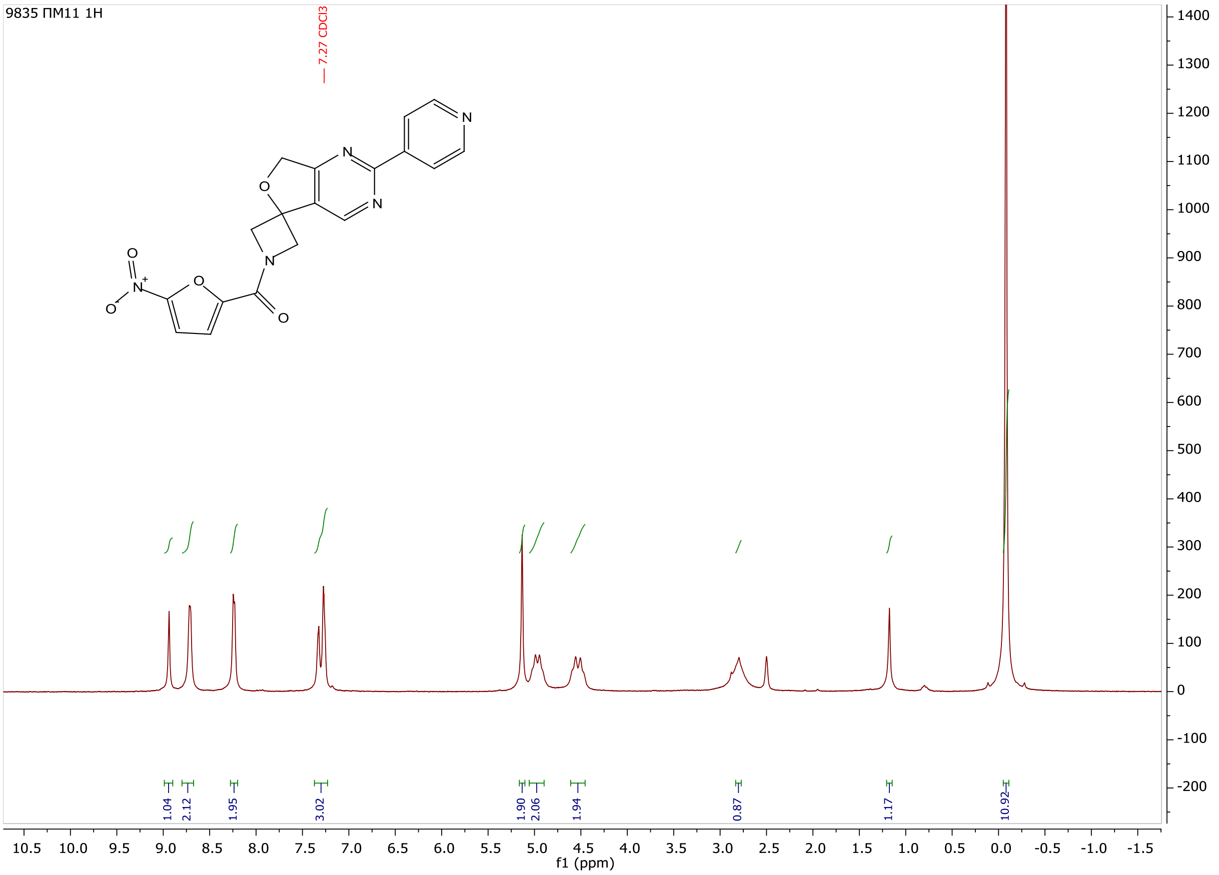

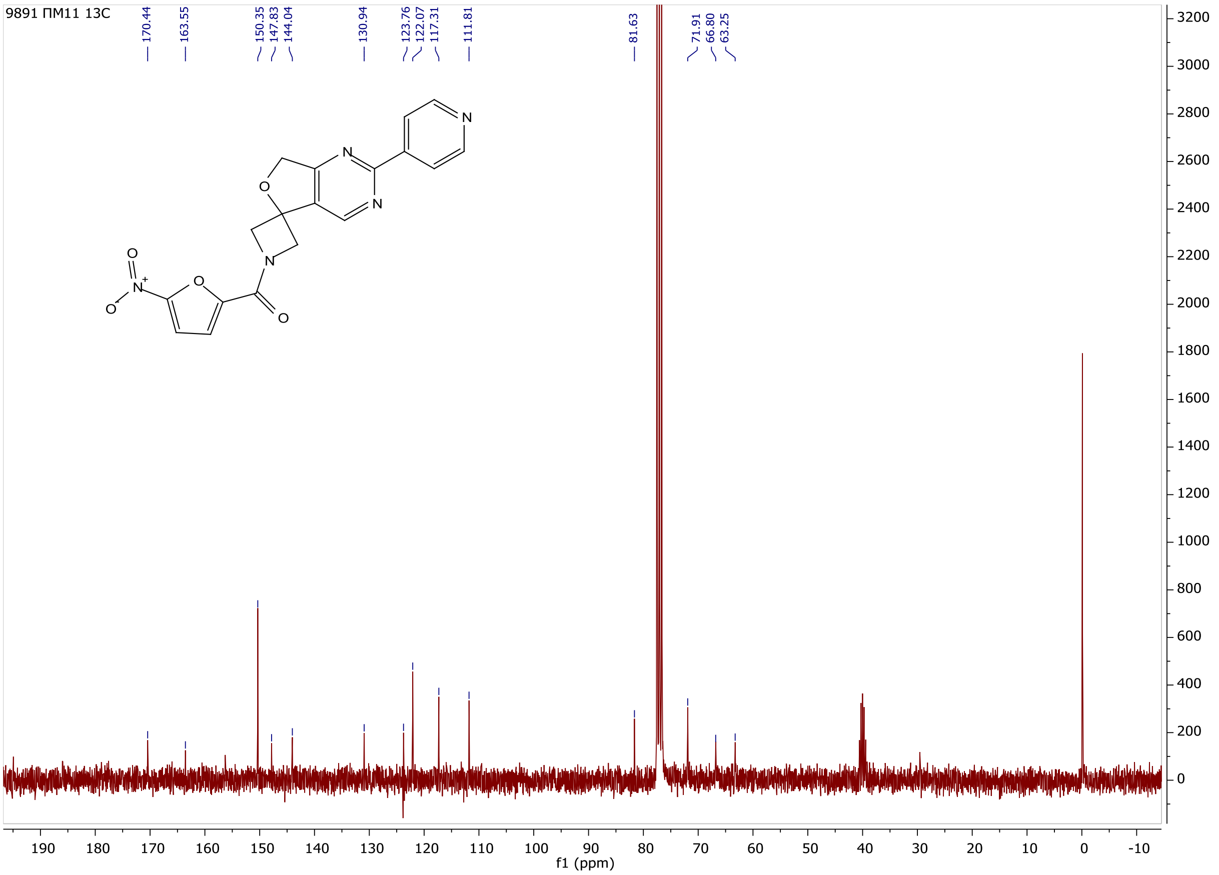

^{1}H and ^{13}C NMR spectra for compound 3p

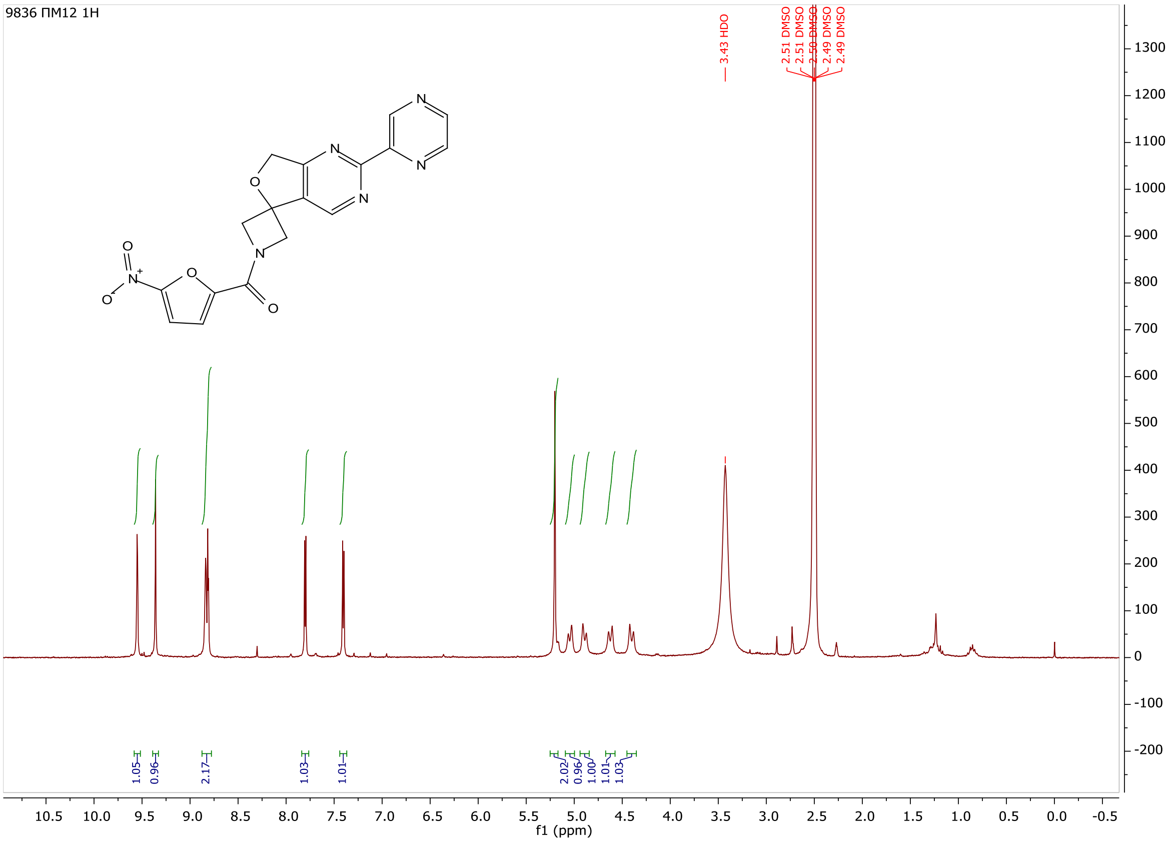

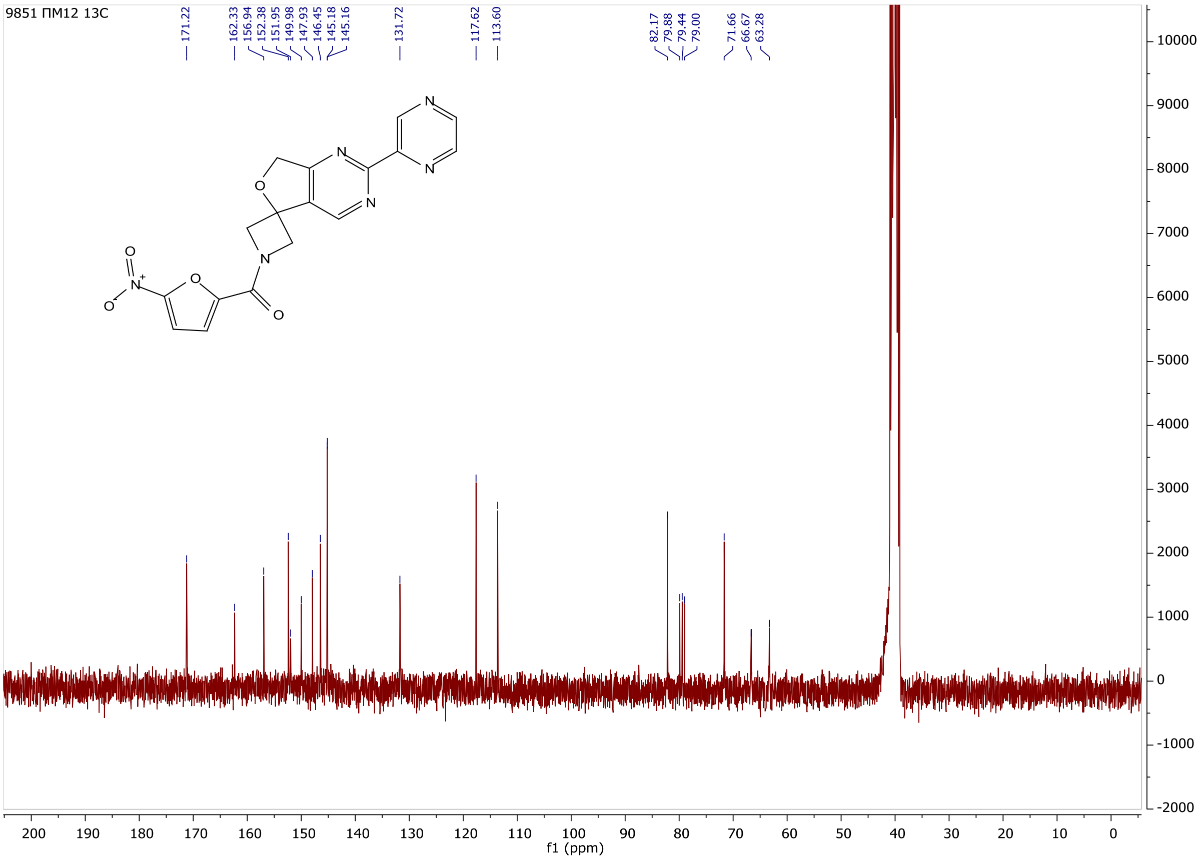

^{1}H and ^{13}C NMR spectra for compound 3q

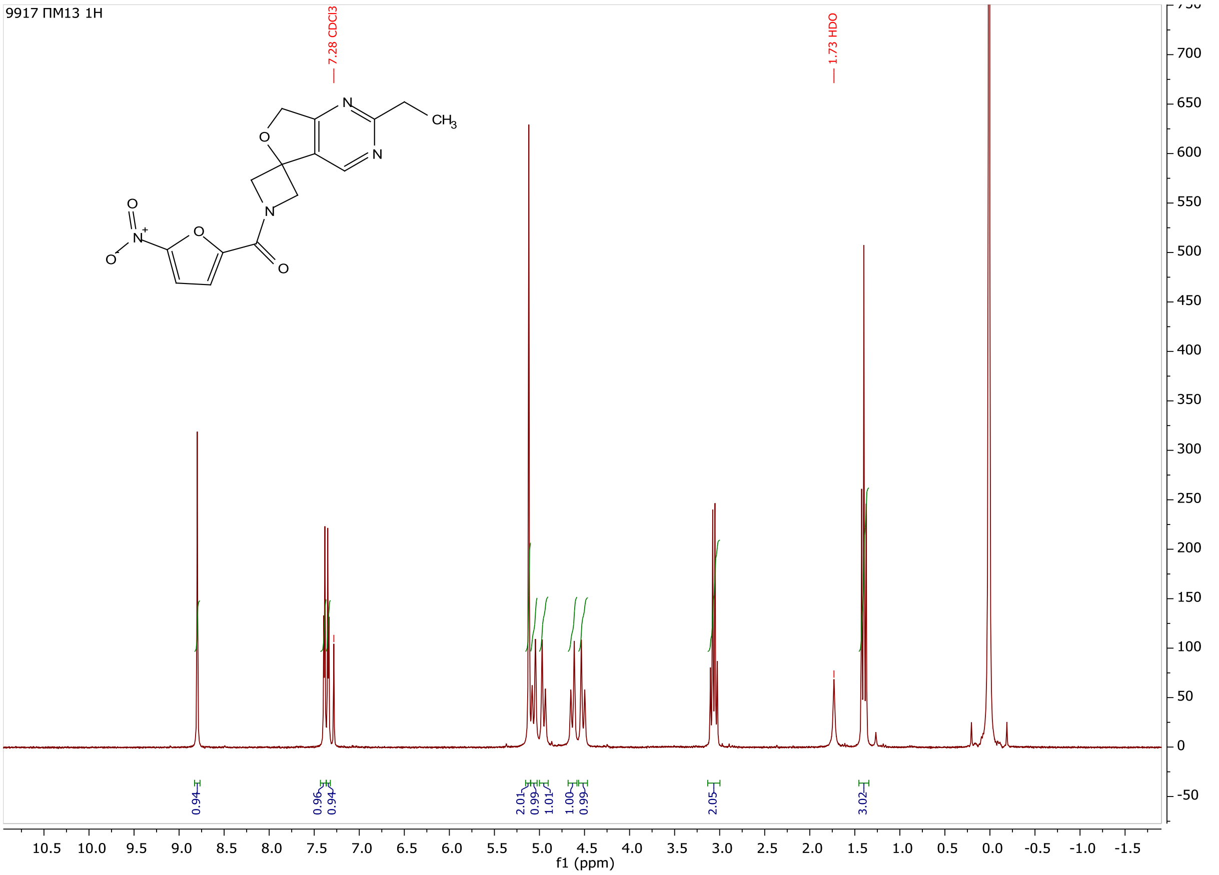

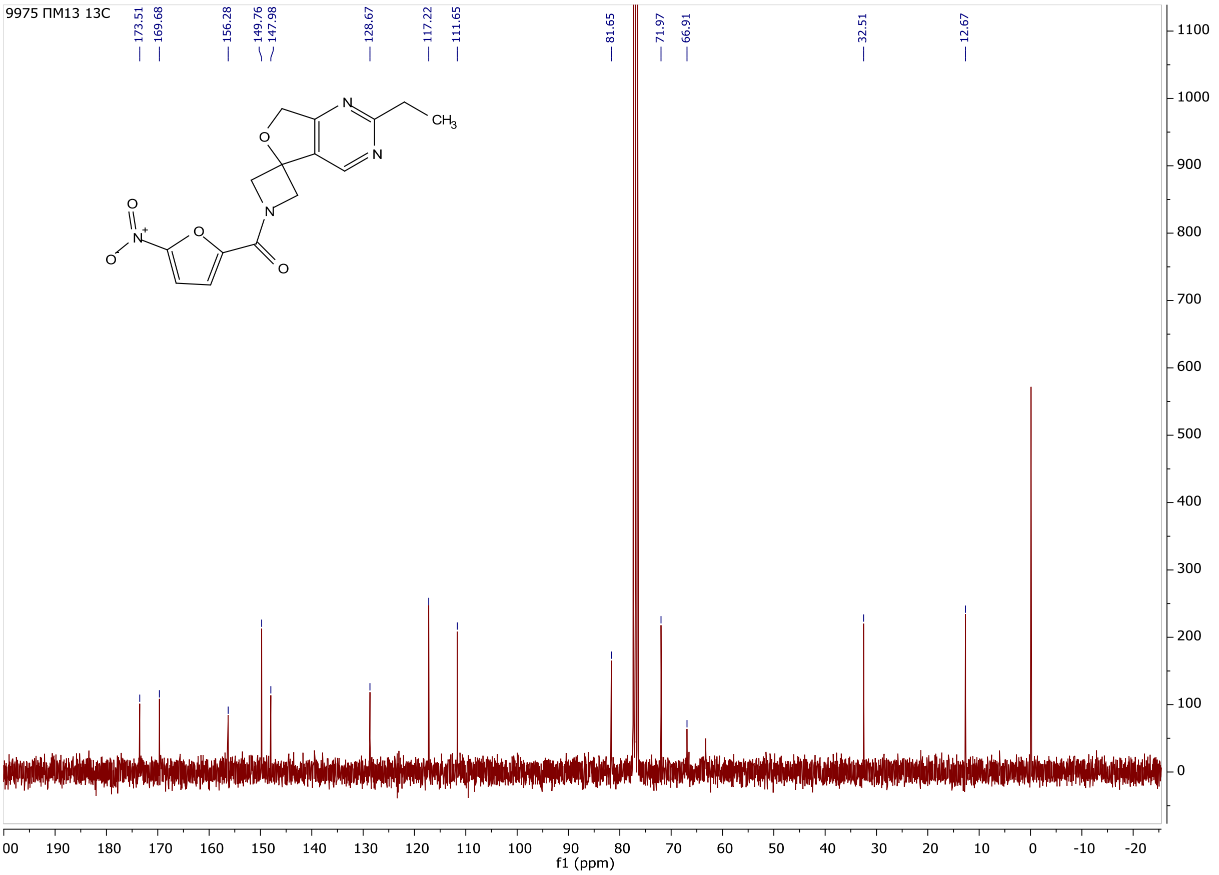

^{1}H and ^{13}C NMR spectra for compound 3r

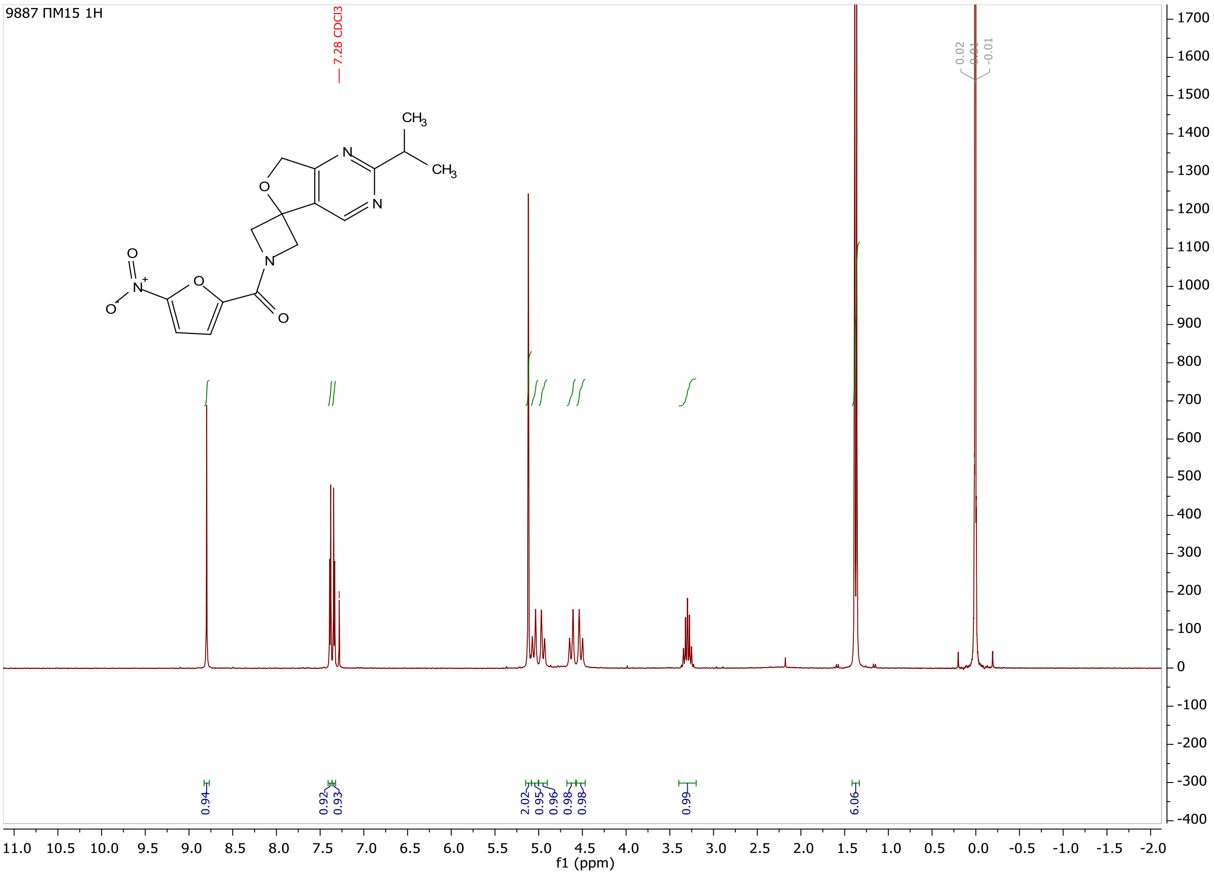

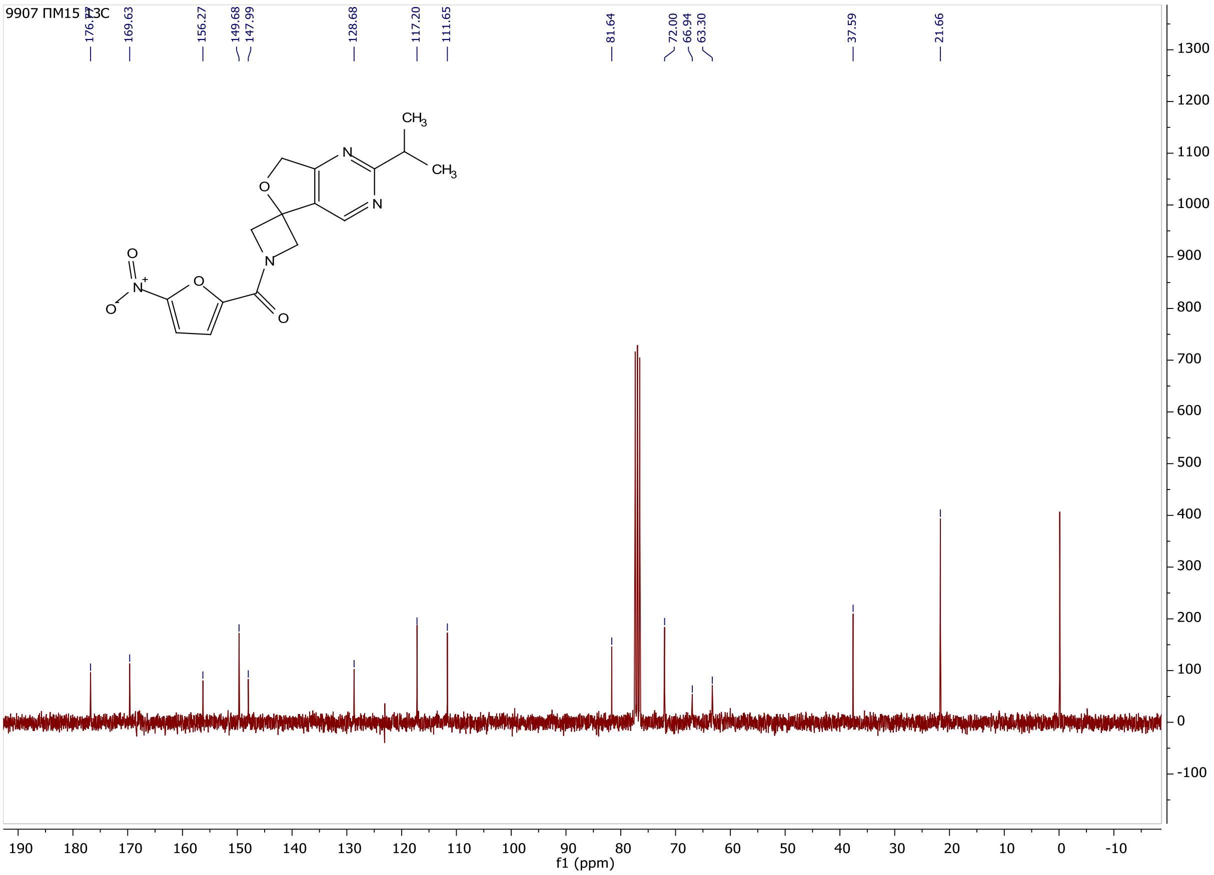

^{1}H and ^{13}C NMR spectra for compound 3u

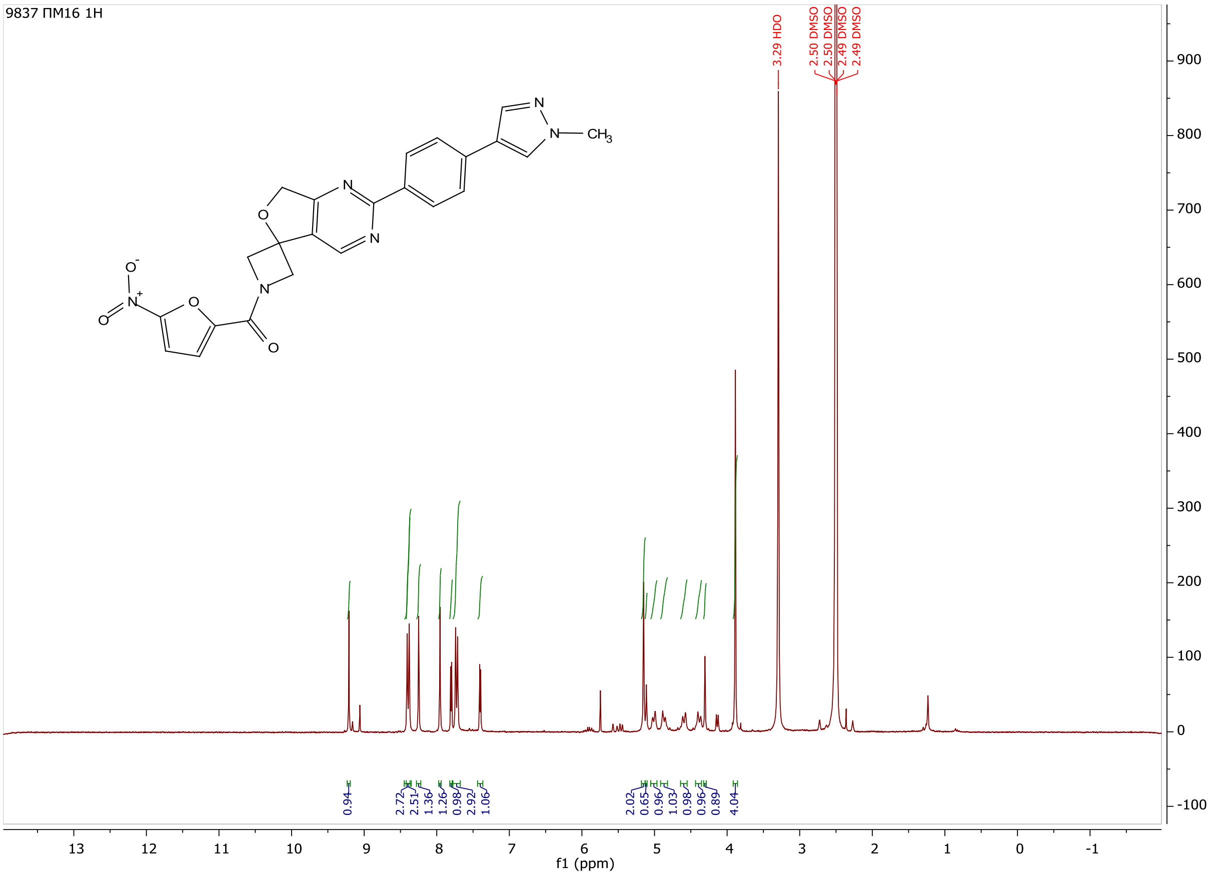

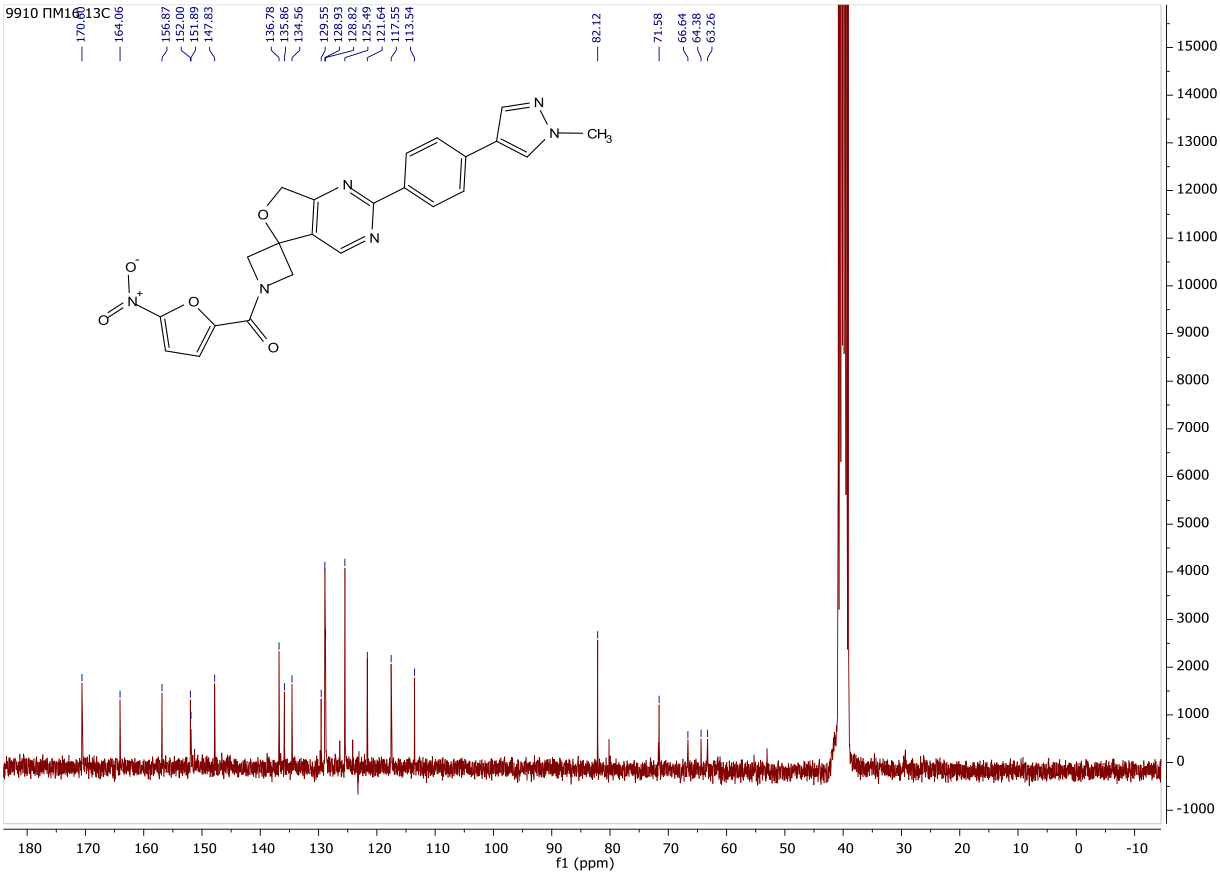

^{1}H and ^{13}C NMR spectra for compound 3t

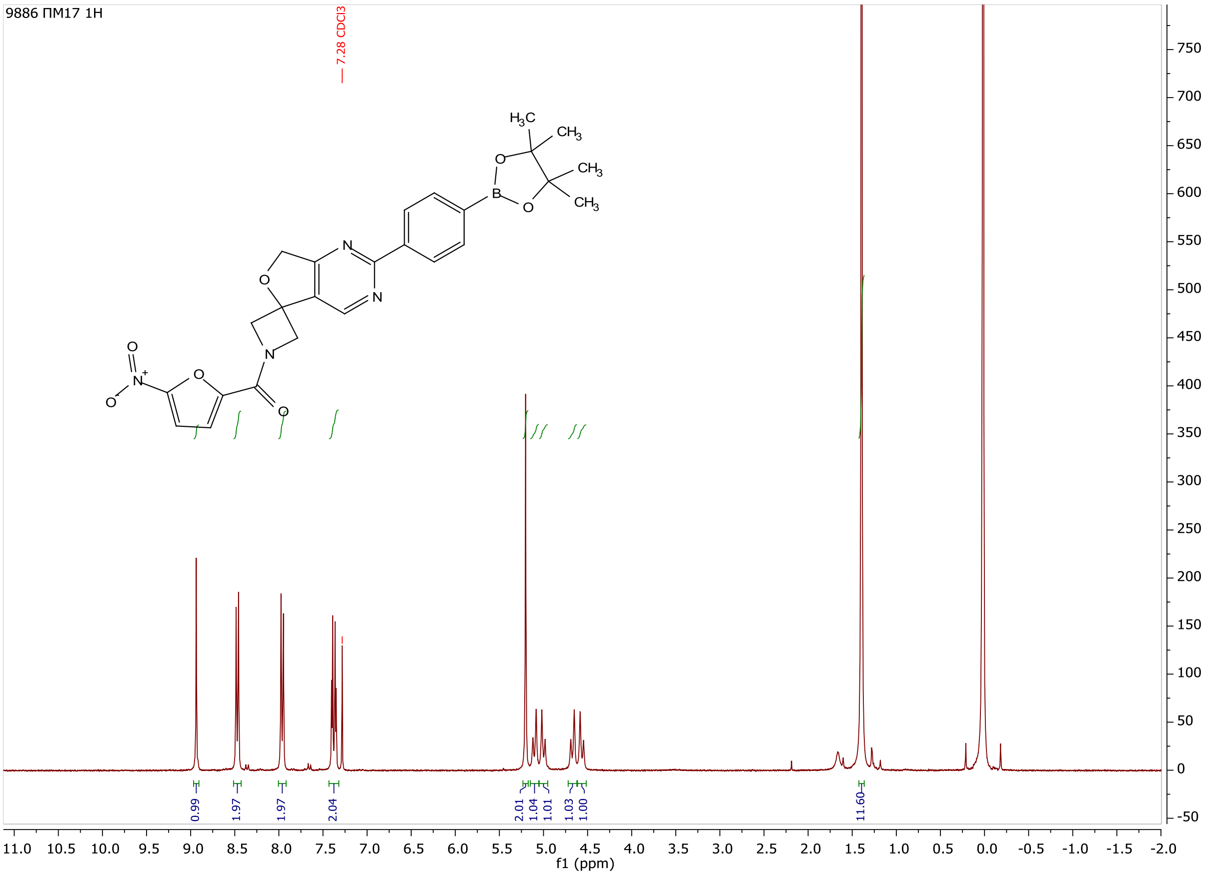

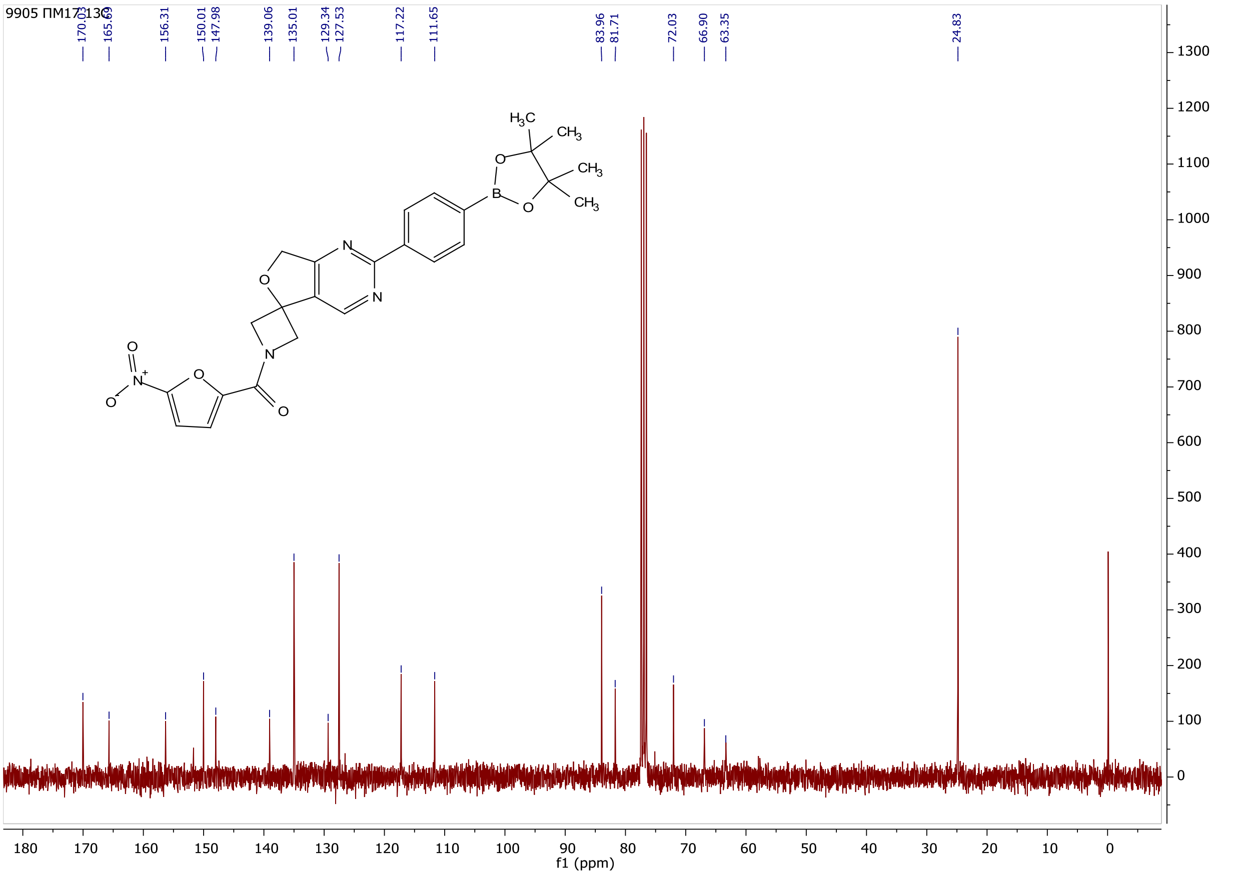

^{1}H and ^{13}C NMR spectra for compound 3s

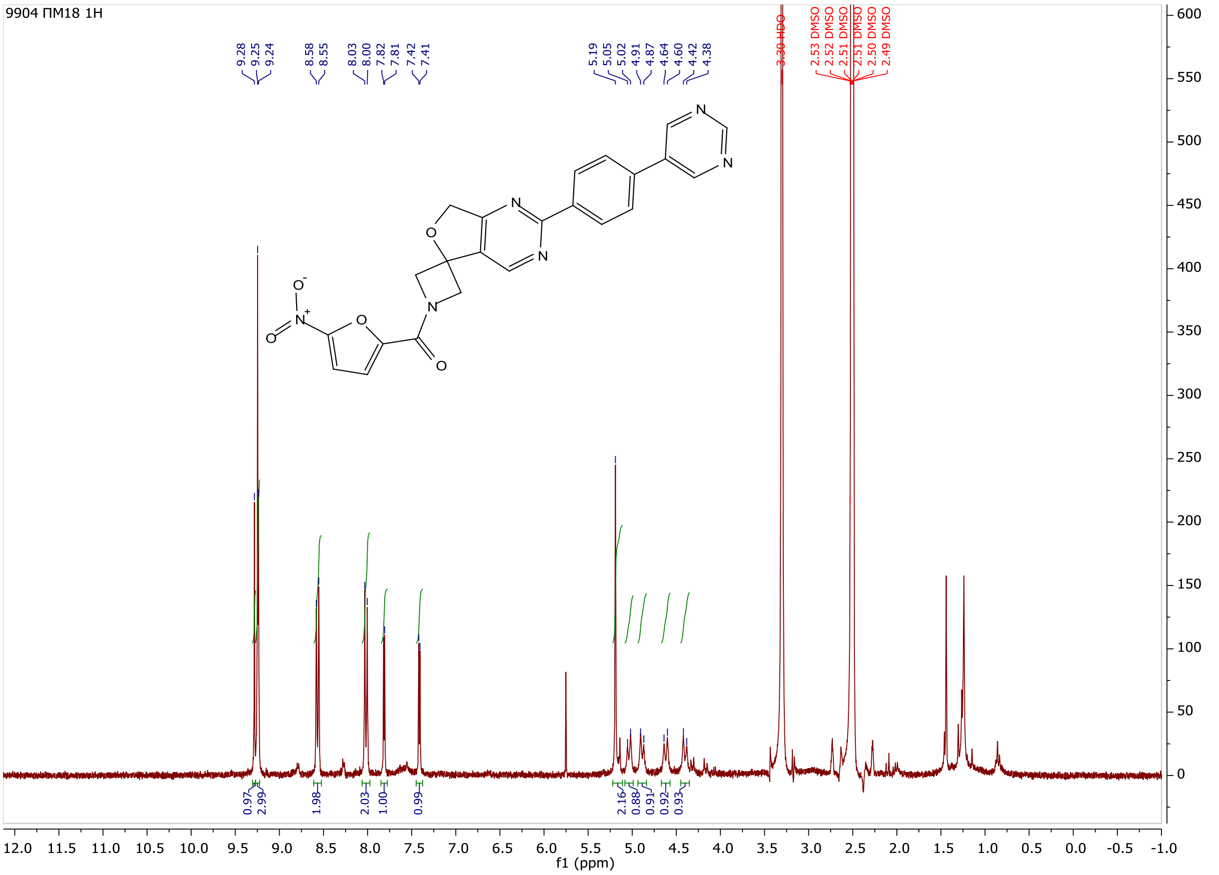

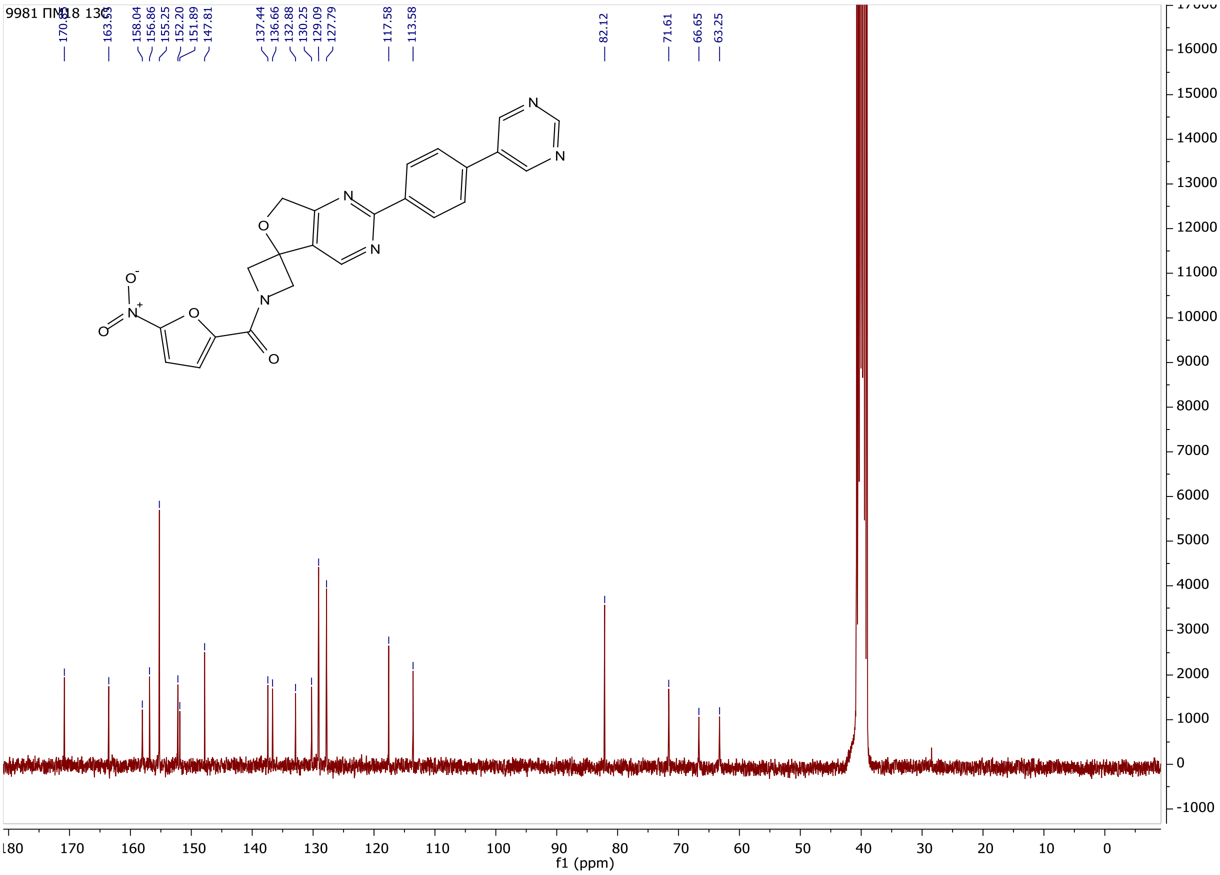

Supplement: Supplementary file 1 [file molecules-29-03071-s001.zip › molecules-3043702-supplementary.pdf]
